# FRET-Based Nanobiosensors for Imaging Intracellular Ca^2+^ and H^+^ Microdomains

**DOI:** 10.3390/s150924662

**Published:** 2015-09-23

**Authors:** Alsu I. Zamaleeva, Guillaume Despras, Camilla Luccardini, Mayeul Collot, Michel de Waard, Martin Oheim, Jean-Maurice Mallet, Anne Feltz

**Affiliations:** 1 Ecole Normale Supérieure, Institut de Biologie de l'ENS (IBENS), Inserm U1024, CNRS UMR 8197, Paris F-75005, France; E-Mails: alsu.zamaleeva@curie.fr (A.I.Z.); Camilla.luccardini@univ-lyon1.fr (C.L.); 2 Department de Chimie, École Normale Supérieure-PSL Research University, CNRS UMR 7203 LBM, 24, rue Lhomond, and Sorbonne University, UPMC Univ Paris 06 LBM, 4 place Jussieu, Paris F-75005, France; E-Mails: gdespras@oc.uni-kiel.de (G.D.); mayeul.collot@unistra.fr (M.C.); jean-maurice.mallet@ens.fr (J.-M.M.); 3 Inserm U836, Grenoble Neuroscience Institute, Research Group 3, LabEx Ion Channel Science and Therapeutics, Joseph Fourier University, BP170, Grenoble Cedex 09 38042, France; E-Mail: Michel.dewaard@ujf-grenoble.fr; 4 Brain Physiology Laboratory, CNRS UMR 8118, Faculté des Sciences Fondamentales et Biomédicales, Fédération de Neurosciences FR3636, Paris Descartes University, PRES Sorbonne Paris Cité, Paris F-75006, France; E-Mail: Martin.oheim@parisdescartes.fr

**Keywords:** quantum dot nanobiosensors, nanoparticle surface chemistry, FRET-based Ca^2+^ and H^+^ probes, red-emitting indicator, intracellular Ca^2+^ and H^+^ fluorometry, cell-penetrating peptide, concentration microdomain

## Abstract

Semiconductor nanocrystals (NCs) or quantum dots (QDs) are luminous point emitters increasingly being used to tag and track biomolecules in biological/biomedical imaging. However, their intracellular use as highlighters of single-molecule localization and nanobiosensors reporting ion microdomains changes has remained a major challenge. Here, we report the design, generation and validation of FRET-based nanobiosensors for detection of intracellular Ca^2+^ and H^+^ transients. Our sensors combine a commercially available CANdot^®^565QD as an energy donor with, as an acceptor, our custom-synthesized red-emitting Ca^2+^ or H^+^ probes. These ‘Rubies’ are based on an extended rhodamine as a fluorophore and a phenol or BAPTA (1,2-bis(o-aminophenoxy)ethane-*N*,*N*,*N*′,*N*′-tetra-acetic acid) for H^+^ or Ca^2+^ sensing, respectively, and additionally bear a linker arm for conjugation. QDs were stably functionalized using the same SH/maleimide crosslink chemistry for all desired reactants. Mixing ion sensor and cell-penetrating peptides (that facilitate cytoplasmic delivery) at the desired stoichiometric ratio produced controlled multi-conjugated assemblies. Multiple acceptors on the same central donor allow up-concentrating the ion sensor on the QD surface to concentrations higher than those that could be achieved in free solution, increasing FRET efficiency and improving the signal. We validate these nanosensors for the detection of intracellular Ca^2+^ and pH transients using live-cell fluorescence imaging.

## 1. Introduction

Microdomains of intracellular ion concentrations are central to the compartmentalization and specificity of cellular signalling pathways. For example, pH is strictly regulated in cytosol and is different in intracellular organelles, with an important impact on their respective functions: pH changes along the endo-lysosomal pathway directly affect ion fluxes and organelle function [1,2]. Likewise, cell energy supply is linked to the mitochondrial proton pump [3] and pH gradients control antigen processing and antigen presentation in the endocytic compartment of dendritic cells [4,5].On the other hand, the free cytoplasmic calcium ion concentration ([Ca^2+^]_i_) is a key factor regulating events as diverse as gene expression, cellular metabolism, mitochondrial mobility and exocytosis. In the case of Ca^2+^, the spatial restriction of subcellular Ca^2+^ gradients results from the combination of a localized and finely tuned Ca^2+^ entry across the plasma membrane (or from the release of Ca^2+^ from intracellular Ca^2+^ stores) and the strong cellular Ca^2+^ buffering capacity, limiting the subsequent diffusion by Ca^2+^-binding to mobile and fixed Ca^2+^ buffers [6]. However, in either case the precise spatio-temporal characterization of such microdomain signaling events with fluorimetric ion indicators has been difficult because the measurement of the local ion concentrations is hampered by the rapid diffusion of the ion and the indicator, the rapidity of the event, the small spatial extent of the microdomain and the relatively low number of ions and photons involved.

To better meet the conditions of ionic microdomain detection, we propose here the use of a fluorescent and multi-functionalized nanoparticle, carrying several ion sensor molecules as well as cell penetrating peptides (CPPs) to facilitate its cytoplasmic delivery. We used a commercial quantum dot (QD), *i.e.*, an inorganic colloïdal fluorescent particle as a central scaffold for our biosensor. The broad absorption spectrum, the well-defined symmetric emission spectrum and the large brightness and high resistance to photobleaching compared to small-molecule organic fluorophores make QDs good energy donors for fluorescence resonance energy transfer (FRET) and facilitate the detection of single QDs inside live cells. Several organic ion indicator molecules linked to the QD surface act as fluorescent energy acceptors. The FRET efficiency of this assembly is given by the overlap of the emission spectrum of the QD with the absorbance spectrum of the fluorescent ion indicator, as well as their number, orientation and proximity with the QD surface.

These hybrid organic/inorganic nanobiosensors can be used, by virtue of the stable green fluorescence of the QD, to firstly identify candidate sites of H^+^ or Ca^2+^ accumulation and thereby “tag and track” the local ion concentration in this very location by reading out the spectrally distinct fluorescence of the ion indicator [7,8,9]. Analyte sensing can result either from an ion-binding dependent change in the acceptor absorption, or an ion-binding dependent change in the acceptor fluorescence quantum yield, or from an analyte-induced change in the donor/acceptor distance or relative orientation. In the case of the hereby-used CANdot@565 QDs and our CaRuby indicators ([8] and Figure S1), the acceptor displays no spectral shift upon analyte binding, as the Rubies emission spectra are changing only in the amplitude but not in shape with analyte concentration. Squaraine- [10] and fluorescein-base pH sensors [11] have been reported as well but while the former has a poor quantum yield the latter emit in the yellow-green spectral band, making them both incompatible for pH measurements in green-fluorescent-protein (GFP) expressing cells. Unlike these “intensometric” dyes, seminaphtorhodafluors (SNARF-dyes), routinely used pH sensors [12,13] display a shift of both the absorption and emission spectra following proton binding (see Figure S2A–D), thereby making this popular ratiometric pH indicator a complex acceptor in a FRET-based nanobiosensor. In the present work, we addressed this problem by introducing a new custom red-emitting pH indicator, HRuby [14] derived from our successful family of Calcium Ruby red-emitting Ca^2+^ indicators [15,16,17,18]. This new pH indicator displays no spectral shift upon proton binding. The absorption of HRuby overlaps with the emission of the CANdot@565 (QD) and its emission is well separated from the donor. 

We here report the synthesis, characterization and validation of a QD/Ca (H)Ruby-based FRET pair and demonstrate that the bound Ca(H)Ruby titrates against Ca^2+^/protons as the free dye in solution. Finally, we validate both *in vitro* and *in situ*, a FRET-based cell penetrating Ca^2+^ nanobiosensor as well as a H^+^ nanobiosensor engulfed in the endocytotic pathway.

### 1.1. Material and Methods

Chemistry of CaRuby1 compounds and HRubies will be found in [17] and [19] respectively. Chemistry of CaRuby2 is described in the Supplementary Information and documented with Scheme S1 for the synthetic strategy and Figures S1–S46 for the characterization of the synthesis intermediates and the final compounds.

Most of the protocols used here (for QDs surface chemistry, conditions for maintaining the BHK cell line stably expressing NR2A-NMDAR and their use for TIRF microscopy) have been previously described in the Supporting Information of Zamaleeva *et al.*, 2014 [8] to which one will refer for the synthesis of peptide-coated QDs, functionalization of QDs and their purification, cell culture of the BHK cell line expressing NR2-NMDAR, and the in cell imaging of single particles using TIRF microscopy. We mainly describe here the protocols relevant to the study of HRu-PiAC-based pH nanosensors.

### 1.2. Dyes PEGylation

CaRubies PEGylation using the side chain for click chemistry has been detailed by [8]. The same PEGylation procedure was followed for HRu-PiAC.

### 1.3. Fluorimetry

Methods used for CaRubies2 have been published previously [18]. Briefly, CaRuby dynamic range for Ca^2+^ sensing was estimated from peak PL of CaRuby measured in a solution containing (in mM) 100 KCl, 30 MOPS, where [Ca^2+^] was adjusted using the Invitrogen Ca Buffer kit (Life Technologies, ref: C-3008MP). For HR-PiAC titration we used the universal pH buffer, see Supplementary 3, p. 830 in [20] which has an almost constant ionic force over the 2 to 12 pH range. Fluorescence curves are corrected for the pH sensitivity of the QDs fluorescence (see Figure S3 for details). FRET fluorescence spectra (500–700 nm) were obtained by excitation light at 407 nm, and direct emission spectra (550–700 nm) were obtained by excitation at 545 nm. All values for FRET pairs were calculated, after spectral linear unmixing, by fit to the QD and Ca/HRuby spectra (MatLab curve fitting tool). 

Confocal Microscopy: Plated cells were incubated with the pH nanosensors (at 100 nM QDs in DMEM nutrient medium without serum) for 2 h, and then for 30 min with 500 nM Lysotracker Green. After they were washed twice with phosphate-buffered saline (PBS) alone (Invitrogen, Cergy Pontoise, France), finally a HEPES buffered DMEM medium was reintroduced. Live cells were then immediately analyzed by confocal laser scanning microscopy using a Zeiss LSM operating system. pH probes (561 nm) and Lysotracker green (488 nm) were simultaneously excited and emission fluorescence was collected.

### 1.4. Intracellular Calibration of pH Sensors by Flow Cytometry

The intracellular calibration of the pH sensors was performed using BHK-21 cell line. Plated 80% confluent BHK cells were incubated with 100 nM QDs for 1 h in the medium without antibiotics or fetal calf serum, and then the cells were allowed to rest in an incubator (5% CO_2_, 37 °C). After 2 h, the cells were harvested using TrypLE^TM^ Express Enzyme (Gibco, Waltham, MA, USA) and resuspended in a CO_2_-independent medium (Gibco, UK).

The K^+^/H^+^ ionophore nigericin was used for intracellular calibration of pH sensors. Together with a high concentration of potassium in the buffer, it equalizes the intracellular and extracellular pH. The cells containing internalized pH sensors were resuspended in buffers containing 143 mM KCl, 1.17 mM MgCl_2_, 1.3 mM CaCl_2_, 5 mM glucose and 10 µM nigericin with defined pH buffers ranging from pH 4.0 to pH 8.0 with 0.5 increments. Then, 20 mM citric acid was used for a buffer range of pH 4.0–6.5 and 20 mM (4-(2-hydroxyethyl)-1-piperazineethanesulfonic acid) (HEPES) for buffers in the pH range 7.0–8.0. DAPI (4′,6-diamidino-2-phenylindole; 0.05 µM) was added to exclude the dead cells. After incubation for 5 min at room temperature the samples were analyzed by a flow cytometer (BD LSR Fortessa, BD Biosciences). For acquisition, a 532 nm or 405 nm wavelength lasers were used for H-Ruby direct or FRET excitation, respectively, and its emission fluorescence was collected in a 610 ± 20 nm spectral detection channel. Each time 10,000 events were acquired. A FlowJo vX.0.7 software was used for the analysis. First gating on the cells by forward/side scatter was done, then a population of live cells was selected and a mean fluorescence intensity of H-Ruby for each sample was determined. 

## 2. Results

### 2.1. From the Principle to a Modular Toolkit: Construction of FRET-Based Ion Sensors

#### 2.1.1. Principle

**Figure 1:**
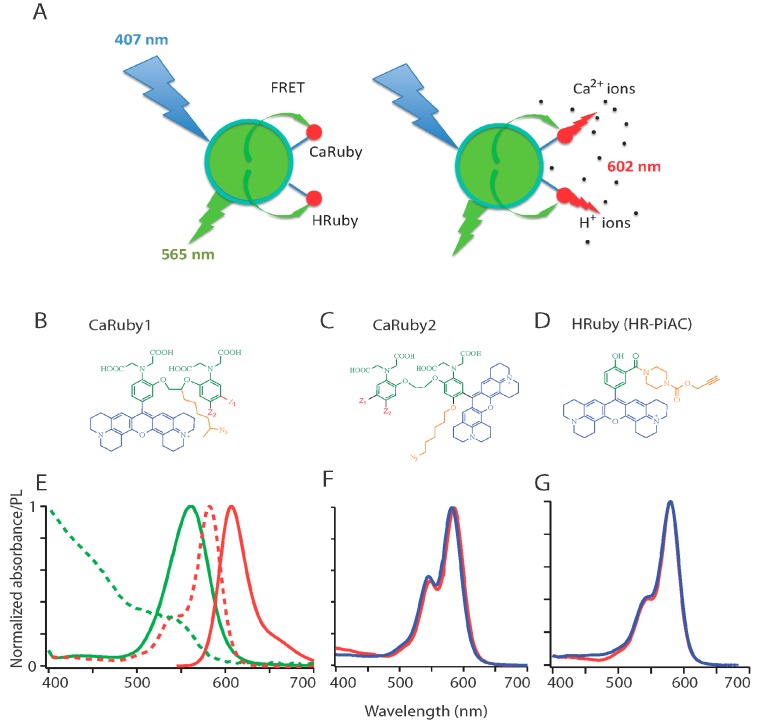
FRET-based red-emitting ion sensors: (**A**) Principle. Coupling a green-emitting quantum dot (QD: here CANdot^®^565) donor to a red-emitting rhodamine-based ion sensor, the acceptor, produces an analyte-dependent FRET signal upon donor excitation at 405 nm. The custom red-emitting ion sensors used here are Ca^2+^ (alternatively H^+^) indicators, the emission of which is quenched by PET in absence of their ligand. Analyte binding results in a strong fluorescence peaking at 602 nm; (**B**–**D**) Chemical structure of the sensors: All sensors are built on an extended rhodamine moiety (blue). The two Ca^2+^ sensor families incorporate a BAPTA moiety (green), without (**B**) and with (**C**) an oxygen introduced on one of the aromatic ring of the BAPTA for the lower and higher affinity families: CaRuby1 (µM-mM range) and CaRuby2 (sub-µM range), respectively. Substitutions (Z_1_, Z_2_ in red with Z_1_=Cl, Z_2_=H for the chloride derivatives, and Z_1_=H, Z_2_=F for the fluorine derivatives, Z_1_=H, Z_2_=Me for CaRu1-Me) yield compounds with a finely tunable K_D_ for Ca^2+^ binding. The pH sensor family (HRubies, (**D**)) is based on the addition of a phenol instead of a BAPTA. Note that all compounds bear an azido/alkyne side arm for click chemistry and the resulting potential for high-yield coupling reactions. The azide bearing linker is introduced in the bridge between the two aromatic rings of the BAPTA for the CaRubies1, and on the additional oxygen for the CaRubies2. HR-PiAC bears an alkyne moiety at the ortho position of the phenol through a piperazine carbamate link; (**E**–**G**) Spectral properties of retained donor/acceptor pairs (**E**) normalized absorbance and emission spectra (dashed and plain lines, respectively) of QD565 (green) and CaRu-Me (red). Since there is only a slight Ca^2+^ sensitivity of CaRu-Me absorbance when switching from EGTA- to 2 mM Ca^2+^-containing solution, the K_D_s of CaRu-Me and QDCaRu-Me were similar, as expected. Similar properties are expected with CaRu2-F and HR-PiAC since their absorbance is respectively Ca^2+^ and pH insensitive, as illustrated in panels (**F**) and (**G**), where blue and red traces are in absence and presence of the analyte, respectively.

We constructed FRET-based nanobiosensors from a central QD donor (D) and 1 to 10 ion-sensitive acceptor (A) dye molecules (Figure 1A). For the latter, we used members of our custom family of Ca^2+^ and H^+^ Ruby sensors having absorption and emission spectra matching that of a green-emitting commercially available QD donor, CANdot^®^565 and leading to a calculated Förster radius of about 4.5 nm (assuming dipole–dipole coupling, and isotropic orientation) thereby indicating an efficient FRET. The fluorescent acceptor combines an extended rhodamine fluorophore, an ion specific-chelating group and a linker arm allowing its conjugation to the QD surface (Figure 1B–D).

In the Calcium Ruby probes, while the fluorescence quantum yield Φ_F_ increases upon Ca^2+^ binding, its absorbance remains unchanged so that the FRET between donor and acceptor is independent of analyte binding and merely relays excitation, leading to an apparent large Stokes’ shift of 40 nm when excited in the blue (at 405–407 nm). However, Φ_F_ changes from a quenched state at low analyte concentration, to a bright deep-red sensitized emission at higher analyte concentration. The FRET efficiency increases with increasing A:D ratio, as expected for multi-acceptor FRET that offers multiple non-radiant deexcitation pathways and acceptor orientations for the QD donor. As a result, at low acceptor concentrations, the nanobiosensor can be located upon blue excitation and detection of green/yellow QD emission, and the analyte concentration be monitored above 600 nm either by FRET or by direct excitation of the Rubies near 560 nm. For a multi-acceptor system (with a A/D >5), the nanoparticle is merely a carrier to up-concentrate the ion sensor, and the donor fluorescence is so much quenched that, even at resting low ion concentration, the ensemble can only be located by the red emission.

#### 2.1.2. Ion Sensor Design

We recently introduced a family of functionalizable red-emitting ion indicators based on an extended (X-) rhodamine, incorporating either a BAPTA-based Ca^2+^-sensing moiety (European patent, EP 13 194 728.5) or else a phenol-based moiety for H^+^ sensing (EP 13 199 575.5). In these compounds (Figure 1B–D), ion binding to the nitrogen of BAPTA (for the Ca^2+^ probes, [21]) or to the phenolate (for the pH probe) blocks the photoelectron transfer (PET) responsible for fluorescence quenching at low ion concentration. Therefore, ion binding leads to an unquenched fluorophore at high analyte concentration. To allow fluorimetry upon single-wavelength excitation as well as two-photon imaging, we only retained compounds having no absorption shift when the ion concentration is varied (Figure 1F,G). We can at this stage expect the same calibration curve for the free sensor and on the functionalized QD, unless non-trivial phenomena introduce more complicated effects.

#### 2.1.3. Ca^2+^ Sensors

All Calcium Rubies (CaRuby) bear (orange, in Figure 1B,C) an azido spacer arm for click chemistry and the resulting potential for high-yield coupling reactions [22]. This side arm efficiently allows conjugation reactions without using the carboxylic groups of the BAPTA moiety [23] and conjugation therefore introduces no significant perturbation of the Ca^2+^ binding affinity [8].

A first-generation of CaRubies (from now on referred to as CaRuby1) was constructed having the side arm attached to the ethylene glycol bridge between the two aromatic rings of the BAPTA [15,16,17]. These CaRubies featured dissociation constants from 3.4 to 21.6 µM, depending on the halide substitutions on the aromatic rings (Z_1_, Z_2_ in Figure 1B,C). To increase further the Ca^2+^-binding affinity of CaRuby, we introduced an oxygen atom on one of the aromatic rings of the BAPTA by a S_N_Ar reaction which also serves as a link for the azido side arm in the second generation of new CaRuby variants, CaRuby2 ([18] and Figure 1C). Additionally, in the latter case, the fluorophore was placed in a meta position so as to reduce its effect on the chelating nitrogen. These modifications resulted in variants with sub-micromolar affinities for Ca^2+^ binding and ranging from 0.26 µM for CaRuby-Nano [18], now dubbed CaRuby2-H (see Figure 1B,C), 0.325 µM for CaRuby2-F and 1.71 µM for CaRuby2-Cl leading to an overall range of 0.26 to 22 µM covered between all CaRubies. 

The absorption and emission spectra of CaRuby2-F is identical to that of Caruby1-CH_3_ shown in Figure 1E where the red dotted line shows the QD emission spectrum. The dynamic range and QY of the yet unpublished CaRubies2 are given below in Table 1 (see [17,18]) for CaRubies1 and CaRuby2-H, respectively). Importantly for physiological applications, these sensors displayed no sensitivity to Mg^2+^, and were poorly pH-dependent (Figure S1C–E; see also [17,21] for CaRubies1 and [16,18] for CaRuby2-H). On the other hand, these compounds are quenched by Cu^2+^ ions that hence can be applied to quench extracellular dye molecules or perform calibration experiments.

**Table 1:** CaRu_2_-Cl and CaRu_2_-F biophysical characteristics.

CaRu_2_-Cl	**State**	**λ_abs, max_**	**λ_em, max_**	**K_D_**	**ε (M^−1^·cm^−1^)**	**Ф**	**Dynamic**
		**(nm)**	**(nm)**				**Range**
	ON ^a^	583	606	1.71 µM	70,120	0.63	33
	OFF ^b^	580	605		76,550	0.02	
CaRu_2_-F	ON ^a^	583	608	0.33 µM	89,190	0.60	63
	OFF ^b^	580	606		90,240	0.01	

^a^ 2 mM Ca^2+^; ^b^ 10 mM EDTA.

#### 2.1.4. H^+^ Sensors

By following the same strategy, a series of single-wavelength excitation red-emitting pH indicators were synthetized [19]. These HRubies are equally X-Rhodamine-based with a phenol moiety engaged in a PET in absence of protons. The side arm for click conjugation is here attached to a carboxylate introduced in ortho position on the phenol. Among the various HRubies, the compound HRuby-PiAC shares the same spectral properties as the CaRubies (not shown), allowing its use in EGFP-expressing cells without appreciable crosstalk [16] and it has an adequate pK_a_ for physiological experiments (pK_a_ = 7.68 ± 0.09). A fluorescence quantum yield of Ф_F_ = 0.64 at pH 4 as well as a large dynamic range of 86-fold makes it a promising tool for studying cytosolic pH gradients and signaling events. Of note, this new pH sensor has no sensitivity to divalent cations (Figure S2E,F).

#### 2.1.5. Quantum Dots (QDs)

The absorption spectrum of all Ruby indicators overlaps with the emission spectrum of CANdots^®^ 565 (CANdots, Hamburg, Germany; λ_em_= 565 nm). After several attempts with different commercial and custom nanoparticles, we opted for these CdSe core/CdS-ZnS shell-type QDs that proved to be very stable, the hexane of a single initial stock solution being substituted for toluene when needed. While these non-functionalized peptides-coated QDs displayed no change in absorbance when changing from low to high ambient Ca^2+^ concentrations, their emission displayed a sensitivity to pH (Figure S3), which was corrected for in presented data by taking a linear interpolation in the range of pH values between 4 and 12, corresponding to an effective three-fold increase in fluorescence.

#### 2.1.6. QDs Surface Chemistry and Functionalization Reaction

**Figure 2:**
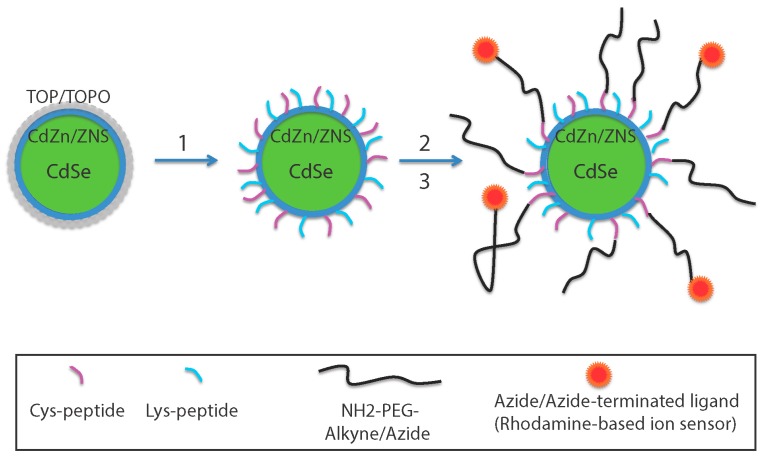
Assembly of FRET-based ion sensors. CANdots have a CdSe core surrounded by a double layer of CdZn and ZnS. The QD TOP/TOPO (Step 1) passivating layer was replaced by a hydrophilic coating peptide made by mixing 50% cysteine-(SH function) and 50% lysine-(NH_2_ function) terminated peptides (Cys-peptide: Ac-CGSESGGSESG(FCC)_3_F-amide and Lys-peptide: NH_2_-KGSESGGSESG(FCC)_3_F-amide, respectively). Independently, an azide/alkyne-terminated ligand was bound to a clickable NH_2_-PEG to form a PEGylated dye (Step 2). Nanoparticles were then functionalized by adding the pegylated rhodamine-based sensor (red dots) (Step 3) using a SH/NH_2_ crosslinking reaction. Other NH_2_ terminated ligands can be added using the same crosslinking reaction and are included in stoichiometric ratio (not shown).

To have a controlled number of the biomolecules linked to a QD, a simple, yet effective approach is to use the same reaction to introduce all reactants and have them present at the desired final stoichiometry. The retained protocol (Figure 2) aimed at obtaining hydrophilic QDs by coating them with peptides bearing a terminal thiol group (SH) for a SH/maleimide linking reaction to functionalize the QDs [24]. For this purpose, as described previously [8], the passivating trioctylphosphine/trioctylphosphine oxide (TOP/TOPO) layer was substituted by some 25–30 short phyto-chelatin-related peptides anchored by metal-affinity to the CdZnSe QD surface, among which maximally 85% of them (to preserve the high QY) carried a SH function, other coating peptides being NH_2_-terminated. This coating peptide retains the photophysical properties of the original QDs with a final fluorescence quantum yield of Ф_F_ = 56% of the fluorescent donor. 

#### 2.1.7. Construction of the Nanosensors

To functionalize the QDs, the various ligands were prepared as maleimides for direct binding onto the surface SHs. 

To link Ca/HRuby, a NH_2_-terminated–PEG-Ruby was prepared (Step 2 in Figure 2). A commercially available NH_2_-PEG-alkyne/azide was linked to CaRuby-N_3_ or HRuby-alkyne by click chemistry. Finally, this compound after a N-γ-maleimido-butyryloxy-succinimide ester (GMBS) crosslinking reaction was linked to the QDs as a maleimide-PEG-Ca/HRuby.

Similarly, the cell-penetrating peptides (CCPs) to be added were prepared with a maleimide function.

## 3. Biophysical Properties of the Nanosensors

### 3.1. Spectral Properties

**Figure 3:**
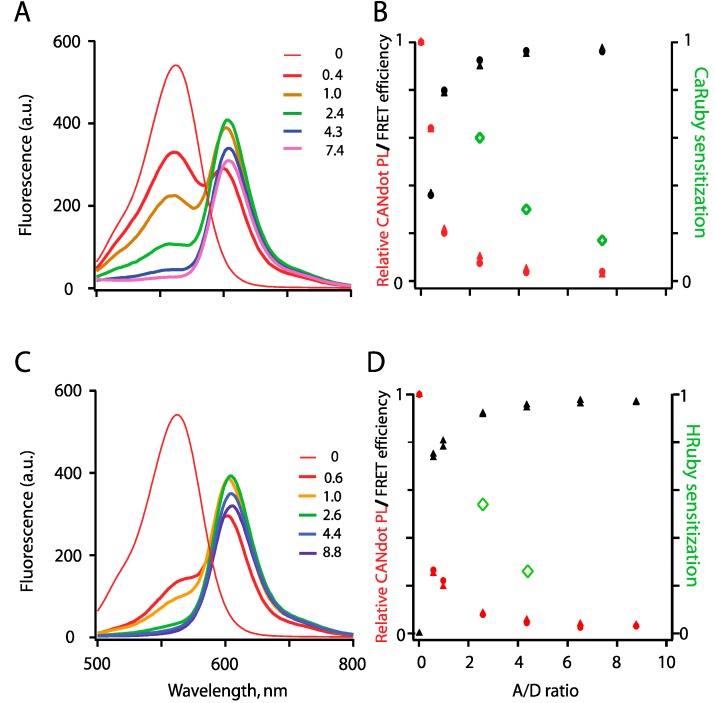
FRET between a QD565 donor and rhodamine-based ion-sensing acceptor fluorophores. FRET was measured as donor quenching upon QD excitation at 407 nm and acceptor sensitization, as a function of A/D ratio. Experiments were performed at an elevated analyte concentration (500 µM) to fully relieve the PET quenching of the dye. (**A**) Series of mixed spectra obtained by increasing the number of PEG5kDa -CaRuby2-F while keeping QD concentration constant (40 nM), in saturating Ca^2+^, 500 µM. The A/D ratio was determined by absorbance measurements at 407 and 581 nm, to evaluate QD565 and CaRuby concentrations, respectively; (**B**) Relative donor quenching (QD photoluminescence, red) and FRET efficiency (black) are reported after linear unmixing of donor and acceptor spectra, see Supplementary Material 6. Green data points shows acceptor sensitization (F_CaRuby_ (exc. @ 350 nm) − F_QD_ (exc. @ 350 nm)/(F_CaRuby_ (exc. @ 535 nm)); (**C**,**D**) Similar experiments were carried with QD-HR-PiAC, with the pH adjusted to 4. In both cases, symbols ▲, ● show results from two independent runs.

Figure 3 illustrates the emission spectra of the QD-Ruby complexes at high analyte concentration where FRET from the QD to the rhodamine moiety is revealed by the detectable acceptor fluorescence. Similar to what we reported earlier for CaRuby1-Me [8], the CANdots565^®^/CaRuby2-F-pair produced an efficient FRET with an increase in efficiency as the number of bound CaRuby molecules on the QD surface increased, as expected for multi-acceptor FRET (Figure 3A,B). Similarly, FRET was observed with HRuby (Figure 3C,D). FRET characteristics of both the Ca^2+^ and H^+^ nanobiosensors are illustrated in Figure 3B,D, respectively. Noteworthy, maximal FRET efficiency was attained with distinct A/D ratio depending on the dye bound to the QD. It is not maximal with QD-PEG5-CaRu1-Me although using an A/D ratio of 18 [8]. With CaRu2-F and HRuPiAC as the acceptors, peak value is already attained with only three dye molecules inserted in the otherwise same QD-PEG5kDa-Rubies constructs. Obviously in these two latter cases, a slight acceptor quenching due to a not yet identified mechanism occurs at higher A/D ratio, whereas the CANdots photoluminescence regularly decreases (note the very sharp decrease with HRuby).

Measured mixed donor and acceptor emission spectra (shown in Figure 3A,C) were linearly unmixed using standard procedures (MATLAB) to calculate the relative donor luminosity and FRET efficiency. The later (E = 1 − F'_D_/F_D_, where F’_D_ and F_D_ are the donor fluorescence intensities in the presence and absence of the acceptor, respectively) increased up to 80% while acceptor sensitization plateaued at 20% when the number of PEG5kDa-CaRuby was increased to 10.

### 3.2. Ion Sensing

As previously with CaRu1-Me, we systematically investigated whether the new second-generation CaRubies retained their ion sensitivity once they were coupled to the QD surface. We characterized the Ca^2+^-sensitivity of the assembled FRET pairs with fluorimetric titrations in which we switched from a nominally Ca^2+^-free solution (zero Ca^2+^ and 10 mM EGTA: peak photoluminescence, PL = F_0_) to saturating ([Ca^2+^] = 2 mM for CaRubies1 and 6 µM for CaRubies2; peak PL = F) and we used the relative increase in CaRuby fluorescence (dynamic range DR = (F − F_0_)/F_0_) as an index of sensitivity. For H sensors, we proceeded analogously by switching from basic conditions (pH = 10) to an acidic solution at pH 4 and again we used DR as an index of sensitivity.

**Figure 4:**
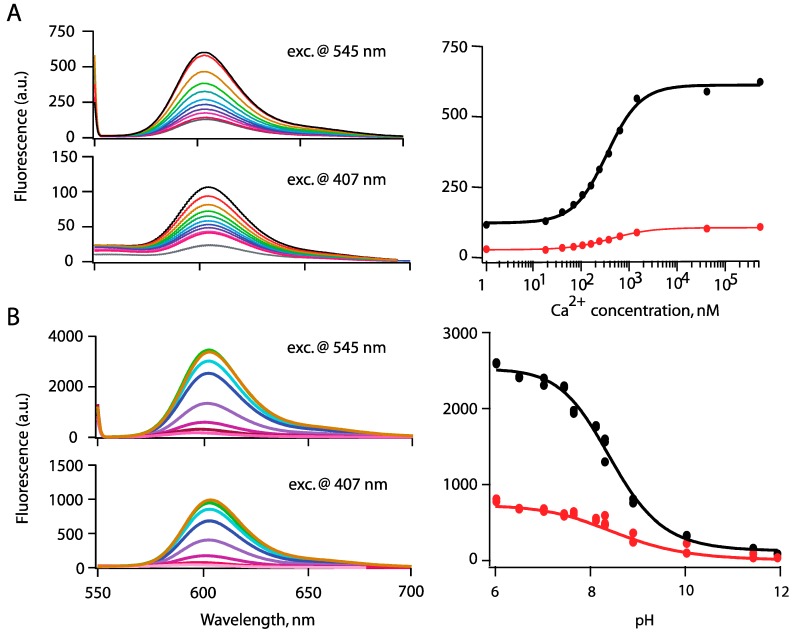
(**A**) Fluorometric titration of FRET-based nanobiosensor assemblies: QD-PEG5kDa-CaRu2F (prepared with A/D ratio= 7.4, and using the Invitrogen Ca^2+^ buffer kit to adjust [Ca^2+^]); Spectra (**left**) were obtained when following Ca^2+^ concentrations were successively applied: 1, 17, 38, 65, 100, 150, 225, 351, 602 nM and 1.35 and 39 µM from bottom to top traces; (**right**) Resulting titration curves using direct excitation at 545 nm or FRET excitation upon QDs excitation at 407-nm; (**B**) QD-PEG5kDa -HR-PiAC (A/D ratio= 5.6), universal pH buffer, see Supplementary 3, p. 830 in [20]). Fluorescence curves are corrected for the pH sensitivity of the QDs fluorescence (see Figure S3 for details). Spectra shown on the left correspond to pH 6, 7.45, 7.65, 8.3, 8.9, 10.3, 11.45 and 11.9 from top to bottom, respectively. On the right: measured *K*_D_s are similar when carrying fluorometric titration either by direct excitation (at 545 nm) or by FRET excitation (at 407 nm).

The resulting probes contrasted sharply for Ca^2+^ *vs.* H^+^ sensors, respectively. Although CaRubies retained a high Ca^2+^-sensitivity after binding to PEG by click chemistry, QD-CaRuby pairs built from shorter PEGs were unexpectedly largely Ca^2+^-insensitive. This observation initially made with PEG0.3kDa -CaRu1-Me ((DR = 0.5, n = 2; [8,17]) also holds true for CaRu2-F combined to PEG3kDa (data not shown). However, with PEG5kDa, an A/D ratio in the range of 5–15 produces systematically a Ca^2+^-dependent fluorescence large enough to allow efficient Ca^2+^-sensing (as illustrated in Figure 4A where A/D = 7.4). As we aimed at visualizing and hence localizing nanobiosensors inside cells, even at resting intracellular [Ca^2+^], we finally retained an A/D ratio of 5–10 as a compromise for *in situ* biological Ca^2+^ imaging. Since nanobiosensor performance is dominated by the characteristics of the acceptor BAPTA, which has a diffusion limited k_on_(Ca^2+^) (see [8] for CaRuby1-based constructs), the very fast time response of all CaRuby-based constructs will allow to measure fast Ca^2+^ transients. Of practical importance, once assembled these nanobiosensors proved to be stable over at least two months. Thus, taken together, a central 565CANdot with a low number of CaRuby acceptors emerges as a promising tool for Ca^2+^ nanobiosensing. 

The situation is different with HRuby-PiAC. Whereas the DR was reduced to about half of that of the free indicator in solution upon PEG binding, a DR of 30 was maintained upon PEG-HRuby binding to the QD surface (Figure 4B, here the A/D ratio was 5.6). 

The origin of the sensitivity of the CaRuby to its environment is not yet understood, but since once linked to a third component the rhodamine quenching by BAPTA was reduced, we speculate that the crosslinking reaction led to a reduced mobility of the two molecules involved.

Next, we examined the analyte binding affinity of Rubies once linked to QDs. As expected, the K_D_s obtained by fluorescence titration were almost identical between the dye alone and the QD-dye conjugates. With direct excitation, the more sensitive condition, the K_D_ measured with QD-CaRuby2-F was 391 ± 2 nM and with QD-HR-PiAC pH 8.25 ± 0.25 compared to 325 ± 50 nM and pH 7.7 ± 0.1 for the isolated dyes (see Figure 4, as well as Figures S1 and S2). K_D_(H^+^) value for QD-HRu-PiAC was similar when measured upon direct or FRET excitation of the dyes (8.25 ± 0.06 and 8.4 ± 0.1 respectively), and only slightly shifted towards lower affinity as compared to the free dye (K_D_ = 7.8 ± 1). The situation is different with QD-CaRu2 where FRET excitation yields a notably higher K_D_(Ca^2+^) than the one obtained by direct excitation, with a shift up to 1.3 ± 0.3 µM (as compared to 391 ± 2 nM). Such pKa difference may originate from the local environment of the probes and from their distance to the center of the nanocrystals: FRET emission excites preferably the closest probes prone to surface charge sensitivity—and the negative environment found there reduces the acidity of the probe—whereas direct excitation give a general and an average overview of the protonation status of the probes.

## 4. Cell Imaging

**Figure 5:**
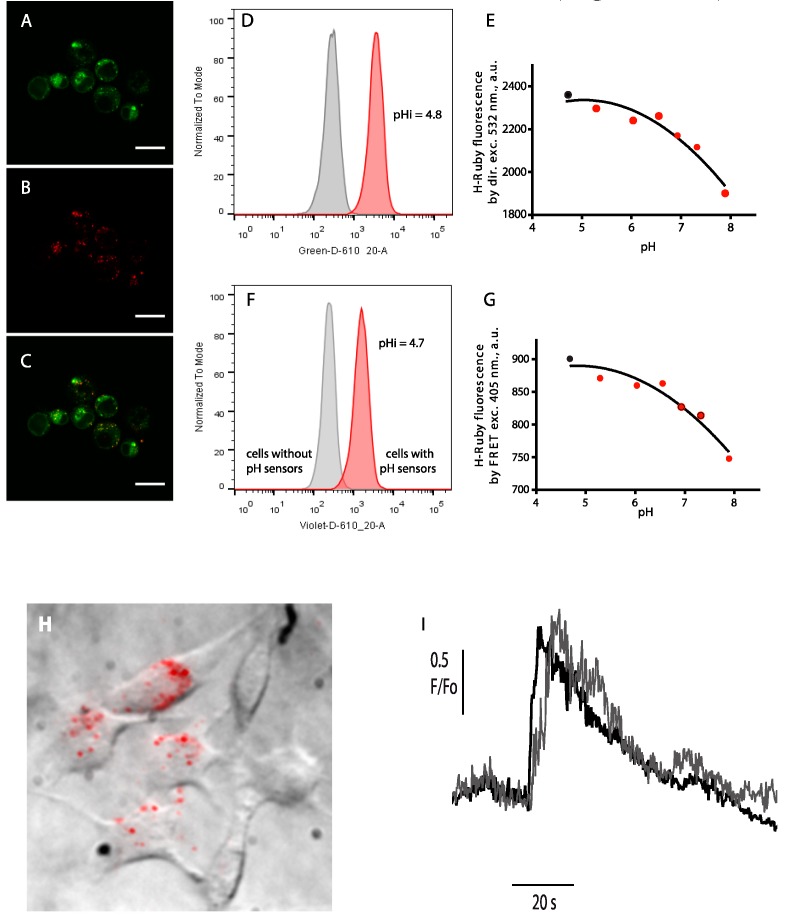
Live-cell imaging of intracellular ion distributions. (**A**–**C**) Confocal images of BHK-21 cells after incubation with QD-HRu PiAC (**A**) co-stained by Lysotracker Green; (**B**) and a merged image of the two channels (**C**). Scale bar is 20 µm; (**D**–**G**) Intracellular calibration of pH sensors in a suspension of BHK-21 cells using flow cytometry. Typical histograms showing the internalization of the QD565/H-Ruby pH sensors according to the H-Ruby intensity (in grey, the cell without sensors, and in red, the cells having internalized pH sensors) upon direct (**D**) and by FRET excitation (**F**). Calibration curves of the intracellular pH sensors clamped at different pH with the ionophore nigericin were obtained by direct (**E**) and by FRET excitation (**G**) of H-Ruby. Intracellular pH as measured by the red histograms of fluorescence H-Ruby in (**D**,**F**) was evaluated (black dots) by extrapolation on the calibration curves (**E**,**G**), respectively; (**H**,**I**) Read-out of local Ca^2+^ transients upon glutamate application on BHK cells stably expressing the NR_1_ and NR_2_A subunits of the N-methyl-D-aspartate receptors (NMDARs). (**H**) Firstly internalized biosensors (QD-CaRuby-CPP in a ratio 1:10:10) were localized by superimposition of the bright-field and time-averaged TIRF image of cultured BHK cells detected in the green channel upon 405-nm evanescent-wave excitation of the QDs. (**I**) Repetitive stimulation evokes reversible Ca^2+^ transients. Superimposed traces obtained in the red channel following 568-nm excitation are responses of the Ca^2+^ sensor to two successive bath applications of NMDAR agonists at a saturating concentration, with a 15 min long continuous bath perfusion of control saline for recovery from desensitization in between. Figure 5H,I is reprinted with permission from Zamaleeva *et al.*, 2014 [8]. Copyright 2015 American Chemical Society.

Finally, we validated our H^+^ and Ca^2+^ nanobiosensors for the detection of local, ionic signals in live cells. We first showed the use of QD-HRuby to detect an induced cellular pH change and to reveal the acidic endosomal content (Figure 5A–G), and, secondly, we demonstrate that QD-CaRuby2-Me loaded in the cytoplasm are able to follow intracellular Ca^2+^ transients (Figure 5H,I).

### 4.1. Internalization of HRuby-Nanosbiosensors along the Endo-/Lysosomal Route

Free QDs non-functionalized by (bio-)macromolecules have the possibility to enter cells by different endocytotic pathways. Depending on the nature of their surface coating, QDs internalization can occur via lipid raft/caveolae endocytosis [25], via phagocytosis [26] or micropinocytosis [27]. In either case, internalization is followed by the accumulation of the nanoparticles in endo-lysosomal compartments. After a 2 h cell exposure to QDs-HRuby, we show these constructs to be localized in the lysosomal compartment (Figure 5A–C). The acidic environment of the endo-/lysosomes together with opsonisation by endocytic-pathway proteins can induce an aggregation of the QD-based sensors and affect their sensitivity [19]. To investigate this issue, we incubated QD-H-Ruby with BHK fibroblasts in a serum free medium for 1 h and let the cells rest for two more hours. The cells were then harvested and analyzed by flow cytometry where the fluorescence intensity of intracellular QD-H-Ruby was collected both upon the direct excitation of H-Ruby and upon FRET excitation. As shown in Figure 5D,F, both imaging modalities could be used for detection and read-out of intracellular pH sensors. However, the direct excitation of H-Ruby gives an almost three times stronger signal compared to FRET excitation, which is consistent with the earlier spectrofluorimetric characterization of QD-H-Ruby pairs (Figure 4). Next, in order to determine the pH of QDs localized organelles we prepared the standard samples of the cells by bringing intracellular pH to external defined pH using the K^+^/H^+^ ionophore nigericin. Extrapolation of the fluorescence intensity of H-Ruby of the samples shown in the Figure 5E,G from a calibration curve of the standard samples (Figure 5D,F) reveals an acidic intra-organelle pH in the range of 4.7 to 4.8 that corresponds to lysosomes as expected. 

### 4.2. Cytoplasmic Ca^2+^ Measurements

In a second set of experiments aiming at detecting intracellular Ca^2+^ transients, we favored a direct cytoplasmic delivery of our nanobiosensors by further linking the QDs to cell-penetrating peptides (CPPs) (see [8] for a discussion of the exact routes of entry). As a CPP, we selected H11, a very high-affinity peptide derived from hadrucalcin, a scorpion toxin directed against the intracellular ryanodine receptor [28,29]. This CPP was synthetized as a maleimide derivative (Smartox Biotechnology, Saint Martin d'Hères, France) and stoichiometrically combined with CaRuby to QDs. We loaded for one hr QD-CaRuby1-Me-H11 nanobiosensors into BHK cells stably expressing the NR_1_ and NR_2_ subunits of the Ca^2+^-permeant NMDA receptor (NMDAR), then trypsinized the incubated cells to replate them directly on a quartz slide and let them settle down and attach overnight [30]. We first visualized cells contours in bright field and then selected cells having incorporated near-membrane QD sensors using total internal reflection fluorescence (TIRF) microscopy. Donor and acceptor fluorescence were simultaneously detected upon 405- and 568-nm evanescent-wave excitation on a custom prism-type VA TIRF microscope (see Methods in Supplementary Information, and [8]). Evanescent-wave illumination allowed visualizing only those nanosensors close to the basal cell membrane with a high contrast, permitting the readout of the fluorescence emitted by individual QD-sensors and small aggregates (Figure 5H, here underlined as red dots). Ca^2+^ transients were evoked by NMDAR activation (bath application of 100 µM glutamate, 20 µM glycine and 5 mM CaCl_2_) and responses were continuously monitored at 4 Hz during 3 min before and during agonist application. Figure 5I shows that Ca^2+^ transients differed at different subcellular locations, but were comparable for each site upon successive applications of NMDAR agonists. 

## 5. Discussion and Conclusions

### 5.1. Towards a Working in Cellulo Proton-Nanobiosensor

Based on the previous development of a FRET-based Ca^2+^ sensitive nanosensor, we now present a conceptually similar FRET-based pH-sensitive nanobiosensor. Both cation sensitive dyes used for this purpose are constructed on extended rhodamine scaffolds that are quenched by the Ca^2+^/H^+^-binding moiety, which itself is dequenched upon analyte binding, and in turn relieves the fluorescence quenching. This PET is relieved when a BAPTA motif binds Ca^2+^ in the former case, or when a phenol ring is exposed to an acidic medium in the latter case. The QD surface chemistry already developed [8] to construct such sensors was extended reliably with no further modifications to the whole family of these fluorescent cation indicators. We have previously extensively described and discussed the properties of the CaRuby-based sensor [8]. Therefore, here we will mainly focus on the properties of the HRuby-based sensor, more precisely on a HRuby-PiAC probe. HRubies have a poor sensitivity to divalent cations that make them promising tools for physiological studies. Once bound to QDs through a hydrophilic PEG chain, the pK_a_ of HR-PiAC was only slightly displaced to higher values, and, most interestingly, it kept its wide dynamic range (~30) with maximal fluorescence at pH 4. These observations provide guidelines for the design of a further improved dye for in cell studies. Of course, any modification reducing the high lipophilicity of the rhodamine will be a plus and a slightly more acidic K_d_ would allow monitoring pH changes in intracellular compartments. By these constructs, we extend the toolbox of smart sensors allowing *in cellulo* pH mapping [14,24].

### 5.2. What is the Reason for the Loss of Dynamic Range for Ca^2+^ Nanobiosensing?

**Scheme 1:**
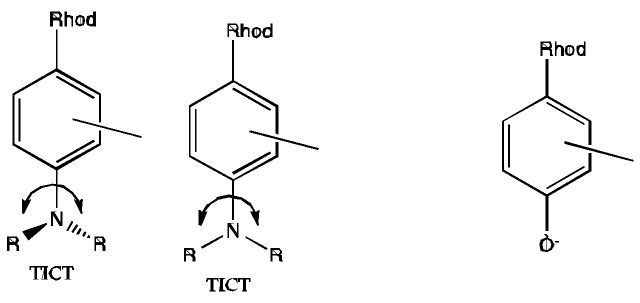
Twisted-intramolecular charge transfer in CaRuby (at the **left**) and HRuby (at the **right**) dyes.

Despite the similar behavior of the CaRubies and HRubies once bound to a PEG and further to a QD, the poor dynamic range for Ca^2+^ detection as opposed to the maintained good dynamic range of the pH sensors is the most intriguing finding in this study. This effect could probably be attributed to a change in local viscosity around the Qdot shell. In the case of Ca^2+^ detection, the efficacy of the PET quenching in the CaRuby relies on an efficient TICT (twisted-intramolecular charge transfer) effect, promoted by a fast rotation around the C-N bound leading to a geometrically favorable state (Scheme 1). In viscous environment, the mobility of the rotor is reduced and the quenching is less efficient [31,32]. 

For such molecular rotors, the Forster and Hoffman equation links quantum yields (Ф_F_) and viscosity (η): log Ф_F_ = C + x log η. This effect was well studied in a closely related 4-dimethylamino rhodamine by [33] using glycerol solution. Quantum yields increased with viscosity and reached a plateau of 40% in very viscous solution. In contrast, a similar effect is not expected with H probe, as no group movement is required for its quenching.

### 5.3. pH Sensitivity of QD Luminescence

A contaminating sensitivity of the QD luminescence itself to the analyte would bias measurements. The CdZn/Se QDs that we used here do not display a substantial sensitivity to Ca^2+^ but their sensitivity to pH incited us to introduce a corrective factor in our pH estimates. A shifted pK (8.25 instead of 7.7) of the QD-bound compared to the free indicator was maintained, even after this correction. 

### 5.4. An Effect of Surface Charge?

An accurate reading of ion concentrations would also be made difficult by a surface charge effect, leading to unknown local analyte concentrations near the QD surface as previously suggested [34]. This interpretation has been made in the case of the inclusion of the ion-sensitive indicator (Ca^2+^ and also Cl^−^) in a colloidal polymer [35,36,37,38] to explain the altered pKa of the indicator once integrated in the polymer. Our present results indicate that the negative charges of the QD surface do not interfere much with the Kd of a CaRuby when bound to the QD via a PEG-linker, and that they only slightly modified (by 0.4 pH unit) the pKa of HRuby. Our interpretation in this case is that the observed shifts, if any, are induced by an altered PET between the ion-sensing radical and the rhodamine, a process which is intrinsic to the construction of the CaRuby. This issue may possibly be optimized in the future. A case where inter-molecule quenching occurs would be the formation of *H*-aggregates between two fluorescent acceptor molecules linked to the same QD. In view of the small number of dye molecules bound to a single QD, this process is, however, unlikely to occur in our constructs. 

## Supplementary Files

Supplementary File 1
