# Supplementary material for: FRET-Based Nanobiosensors for Imaging Intracellular Ca2+ and H+ Microdomains
Source: Sensors (Basel). 2015 Sep 23;15(9):24662–80. doi: 10.3390/s150924662 (PMC4610457; doi:10.3390/s150924662)
Supplement: Supplementary File 1 [file sensors-15-24662-s001.pdf]

OPEN ACCESS

sensors

ISSN 1424-8220

www.mdpi.com/journal/sensors

Supplementary Information

FRET-Based Nanobiosensors for Imaging Intracellular Ca^{2+} and H^+ Microdomains. *Sensors* 2015, *15*, 24662-24680

Alsu I. Zamaleeva [1,†], Guillaume Despras [2,†], Camilla Luccardini [1], Mayeul Collot [2], Michel de Waard [3], Martin Oheim [4], Jean-Maurice Mallet [2] and Anne Feltz [1,*]

[1] Ecole Normale Supérieure, Institut de Biologie de l'ENS (IBENS), Inserm U1024, CNRS UMR 8197, Paris F-75005, France; E-Mails: alsu.zamaleeva@curie.fr (A.I.Z.); Camilla.luccardini@univ-lyon1.fr (C.L.)

[2] Department de Chimie, École Normale Supérieure-PSL Research University, CNRS UMR 7203 LBM, 24, rue Lhomond, and Sorbonne University, UPMC Univ Paris 06 LBM, 4 place Jussieu, Paris F-75005, France; E-Mails: gdespras@oc.uni-kiel.de (G.D.); mayeul.collot@unistra.fr (M.C.); jean-maurice.mallet@ens.fr (J.-M.M.)

[3] Inserm U836, Grenoble Neuroscience Institute, Research Group 3, LabEx Ion Channel Science and Therapeutics, Joseph Fourier University, BP170, Grenoble Cedex 09 38042, France; E-Mail: Michel.dewaard@ujf-grenoble.fr

[4] Brain Physiology Laboratory, CNRS UMR 8118, Faculté des Sciences Fondamentales et Biomédicales, Fédération de Neurosciences FR3636, Paris Descartes University, PRES Sorbonne Paris Cité, Paris F-75006, France; E-Mail: Martin.oheim@parisdescartes.fr

† These authors contributed equally to this work.

* Author to whom correspondence should be addressed; E-Mail: anne.feltz@ens.fr; Tel: +33-144-323-736; Fax: +33-144-323-887.

1. Supplementary Figures

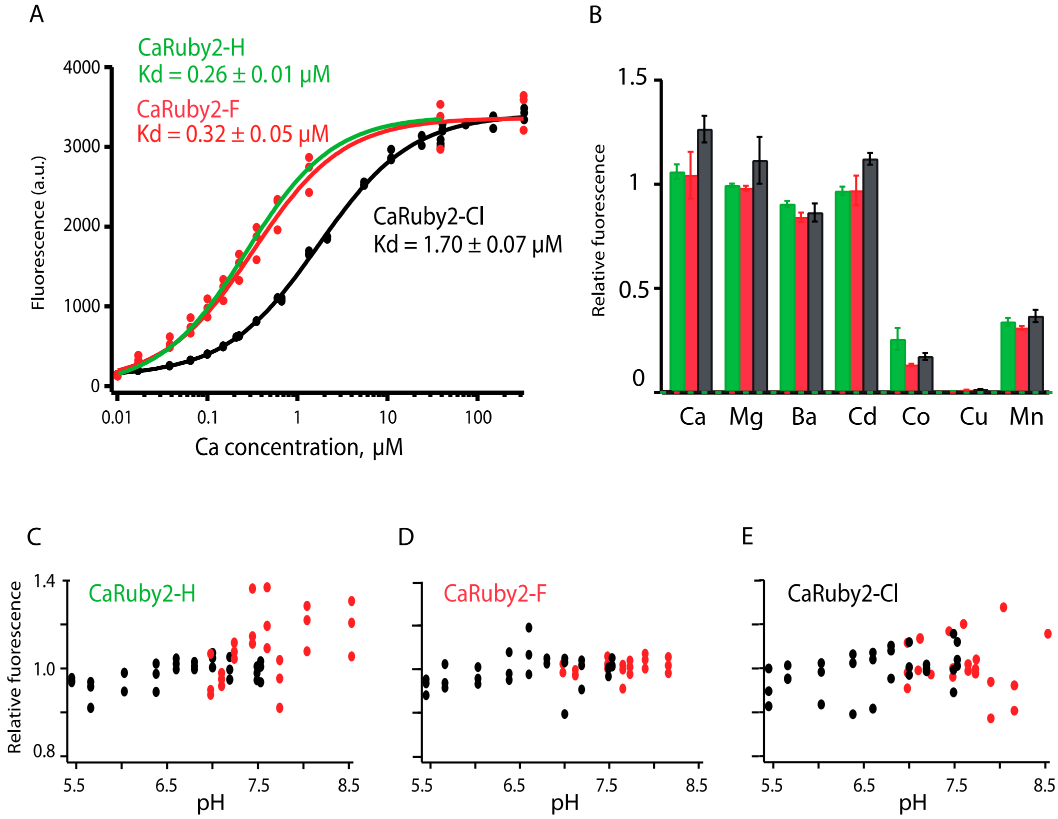

Figure S1. Photophysical properties of CaRubies-2. (**A**) [Ca^{2+}]-dependent changes of CaRubies fluorescence (green, black and red for H, F and Cl substituents respectively). Titration curves for the 3 CaRubies (same colour code) yielded Kd of 0.26 ± 0.01 µM, 0.32 ± 0.05 µM, and 1.70 ± 0.07 µM respectively. Experiments in triplicate. Ca^{2+} concentration was adjusted using the Invitrogen Ca Buffer kit (Life Technologies, ref: C-3008MP) ($[Ca^{2+}]_{free}$: 0 nM, 17 nM, 38 nM, 65 nM, 100 nM, 150 nM, 225 nM, 351 nM, 602 nM, 1.35 µM, 39 µM); (**B**) Normalized fluorescence of 5 µM CaRubies-2 in solutions containing ~ 5 µM free Ca^{2+} upon addition of 5 µM of either Ca^{2+}, Ba^{2+}, Cd^{2+}, Co^{2+}, Cu^{2+}, Mn^{2+} relative to that in the presence of ~ 5 µM free Ca^{2+} (in fact a nominally zero Ca^{2+} solution containing 100 mM KCl and 30 mM MOPS, and pH adjusted to 7.2 by KOH addition with no EGTA nor NTA added, 20–23 °C) All CaRubies-2 were highly selective for Ca^{2+} (with comparable fluorescence only for Ba^{2+} and Cd^{2+}, and reduced sensitivity in presence of Co^{2+} and Mn^{2+}). As with the first series of CaRubies, Cu^{2+} acted as potent quencher, making it a useful tool for estimating the amount of non-CaRuby related red fluorescence; (**C**–**E**) pH-dependence of the fluorescence of CaRubies-2, CaRuby2-H, CaRu2-F, CaRu2-Cl from from (**C**) to (**E**). respectively. Between pH 5.45 and 8.15 fluorescence varied from 0.816 to 1.39 (unity at pH 7.2). Symbols show mean ± SD from three independent titrations and fit. Buffer was MES for the most acidic pH values (black dots) and MOPS (red dots) for the more basic values.

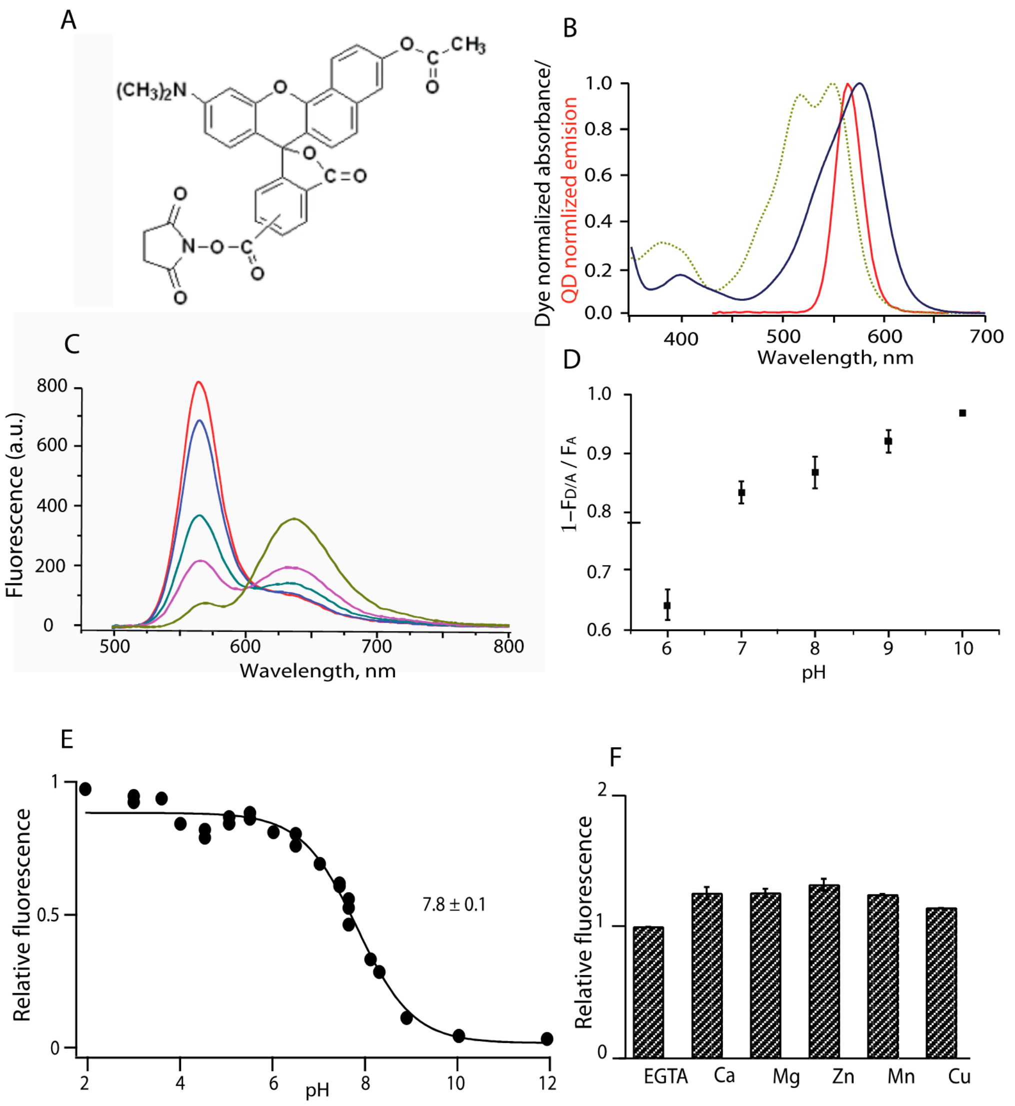

Figure S2. (**A–D**): A FRET-based Qdot565-SNARF-1 pair. (**A**) SNARF-1 NHS acceptor formula; (**B**) its absorbance spectra in green and black when pH is changed from 6 to 10 respectively in a 100 mM TRIS buffer. At basic pH SNARF-1 NHS shows increased peak amplitude as well as a 60 nm shift to the red wavelengths. In red, the emission spectrum (peak @ 565 nm) of carboxy-coated QuantumDots™; (**C**) Fluorescence measurements of the Qdot565-SNARF-1 pair emission spectrum upon 450 nm excitation when pH is changed from 6 to 10 in 100 mM Tris buffer. Donor quenching dominates over sensitized SNARF-1 acceptor fluorescence; (**D**) FRET efficiency of the pair increases as a function of pH (3 experiments, mean ± SE); (**E**,**F**): Photophysical properties of HR-PiAC; (**E**) Fluorometric titration of PEG5-HR-PiAC when pH is varied. Hill plot yields a K_D of 7.8 ± 0.1 identical to free dye K_D; (**F**) Normalized fluorescence of HR-PiAC when exposed to EGTA or to a range of divalent cations at 1 mM in a MOPS buffer (MOPS 30 µM, KCl 100 µM, pH 7.2; three batches).

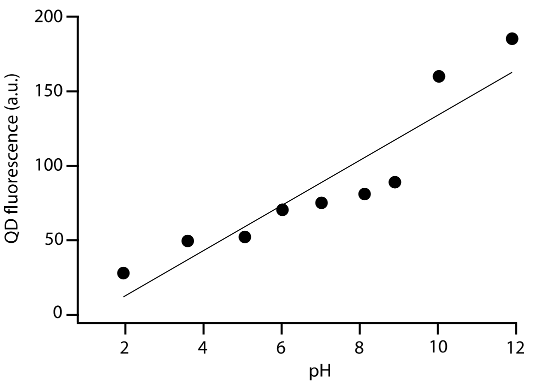

Figure S3. QDKCs sensitivity to pH. If QDKC absorbance did not display any pH sensitivity, integral of QDs emission measured between 500 and 580 nm proved to vary almost linearly with pH with a change by a factor 2.8 between pH 4 and 10.

2. Supplementary Material

2.1. Synthesis and Purification of CaRuby2-F and CaRuby2-Cl

2.1.1. Synthesis Scheme

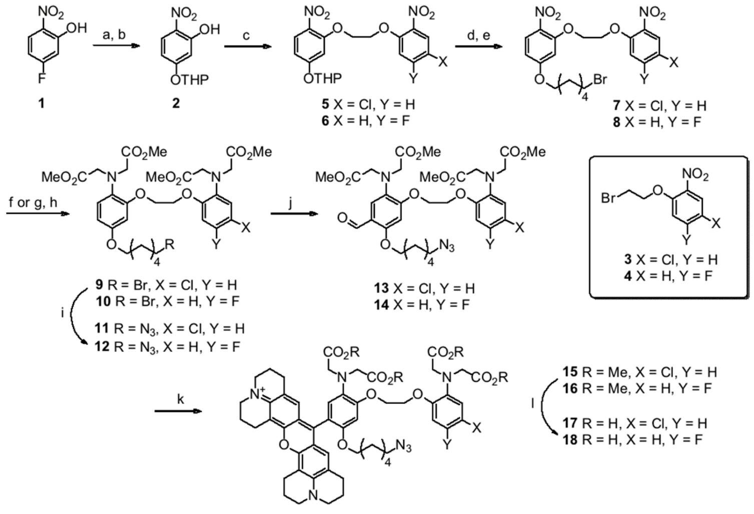

Scheme S1. Reagents and Conditions: (**a**) KOH (5 equiv), water, 90–100 °C, 93%; (**b**) 3,4-dihydropyran (2.5 equiv), camphorsulfonic acid (0.05 equiv), DCM, 0 °C, 86%; (**c**) **3** or **4** (1.1 equiv), K_2CO_3 (1.5 equiv), DMF, 70 °C, 85% for **5**, 81% for **6**; (**d**) Conc. HCl, THF, rt; (**e**) 1,6-dibromohexane (3 equiv), K_2CO_3 (3 equiv), DMF, 70 °C, 59% for **7**, 83% for **8** over two steps; (**f**) With substrate **7**: $SnCl_2 \cdot 2H_2O$ (8 equiv), conc. HCl, EtOH, 80 °C; (**g**) With substrate **8**: H_2, Pd/C, AcOEt/MeOH (4:1), rt; (**h**) $BrCH_2CO_2Me$ (12–15 equiv), DIEA (12–15 equiv), acetonitrile, 80 °C, 48% for **9**, 42% for **10** over two steps; (**i**) NaN_3 (3 equiv), DMF, 80 °C, 92% for **11**, 97% for **12**; (**j**) Vilsmeier reagent (3 equiv), DMF, 60 °C, 55% for **13**, 56% for **14**; (**k**) 8-hydroxyjulolidine (2 equiv), TfOH (0.15–0.3 equiv), DCM, rt, then *p*-chloranil (1 equiv), rt, 43% for **15**, 39% for **16**; (**l**) 10 M KOH, MeOH, rt, 40% for **17**, 77% for **18**. (THP = tetrahydropyranyl).

2.1.2. Synthesis of Compounds 1–4

4-nitroresorcinol (1):

To a solution of potassium hydroxide (42.9 g, 764 mmol) in water (150 mL) was added portionwise 5-fluoro-2-nitrophenol (24.0 g, 153 mmol). The mixture was heated at 90 °C for 24 h then the temperature was raised up to 100 °C. After refluxing for 19 h, the orange solution was cooled to room temperature then diluted in water and washed with aq. 1M HCl. The aqueous layer was extracted with ethyl acetate then the combined organic layers were washed with brine then dried over $MgSO_4$, filtered and concentrated to afford **1** (22 g, 93%) as a pale orange solid. Analytical and spectroscopic data were found to be in agreement with reported literature [1].

2-nitro-5-((tetrahydro-2H-pyran-2-yl)oxy)phenol (2):

A solution of **1** (4.75 g, 30.64 mmol) and 3,4-dihydropyran (7 mL, 76.61 mmol) in CH_2Cl_2 (150 mL) was cooled to 0 °C then camphorsulfonic acid (0.355 g, 1.53 mmol) was added. The yellow solution was stirred at 0 °C for 20 min then triethylamine (0.300 mL) was added and the mixture was concentrated. The residue was taken up in CH_2Cl_2 (50 mL) then hexane (400 mL) was added in order to precipitate the product. After 2 h at room temperature then 2 days at −18 °C, the solid was filtered then purified by flash chromatography (cyclohexane/ethyl acetate 95:5 to 92:8) to afford **2** (6.29 g, 86%) as a yellow solid. Analytical and spectroscopic data were found to be in agreement with reported literature [2].

1-(2-bromoethoxy)-4-chloro-2-nitrobenzene (3):

To a solution of 4-chloro-2-nitrophenol (20 g, 0.115 mmol) in *N*,*N*-dimethylformamide (100 mL) was added 1,2-dibromoethane (50 mL, 576 mmol) then potassium carbonate (32 g, 230 mmol). The mixture was heated at 70 °C for 2 h 30 min, cooled to room temperature then diluted with ethyl acetate and filtered through a celite pad. The filtrate was concentrated to dryness then taken up in ethyl acetate, washed with brine and dried over $MgSO_4$, filtered and concentrated. The residue was purified by flash chromatography (cyclohexane/ethyl acetate 9:1 to 85:15) to afford **3** (21.6 g, 67%) as a yellowish solid; ^{1}H NMR ($CDCl_3$, 300 MHz) δ 7.85 (d, J = 2.7 Hz, 1H), 7.50 (dd, J = 9.0 Hz, J = 2.7 Hz, 1H), 7.05 (d, J = 9.0 Hz, 1H), 4.40 (t, J = 6.4 Hz, 2H), 3.67 (t, J = 6.4 Hz, 2H); ^{13}C NMR ($CDCl_3$, 75 MHz) δ 150.2, 134.1, 126.6, 125.8, 116.8, 70.2, 27.9; HRMS: *m/z* calcd for $C_8H_7BrClNO_3Na$: 301.9190 [M+Na]$^+$, found 301.9192.

1-(2-bromoethoxy)-5-fluoro-2-nitrobenzene (4):

5-fluoro-2-nitrophenol (10.3 g, 65.56 mmol) was treated following the procedure which gave **3** to afford **4** (9.91 g, 57%) as a yellowish solid after flash chromatography (cyclohexane/ethyl acetate 9:1 to 85:15). Analytical and spectroscopic data were found to be in agreement with reported literature [3].

2.1.3. Synthesis Scheme of Nitro Compounds 5–8

2-(3-(2-(4-chloro-2-nitrophenoxy)ethoxy)-4-nitrophenoxy)tetrahydro-2H-pyran (5):

To a solution of 2 (3.86 g, 16.14 mmol) and **3** (4.98 g, 17.75 mmol) in *N*,*N*-dimethylformamide (20 mL) was added potassium carbonate (3.34 g, 24.2 mmol). The mixture was heated overnight at 70 °C then cooled to room temperature, diluted in ethyl acetate and filtered through a celite pad. The filtrate was concentrated to dryness then the residue was taken up in ethyl acetate and washed with aq. 1 M HCl. The aqueous layer was extracted with ethyl acetate then the combined organic layers were washed with brine then dried over $MgSO_4$, filtered and concentrated. The residue was recrystallized from ethyl acetate/petroleum ether then the solid was filtered off and washed with cold petroleum ether to afford **5** (6.04 g, 85%) as a yellow solid; ^{1}H NMR ($CDCl_3$, 300 MHz) δ 7.95 (d, *J* = 9.1 Hz, 1H), 7.81 (d, *J* = 2.5 Hz, 1H), 7.54 (dd, *J* = 8.9 Hz, *J* = 2.6 Hz, 1H), 7.31 (d, *J* = 9.0 Hz, 1H), 6.78 (d, *J* = 2.5 Hz, 1H), 6.73 (dd, *J* = 9.1 Hz, *J* = 2.4 Hz, 1H), 5.54–5.52 (m, 1H), 4.58–4.45 (m, 4H), 3.85–3.77 (m, 1H), 3.67–3.60 (m, 1H), 2.06–1.86 (m, 3H), 1.74–1.59 (m, 3H); ^{13}C NMR ($CDCl_3$, 75 MHz) δ 162.3, 161.6, 154.7, 154.1, 150.7, 150.6, 140.4, 134.4, 134.1, 133.5, 128.5, 128.1, 126.4, 125.5, 125.3, 117.8, 117.0, 108.6, 103.0, 102.7, 96.4, 94.7, 69.5, 69.1, 68.6, 68.4, 61.9, 29.8, 24.9, 1; HRMS: *m/z* calcd for $C_{19}H_{19}ClN_2O_8Na$: 461.0722 $[M + Na]^+$, found 461.0724.

2-(3-(2-(5-fluoro-2-nitrophenoxy)ethoxy)-4-nitrophenoxy)tetrahydro-2H-pyran (6):

Compounds **2** (5.38 g, 22.49 mmol) and 4 (6.53 g, 24.74 mmol) were treated following the procedure which gave **5** to afford **6** (9.50 g, 81%) as a yellow solid after flash chromatography (cyclohexane/ethyl acetate 9:1 to 4:1); ^{1}H NMR ($CDCl_3$, 300 MHz) δ 7.95–7.90 (m, 2H), 6.98 (dd, *J* = 10.1 Hz, J = 1.5 Hz, 1H), 6.81–6.71 (m, 3H), 5.54–5.52 (m, 1H), 4.56–4.48 (m, 4H), 3.85–3.77 (m, 1H), 3.67–3.61 (m, 1H), 2.06–1.87 (m, 3H), 1.74–1.60 (m, 3H); ^{13}C NMR ($CDCl_3$, 75 MHz) δ 167.3, 163.9, 162.2, 154.2, 154.1, 136.4, 133.7, 128.0, 127.8, 108.7, 108.5, 108.1, 103.8, 103.4, 96.4, 68.8, 68.3, 61.9, 29.8, 24.9, 18.1: HRMS: *m/z* calcd for $C_{19}H_{19}FN_2O_8Na$: 445.1018 $[M + Na]^+$, found 445.1019.

4-((6-bromohexyl)oxy)-2-(2-(4-chloro-2-nitrophenoxy)ethoxy)-1-nitrobenzene (7):

To a solution of **5** (6.04 g, 13.76 mmol) in a 2:1 mixture of THF/water (150 mL) was added conc. HCl (15 mL). The solution was stirred at room temperature for 1 h 30 min then diluted in ethyl acetate and washed with brine. The aqueous layer was extracted with ethyl acetate then the combined organic layers were dried over $MgSO_4$, filtered and concentrated to dryness. The residue was dissolved in *N*,*N*-dimethylformamide (45 mL) then 1,6-dibromohexane (6.30 mL, 41.28 mmol) and potassium carbonate (2.85 g, 20.64 mmol) were added. The mixture was stirred at 70 °C for 2 h 30 min then diluted with ethyl acetate and filtered through a celite pad. The filtrate was concentrated then taken up in CH_2Cl_2 and washed with aq. 1M HCl. The aqueous layer was extracted with CH_2Cl_2 then the combined organic layers were washed with brine then dried over $MgSO_4$, filtered and concentrated. The residue was purified by flash chromatography (cyclohexane/ethyl acetate 95:5 to 85:15) then the residue was taken up in ethyl acetate and hexane (200 mL) was added. After triturating for 10 min then cooling at −25 °C for 1 h, the precipitate was filtered off to afford **7** (4.21 g, 59%) as an off white solid; ^{1}H NMR ($CDCl_3$, 300 MHz) δ 7.97 (d, *J* = 9.1 Hz, 1H), 7.82 (d, *J* = 2.5 Hz, 1H), 7.55 (dd,

J = 9.0 Hz, *J* = 2.6 Hz, 1H), 7.29 (d, *J* = 8.9 Hz, 1H), 6.60 (d, *J* = 2.5 Hz, 1H), 6.55 (dd, *J* = 9.1 Hz, *J* = 2.5 Hz, 1H), 4.57–4.46 (m, 4H), 4.04 (t, *J* = 6.3 Hz, 2H), 3.44 (t, *J* = 6.8 Hz, 2H), 1.95–1.79 (m, 4H), 1.55–1.50 (m, 4H); ^{13}C NMR ($CDCl_3$, 75 MHz) δ 164.4, 154.6, 150.8, 140.6, 134.3, 133.4, 128.5, 126.7, 125.5, 117.9, 106.9, 102.1, 69.3, 68.9, 68.8, 33.9, 32.7, 29.0, 28.0, 25.3; HRMS: *m/z* calcd for $C_{20}H_{22}BrClN_2O_7Na$: 539.0191 [M + Na]$^+$, found 539.0191.

4-((6-bromohexyl)oxy)-2-(2-(5-fluoro-2-nitrophenoxy)ethoxy)-1-nitrobenzene (8):

Compound **6** (7.52 g, 17.80 mmol) was treated following the procedure which gave **7** to afford **8** (7.36 g, 83%) as a yellowish solid after flash chromatography (cyclohexane/ethyl acetate 9:1 to 4:1) followed by a precipitation from hexanes; ^{1}H NMR ($CDCl_3$, 300 MHz) δ 7.97–7.91 (m, 2H), 6.97 (dd, *J* = 10.1 Hz, *J* = 2.6 Hz, 1H), 6.81–6.74 (m, 1H), 6.63 (d, *J* = 2.4 Hz, 1H), 6.55 (dd, *J* = 9.2 Hz, *J* = 2.5 Hz, 1H), 4.56–4.48 (m, 4H), 4.05 (t, *J* = 6.4 Hz, 2H), 3.43 (t, *J* = 6.7 Hz, 2H), 1.95–1.79 (m, 4H), 1.57–1.45 (m, 4H); ^{13}C NMR ($CDCl_3$, 75 MHz) δ 167.5, 164.4, 164.1, 154.6, 154.4, 154.2, 136.6, 133.6, 128.4, 128.2, 128.0, 108.7, 108.4, 107.1, 103.9, 103.6, 102.4, 69.0, 68.8, 33.8 (2C), 32.7, 32.6, 28.9, 27.9, 27.4, 25.3; HRMS: *m/z* calcd for $C_{20}H_{22}BrFN_2O_7Na$: 523.0487 [M + Na]$^+$, found 523.0487.

2.1.4. Synthesis of BAPTA Derivatives 9–14

Dimethyl 2,2'-((2-(2-(2-(bis(2-methoxy-2-oxoethyl)amino)-4-chlorophenoxy)ethoxy)-4-((6-bromohexyl)oxy)phenyl)azanediyl)diacetate (9):

To a suspension of **7** (2.59 g, 5.0 mmol) in absolute ethanol (40 mL) was added $SnCl_2 \cdot 2H_2O$ (9.0 g, 40 mmol) and conc. HCl (6.5 mL). The mixture was stirred in the dark at 80 °C for 2 h then cooled to room temperature and brought to Ph > 11with dropwise addition of aq. 3 M NaOH. A grey precipitate started forming and the solution turned gradually from yellow to reddish. The resulting suspension was diluted with water then extracted with diethyl ether (5 × 100 mL). The combined organic layers were dried over $MgSO_4$, filtered and concentrated to dryness to afford the crude amino derivative as a dark brown oil. The residue was dissolved in acetonitrile (10 mL) then methyl bromoacetate (7.1 mL, 75 mmol) and *N*,*N*-diisopropylethylamine (13.1 mL, 75 mmol) were added. The mixture was stirred in the dark and under argon at 80 °C for 38 h then cooled to room temperature. The mixture was diluted with CH_2Cl_2 then washed with satd. aq. $NaHCO_3$ and the aqueous layer was extracted with CH_2Cl_2. The combined organic layers were dried over $MgSO_4$, filtered and concentrated to dryness. The crude residue was purified by flash chromatography (cyclohexane/ethyl acetate 9:1 to 7:3) to afford **9** (1.78 g, 48%) as a brownish syrup; ^{1}H NMR ($CDCl_3$, 300 MHz) δ 6.88–6.76 (m, 4H), 6.44 (d, J = 2.7 Hz, 1H), 6.38 (dd, J = 8.7 Hz, J = 2.7 Hz, 1H), 4.24 (s, 4H), 4.13 (s, 4H), 4.08 (s, 4H), 3.88 (t, J = 6.4 Hz, 2H), 3.58 (s, 6H), 3.57 (s, 6H), 3.42 (t, J = 6.8 Hz, 2H), 1.93–1.84 (m, 2H), 1.80–1.71 (m, 2H), 1.55–1.43 (m, 4H); ^{13}C NMR ($CDCl_3$, 75 MHz) δ 172.2, 171.7, 155.3, 151.9, 149.1, 140.5, 133.0, 126.5, 121.8, 120.7, 119.2, 114.2, 105.4, 101.8, 68.2, 67.6, 67.0, 53.7, 53.3, 51.9, 51.7, 34.0, 32.8, 29.3, 28.1, 25.4; HRMS: *m/z* calcd for $C_{32}H_{42}BrClN_2O_{11}Na$: 767.1553 [M + Na]$^+$, found 767.1554.

Dimethyl 2,2'-((2-(2-(2-(bis(2-methoxy-2-oxoethyl)amino)-5-((6-bromohexyl)oxy)phenoxy)ethoxy)-4-fluorophenyl)azanediyl)diacetate (10):

To a solution of **8** (0.490 g, 0.977 mmol) in a 4:1 mixture of ethyl acetate/methanol (10 mL) was added 10% w/w palladium on carbon (0.100 g). The suspension was stirred at room temperature under hydrogen atmosphere for 3 h then filtered through a celite pad. The filtrate was concentrated to dryness to afford the crude amino derivative as a dark brown oil. The residue was dissolved in acetonitrile (2 mL) then methyl bromoacetate (1.1 mL, 11.7 mmol) and *N*,*N*-diisopropylethylamine (2 mL, 11.7 mmol) were added. The mixture was stirred in the dark and under argon at 80 °C for 20 h then cooled to room temperature. The mixture was diluted with CH_2Cl_2 then washed with satd. aq. $NaHCO_3$ and the aqueous layer was extracted with CH_2Cl_2. The combined organic layers were dried over $MgSO_4$, filtered and concentrated to dryness. The crude residue was purified by flash chromatography (cyclohexane/ethyl acetate 9:1 to 7:3) to afford **9** (0.301 g, 42%) as a brownish syrup; ^{1}H NMR ($CDCl_3$, 300 MHz) δ 6.86–6.82 (m, 2H), 6.65–6.54 (m, 2H), 6.46 (d, *J* = 2.6 Hz, 1H), 6.39 (dd, *J* = 8.7 Hz, J = 2.7 Hz, 1H), 4.28 (s, 4H), 4.09 (s, 4H), 4.08 (s, 4H), 3.88 (t, J = 6.4 Hz, 2H), 3.58 (d, J = 1.7 Hz, 6H), 3.42 (t, J = 6.8 Hz, 2H), 1.94–1.85 (m, 2H), 1.80–1.72 (m, 2H), 1.54–1.45 (m, 4H); ^{13}C NMR ($CDCl_3$, 75 MHz) δ 172.1, 171.9, 160.3, 157.1, 155.4, 151.9, 151.8, 151.7, 135.6 (2C), 133.1, 120.9, 120.5, 120.4, 107.3, 107.0, 105.7, 102.1, 101.7, 101.4, 68.2, 67.5, 67.1, 55.1, 53.8, 53.6, 51.7, 51.6, 45.1, 33.9, 32.8, 32.6, 29.3, 28.0, 26.8, 25.5, 25.4; HRMS: *m/z* calcd for $C_{32}H_{42}BrFN_2O_{11}Na$: 751.1848 $[M + Na]^+$, found 751.1852.

Dimethyl 2,2'-((4-((6-azidohexyl)oxy)-2-(2-(2-(bis(2-methoxy-2-oxoethyl)amino)-4-chlorophenoxy)ethoxy)phenyl)azanediyl)diacetate (11):

To a solution of **9** (1.02 g, 1.37 mmol) in *N*,*N*-dimethylformamide (6 mL) was added sodium azide (0.270 g, 3.06 mmol). The solution was stirred in the dark and under argon at 80 °C for 21 h then cooled to room temperature and diluted with ethyl acetate. After washing twice with water, the combined aqueous layers were extracted with ethyl acetate. The combined organic layers were washed with brine then dried over $MgSO_4$, filtered and concentrated to dryness to afford **11** (0.897 g, 92%) as a brownish syrup; ^{1}H NMR ($CDCl_3$, 300 MHz) δ 6.89–6.76 (m, 4H), 6.44 (d, *J* = 2.6 Hz, 1H), 6.38 (dd, *J* = 8.7 Hz, *J* = 2.6 Hz, 1H), 4.24 (s, 4H), 4.13 (s, 4H), 4.08 (s, 4H), 3.88 (t, *J* = 6.5 Hz, 2H), 3.59 (s, 6H), 3.57 (s, 6H), 3.28 (t, *J* = 6.8 Hz, 2H), 1.80–1.71 (m, 2H), 1.68–1.59 (m, 2H), 1.53–1.40 (m, 4H); ^{13}C NMR ($CDCl_3$, 75 MHz) δ 172.2, 171.7, 155.3, 151.9, 149.1, 140.5, 133.0, 126.5, 121.8, 120.7, 119.2, 114.2, 105.4, 101.8, 68.2, 67.6, 67.0, 53.7, 53.3, 51.9, 51.7, 51.5, 29.3, 28.9, 28.1, 26.7, 25.8; HRMS: *m/z* calcd for $C_{32}H_{42}ClN_5O_{11}Na$: 730.2462 $[M + Na]^+$, found 730.2463.

Dimethyl 2,2'-((4-((6-azidohexyl)oxy)-2-(2-(2-(bis(2-methoxy-2-oxoethyl)amino)-5-fluorophenoxy)ethoxy)phenyl)azanediyl)diacetate (12):

Compound 10 (0.287 g, 0.393 mmol) was treated following the procedure which gave **11** to afford **12** (0.264 g, 97%) as a brownish syrup; ^{1}H NMR ($CDCl_3$, 300 MHz) δ 6.86–6.81 (m, 2H), 6.65–6.54 (m, 2H), 6.46 (d, *J* = 2.6 Hz, 1H), 6.39 (dd, *J* = 8.7 Hz, *J* = 2.6 Hz, 1H), 4.27 (s, 4H), 4.09 (s, 4H), 4.08 (s, 4H), 3.88 (t, *J* = 6.4 Hz, 2H), 3.58 (s, 6H), 3.57 (s, 6H), 3.28 (t, *J* = 6.8 Hz, 2H), 1.80–1.71 (m, 2H), 1.68–1.59 (m, 2H), 1.54–1.39 (m, 4H); ^{13}C NMR ($CDCl_3$, 75 MHz) δ 172.1, 172.0, 160.3, 157.1, 155.4, 151.9, 151.8, 151.7, 135.6 (2C), 133.1, 129.2, 128.4, 125.4, 120.9, 120.5, 120.4, 107.3, 107.0,

105.6, 102.1, 101.7, 101.3, 68.2, 67.5, 67.1, 53.8, 53.6, 51.8, 51.7, 51.5, 29.3, 28.9, 26.7, 25.8; HRMS: *m/z* calcd for $C_{32}H_{42}FN_5O_{11}Na$: 714.2757 $[M + Na]^+$, found 714.2757.

Dimethyl 2,2'-((4-((6-azidohexyl)oxy)-2-(2-(2-(bis(2-methoxy-2-oxoethyl)amino)-4-chlorophenoxy) ethoxy)-5-formylphenyl)azanediyl)diacetate (13):

A solution of phosphoryl chloride (0.350 mL, 3.73 mmol) in *N,N*-dimethylformamide (0.700 mL) was stirred at 0 °C for 1 h then added dropwise to a solution of **11** (0.880 g, 1.24 mmol) in *N,N*-dimethylformamide (4 mL). The mixture was stirred in the dark at 60 °C for 1 h 30 then cooled to room temperature before diluting with ethyl acetate and adding satd. aq. $NaHCO_3$. The aqueous layer was extracted with ethyl acetate then the combined organic layers were washed with brine, dried over $MgSO_4$, filtered and concentrated. The crude residue was purified by flash chromatography (cyclohexane/ethyl acetate 9:1 to 3:2) to afford **13** (0.506 g, 55%) as an orange syrup; ^1H NMR ($CDCl_3$, 300 MHz) δ 10.29 (s, 1H), 7.34 (s, 1H), 6.87 (dd, *J* = 8.6 Hz, *J* = 2.4 Hz, 1H), 6.79–6.76 (m, 2H), 6.44 (s, 1H), 4.32–4.27 (m, 4H), 4.10 (s, 4H), 4.06 (s, 4H), 4.00 (t, *J* = 6.4 Hz, 2H), 3.58 (s, 6H), 3.57 (s, 6H), 3.28 (t, *J* = 6.8 Hz, 2H), 1.87–1.78 (m, 2H), 1.67–1.58 (m, 2H), 1.53–1.43 (m, 4H); ^{13}C NMR ($CDCl_3$, 75 MHz) δ 188.0, 171.6, 171.4, 159.0, 157.4, 149.0, 140.7, 133.5, 126.9, 121.9, 119.5, 118.6, 118.5, 114.7, 98.2, 69.1, 67.5 (2C), 53.5, 53.4, 51.8 (2C), 51.4, 29.2, 28.9, 26.6, 25.8; HRMS: *m/z* calcd for $C_{33}H_{42}ClN_5O_{12}Na$: 758.2411 $[M + Na]^+$, found 758.2413.

Dimethyl 2,2'-((4-((6-azidohexyl)oxy)-2-(2-(2-(bis(2-methoxy-2-oxoethyl)amino)-5-fluorophenoxy) ethoxy)-5-formylphenyl)azanediyl)diacetate (14):

Compound **12** (0.234 g, 0.338 mmol) was treated following the procedure which gave **13** to afford **14** (0.135 g, 56%) as an orange oil after flash chromatography (cyclohexane/ethyl acetate 3:2 to 1:1); ^1H NMR (CD_3OD, 300 MHz) δ 10.30 (s, 1H), 7.35 (s, 1H), 6.85 (dd, *J* = 8.7 Hz, *J* = 5.9 Hz, 1H), 6.65 –6.56 (m, 2H), 6.45 (s, 1H), 4.38–4.28 (m, 4H), 4.07 (s, 4H), 4.06 (s, 4H), 4.02 (t, *J* = 6.4 Hz, 2H), 3.57 (s, 12H), 3.28 (t, *J* = 6.8 Hz, 2H), 1.88–1.79 (m, 2H), 1.68–1.59 (m, 2H), 1.54 1.41 (m, 4H); ^{13}C NMR ($CDCl_3$, 75 MHz) δ 188.1, 171.9, 171.7, 160.3, 159.0, 157.4, 157.1, 151.6, 151.5, 135.7, 133.6, 120.7, 120.6, 118.7, 118.6, 107.7, 107.5, 102.0, 101.7, 98.3, 69.1, 67.5, 67.3, 53.6, 53.5, 51.8, 51.7, 51.5, 29.2, 28.9, 26.6, 25.8; HRMS: *m/z* calcd for $C_{33}H_{42}FN_5O_{12}Na$: 742.2706 $[M + Na]^+$, found 742.2706.

2.1.5. Synthesis of CaRu2 Probes 15–18

CaRuby2-Cl-methyl ester (15):

To a solution of **13** (0.110 g, 0.149 mmol) in CH_2Cl_2 (1 mL) was added 8-hydroxyjulolidine (0.056 g, 0.299 mmol) then trifluoromethanesulfonic acid (4 µL, 0.045 mmol). The solution was stirred overnight in the dark at room temperature then p-chloranil (0.037 g, 0.149 mmol) was added and the brown solution turned dark. After stirring in the dark at room temperature for 4 h, the purple mixture was concentrated. The crude residue was purified by flash chromatography (CH_2Cl_2/methanol 100:0 to 95:5) to afford **15** (0.075 g, 43%) as a dark purple solid; ^1H NMR (CD_3OD, 300 MHz) δ 6.99–6.87 (m, 5H), 6.79–6.78 (m, 2H), 4.44–4.34 (m, 4H), 4.17 (s, 4H), 4.11 (s, 4H), 3.91 (t, *J* = 5.7 Hz, 2H), 3.64 (s, 6H), 3.56–3.51 (m, 12H), 3.09–3.03 (m, 6H), 2.86–2.67 (m, 4H), 2.16–2.07 (m, 4H),

2.02–1.94 (m, 4H), 1.47–1.39 (m, 2H), 1.34–1.25 (m, 2H), 1.15–1.04 (m, 2H), 1.01–0.94 (m, 2H); ^{13}C NMR (CD_3OD, 75 MHz) δ 173.4, 173.3, 154.6, 154.4, 153.7, 153.6, 152.4, 150.5, 141.9, 133.9, 128.3, 128.1, 127.5, 127.4, 124.9, 123.8, 122.5, 122.4, 119.8, 116.0, 115.9, 114.7, 114.5, 106.4, 101.4, 70.1, 69.1, 69.0, 68.8, 54.8, 54.6, 54.5, 52.3, 52.1, 51.9, 51.4, 30.0, 29.8, 28.7, 27.2, 26.7, 21.9, 21.0, 20.9; HRMS: *m/z* calcd for $C_{57}H_{67}ClN_7O_{12}^+$: 1076.4531 $[M]^+$, found 1076.4518.

CaRuby2-F-methyl ester (16):

Compound **14** (0.117 g, 0.162 mmol) was treated following the procedure which gave **15** to afford **16** (0.075 g, 39%) as a deep purple solid after flash chromatography (CH_2Cl_2/methanol 100:0 to 94:6); ^{1}H NMR (CD_3OD, 300 MHz) δ 6.94 (s, 2H), 6.91–6.80 (m, 4H), 6.60 (td, J = 8.2 Hz, J = 2.7 Hz, 1H), 4.48–4.36 (m, 4H), 4.12 (s, 4H), 4.10 (s, 4H), 3.92 (t, J = 5.7 Hz, 2H), 3.61 (s, 6H), 3.57–3.50 (m, 12H), 3.09–2.99 (m, 6H), 2.84–2.67 (m, 4H), 2.14–2.06 (m, 4H), 2.02–1.94 (m, 4H), 1.47–1.39 (m, 2H), 1.34–1.24 (m, 2H), 1.14–1.04 (m, 2H), 1.02–0.94 (m, 2H); ^{13}C NMR (CD_3OD, 75 MHz) δ 173.5, 173.3, 154.6, 154.4, 153.7, 153.6, 152.2, 153.0, 152.4, 137.0, 133.8, 128.3, 124.9, 123.7, 121.4, 121.3, 114.6, 114.5, 106.4, 101.3, 70.1, 68.9 (2C), 54.7 (2C), 52.2, 52.1, 51.8, 51.4, 29.9, 29.8, 28.7, 27.2, 26.7, 21.9, 21.0, 20.9; HRMS: *m/z* calcd for $C_{57}H_{67}FN_7O_{12}^+$: 1060.4826 $[M]^+$, found 1060.4813.

CaRuby2-Cl (17):

To a solution of **15** (0.114 g, 0.105 mmol) in methanol (7 mL) was added aq. 10 M KOH (1 mL). The mixture was stirred in the dark at room temperature for 20 h then diluted with chloroform and washed with aq. 1 M HCl. The aqueous layer was extracted with chloroform then the combined organic layers were dried over $MgSO_4$, filtered and concentrated. The crude residue was purified on a reverse phase column C-18 using acetonitrile (0, 1% TFA) and water (0, 1% TFA) as eluant (20% ACN to 80%) to afford **17** (0.043 g, 40%) as a deep purple solid after lyophilization (water/ dioxane 1:1); HRMS: *m/z* calcd for $C_{53}H_{59}ClN_7O_{12}^+$: 1020.3905 $[M]^+$, found 1020.3902.

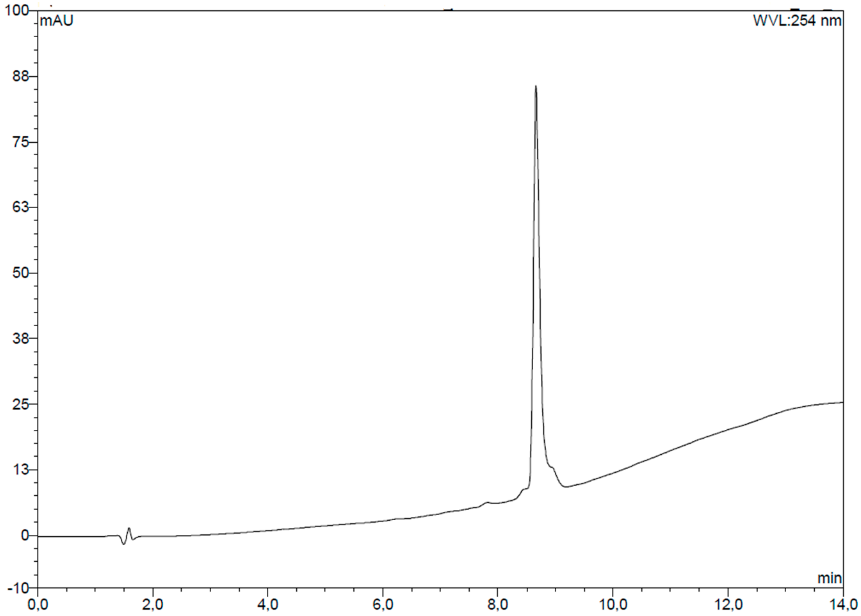

Figure S4. HPLC chromatogram of **17**.

CaRu2-F (18):

To a solution of **16** (0.055 g, 0.052 mmol) in methanol (4 mL) was added aq. 10 M KOH (0.5 mL). The mixture was stirred in the dark at room temperature for 2 h then diluted with chloroform and washed with aq. 1 M HCl. The aqueous layer was extracted with chloroform then the combined organic layers were dried over $MgSO_4$, filtered and concentrated. The residue was taken up in a 1:1 mixture of water/dioxane then freeze-dried to afford **18** (0.040 g, 77%) as a deep purple solid; HRMS: *m/z* calcd for $C_{53}H_{59}FN_7O_{12}^+$: 1004.4200 $[M]^+$, found 1004.4196.

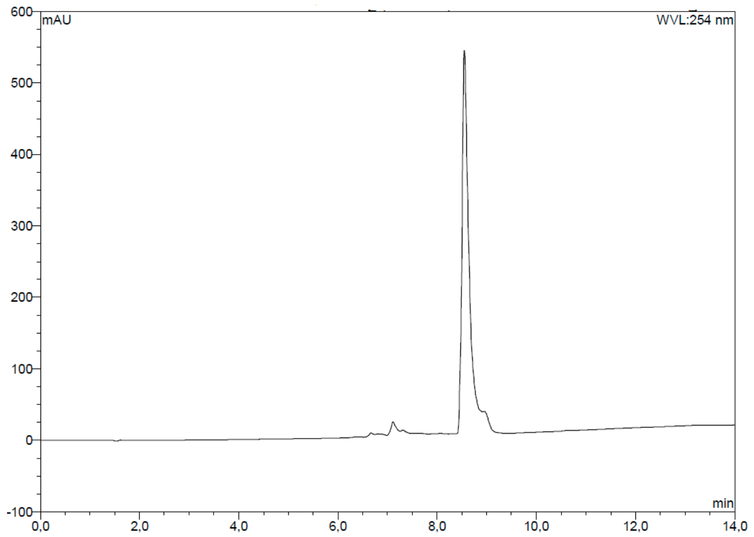

Figure S5. HPLC chromatogram of **18**.

2.1.6. Synthesis of the Amino PEG and Dextran Conjugates.

PEG-NH_2 conjugates: To a solution of NH_2-PEG-alkyne 5 kDa (15 mg, 3 µmol) and CaRu2-Cl/F (6 mg, 6 µmol) in methanol (2 mL) was added a mixture of $CuSO_4 \cdot 5H_2O$ (15 mg, 60 µmol) and sodium ascorbate (15 mg, 75 µmol) in water (0.5 mL). The mixture was stirred in the dark at room temperature for 20 h then diluted with water and extracted with DCM. The combined organic layers were washed with 0.1 M aq. EDTA then the aqueous layer was extracted with DCM. The combined organic layers were concentrated then the crude residue was purified over LH-20 size exclusion phase (elution with 1:1 DCM:methanol). The first colored fraction was concentrated, taken up in water then freeze-dried to give the conjugates **19** (Cl) (13 mg, 70%) and **20** (F) (11 mg, 60%) as purple solids.

Dextran conjugates: To a solution of dextran 6 kDa alkyne (in [4]: 30 mg, 3 µmol) and $CaRu_2$-Cl/F (8 mg, 8 µmol) in *N*,*N*-dimethylformamide (2.5 mL) was added a mixture of $CuSO_4.5H_2O$ (15 mg, 60 µmol) and sodium ascorbate (15 mg, 75 µmol) in water (0.5 mL). The mixture was stirred in the dark at room temperature for 20 h then concentrated. The crude residue was dissolved in aq. 0.1 M EDTA (0.5 mL) then purified over G-25 size exclusion column (elution with water) to give the conjugates **21** (Cl) (17 mg, Molar ratio ~ 1 mol dye /mol dextran) and **22** (F) (15 mg, Molar ratio ~ 1 mol dye /mol dextran) as purple solids after lyophilization.

3. Drugs and Chemicals

ACN (Sigma-Aldrich, St. Louis, MO, USA, 271004); APV (Tocris, Bristol, UK, 0106); 1 M $CaCl_2$ (Fluka, St. Gallen, Switzerland, 21115); Blasticidine S hydrochloride (Sigma-Aldrich, St. Louis, MO, USA, 15205); Coumarin 314 (Sigma-Aldrich, St. Louis, MO, USA, 392995); DIEA (Sigma-Aldrich, St. Louis, MO, USA, 387649); DMEM (Invitrogen, Waltham, MA, USA 31885); Ethanol (Merck, Kenilworth, NJ, USA, 100983); HEPES (Sigma-Aldrich, St. Louis, MO, USA, H3375); geneticine (Gibco, Waltham, MA, USA, 10131); glycine (Tocris, Bristol, UK, 0219); glutamate (Tocris, Bristol, UK, 0218); HOBt (Iris-Biotech, Marktredwitz, Germany, RL-1035); Methanol (Merck, Kenilworth, NJ, USA, 106009); MOPS (Sigma-Aldrich, St. Louis, MO, USA, M-1254); TFA (Sigma-Aldrich, St. Louis, MO, USA, T6508); water (Fluka, St. Gallen, Switzerland, Cat No. 95305).

Quantum dots (QDs): Hydrophobic TOP/TOPO coated CdSe/CdS/ZnS QDs (CANdots, Hamburg, Germany; λ_{Em} = 565 nm) sold in hexane were transferred to a toluene medium by slowly evaporating hexane under reduced pressure and kept as a µM stock solution at 4 °C.

4. Abbreviation List

APV, (2R)-amino-5-phosphonovaleric acid NMDAR antagonist; BAPTA,1,2-bis(o-aminophenoxy) ethane-*N*,*N*,*N'*,*N'*-tetra-acetic acid; BHK, baby hamster kidney cell line; Ca^{2+}, calcium (ion); CPP, cell penetrating peptide; CuAAC, Copper-Catalyzed Azide-Alkyne Cycloaddition; DCM, dichloro-methene; DMEM, Dulbecco's minimal essential medium; D, donor in a FRET pair; DIEA, *N*,*N*-Diisopropylethylamine; E, FRET efficacy estimated on the donor; EDTA, Ethylenediaminetetraacetic acid; EGTA, ethylene glycol tetraacetic acid; F, fluorescence intensity; FRET, Förster resonance energy transfer; GMBS, N-[γ-maleimidobutyryloxy]succinimide ester; Had_{UF1-11}, prepared from Hadrucalcin toxin; HEPES, 4-(2-hydroxyethyl)-1-piperazineethanesulfonic acid; MeOH, methanol; HOBt, hydroxybenzotriazole; MOPS, 3-(N-morpholino) propanesulfonic acid; NMDA, N-methyl-D-aspartate; NMDAR, NMDA-sensitive glutamate receptor type; pC, Ac-CGSESGGSESG(FCC)3F-amide peptide ; pK, NH_2-KGSESGGSESG(FCC)3F-amide peptide ; PEG, poly-ethylene glycol; QD, quantum dot, or fluorescent colloidal nanoparticle; TAE, Tris-acetate-EDTA buffer; TEM, transmission electron microscopy; TFA, trifluoroacetic acid; TOP/TOPO, Tri-n-octylphosphine/Tri-n-octylphosphine oxide.

5. Copies of NMR Spectra

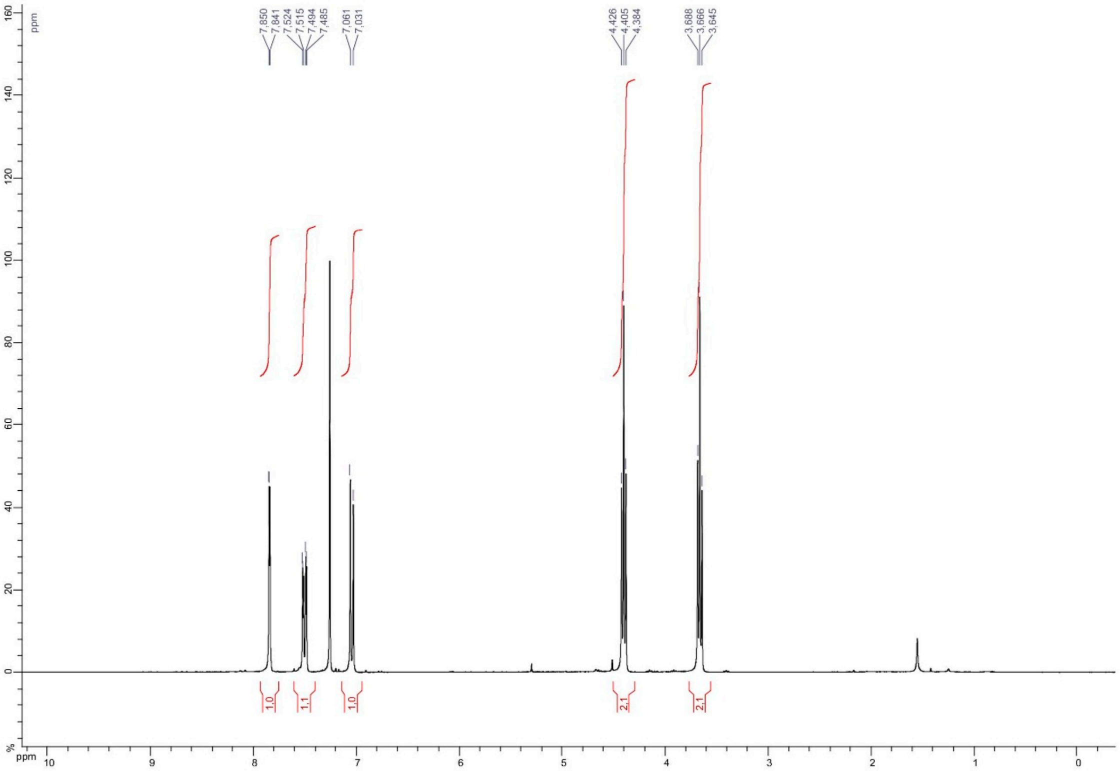

Figure S6. ^{1}H NMR spectrum of **3** ($CDCl_3$, 300 MHz).

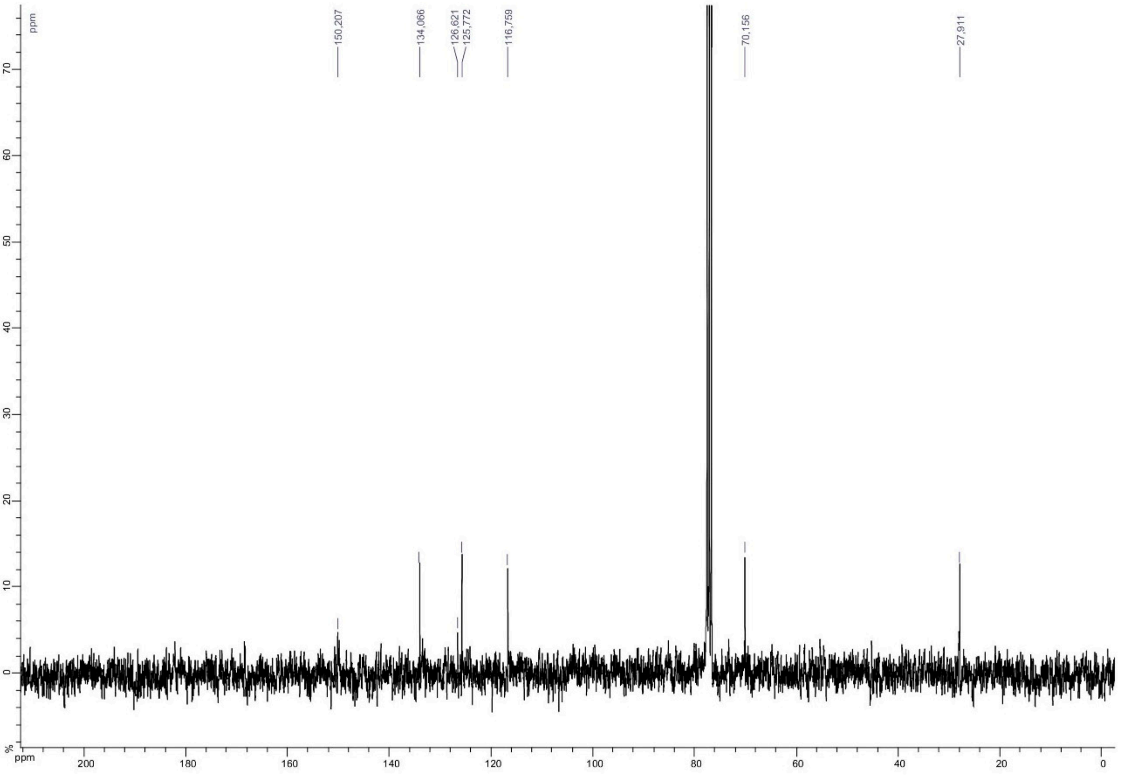

Figure S7. ^{13}C NMR spectrum of **3** ($CDCl_3$, 75 MHz).

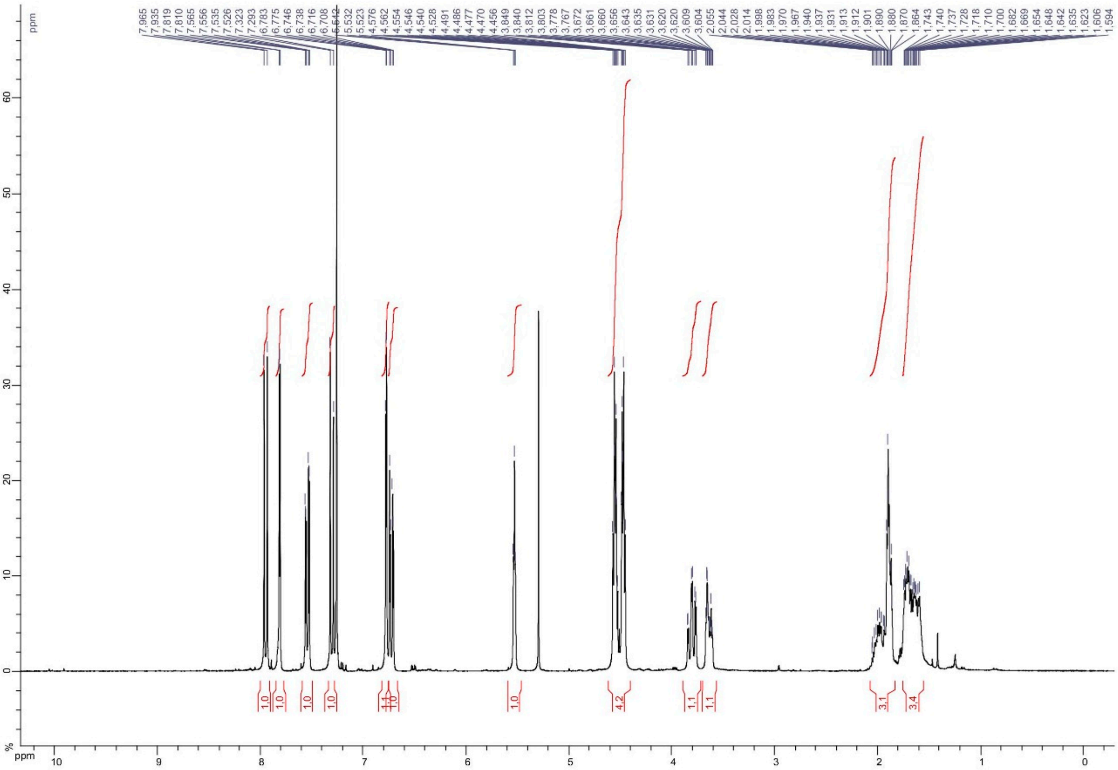

Figure S8. ^{1}H NMR spectrum of **5** ($CDCl_3$, 300 MHz).

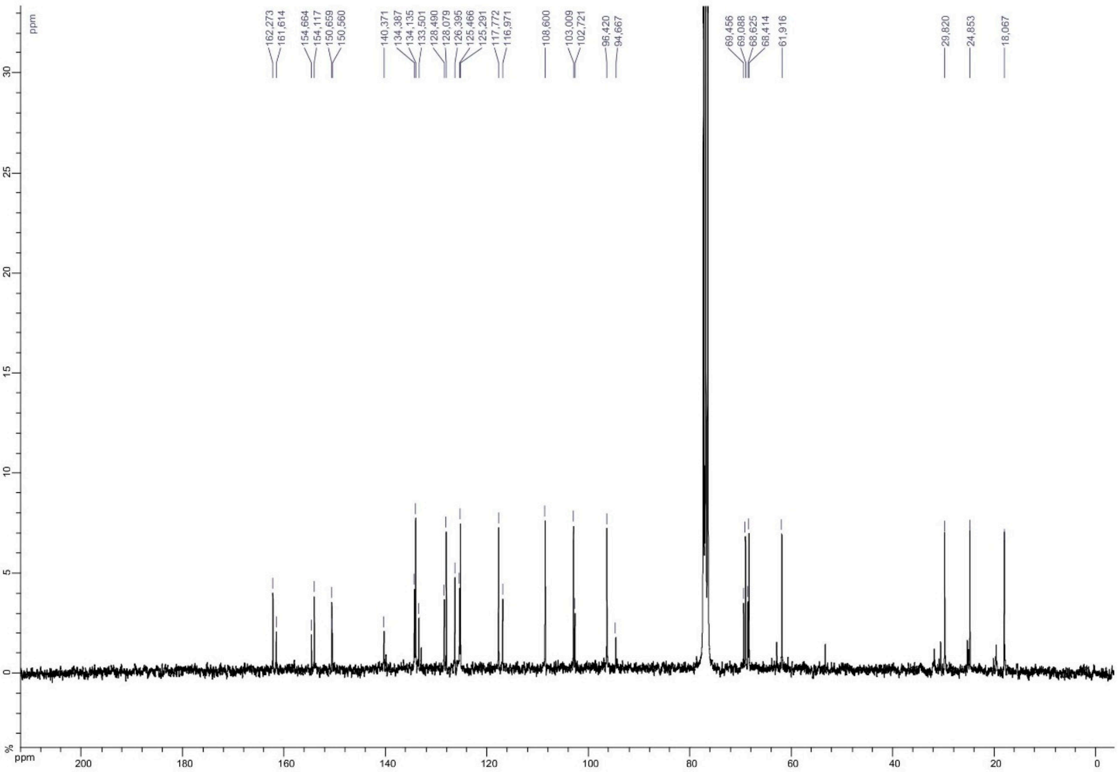

Figure S9. ^{13}C NMR spectrum of **5** ($CDCl_3$, 75 MHz).

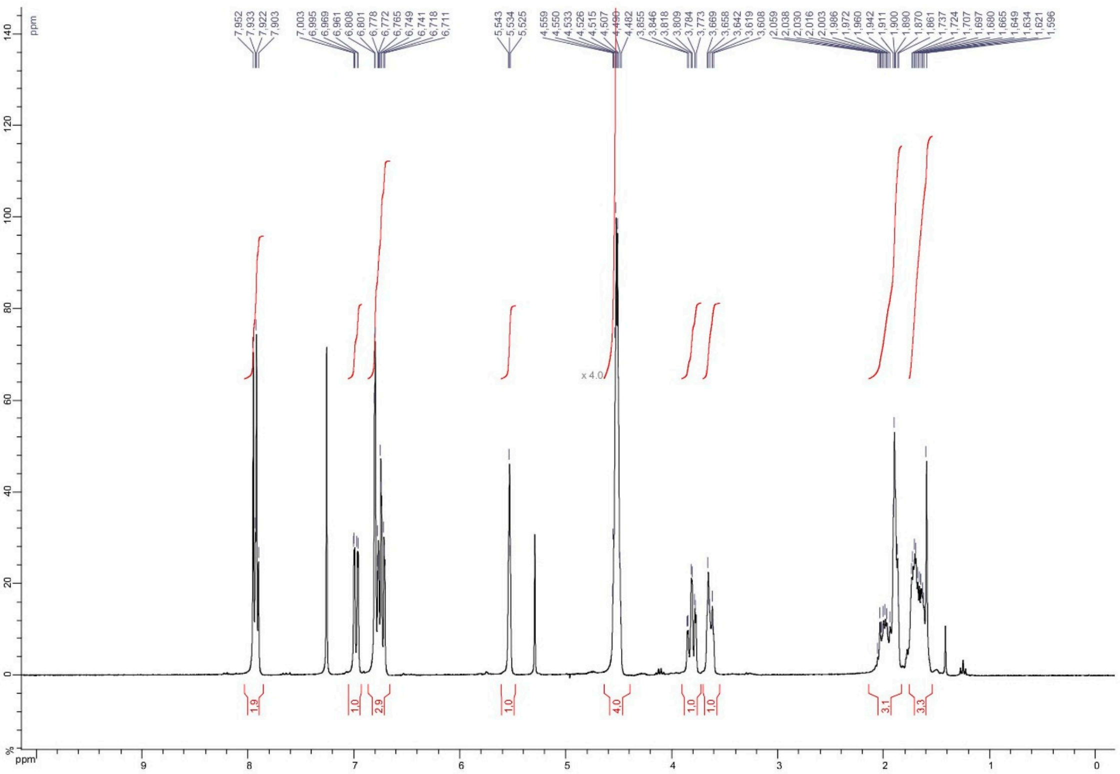

Figure S10. ^{1}H NMR spectrum of **6** ($CDCl_3$, 300 MHz).

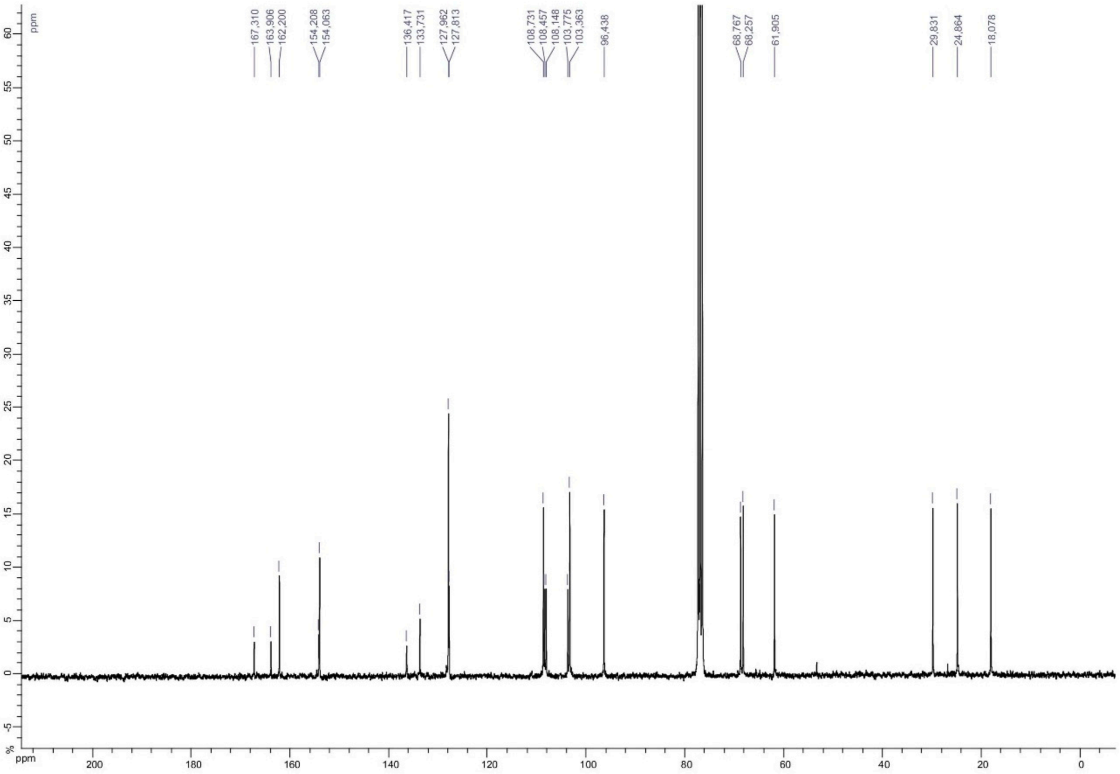

Figure S11. ^{13}C NMR spectrum of **6** ($CDCl_3$, 75 MHz).

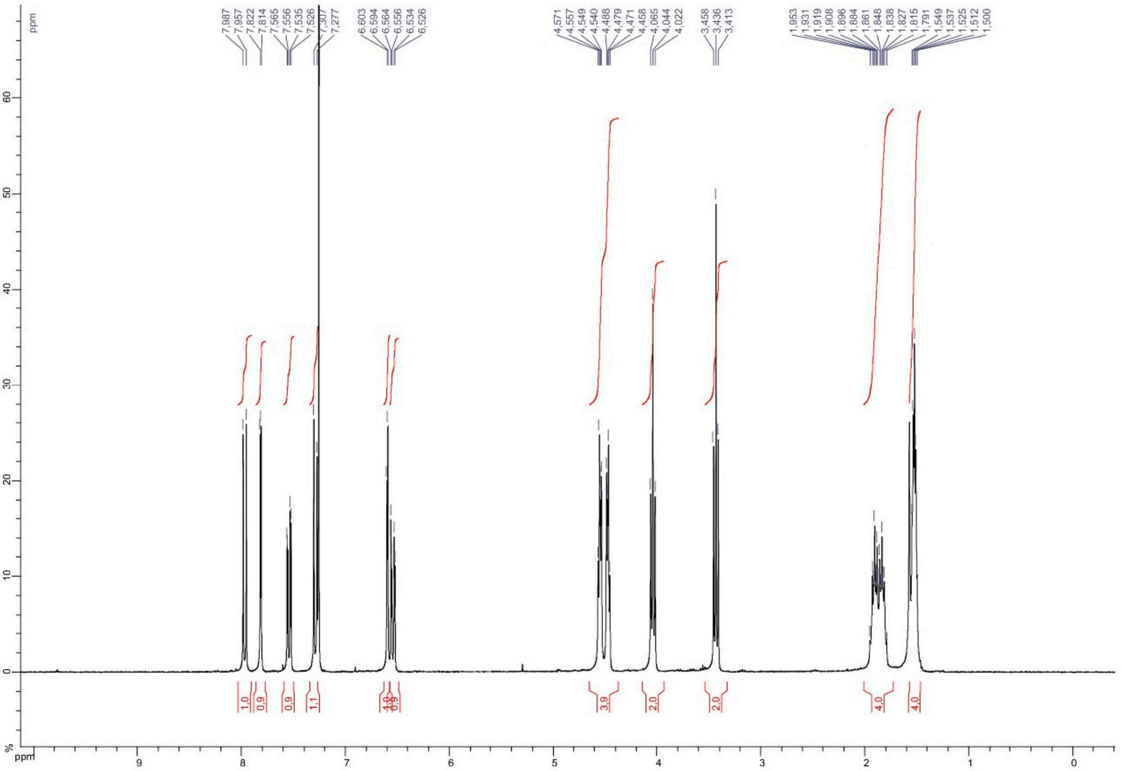

Figure S12. ^{1}H NMR spectrum of **7** ($CDCl_3$, 300 MHz).

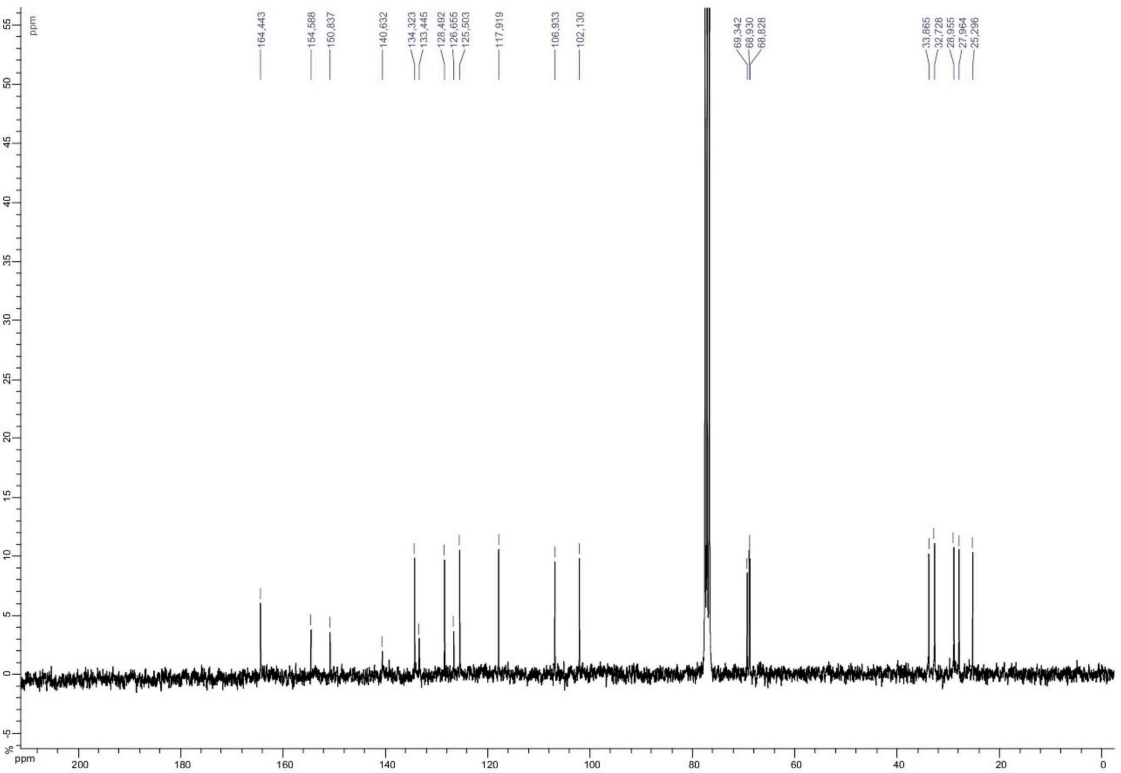

Figure S13. ^{13}C NMR spectrum of **7** ($CDCl_3$, 75 MHz).

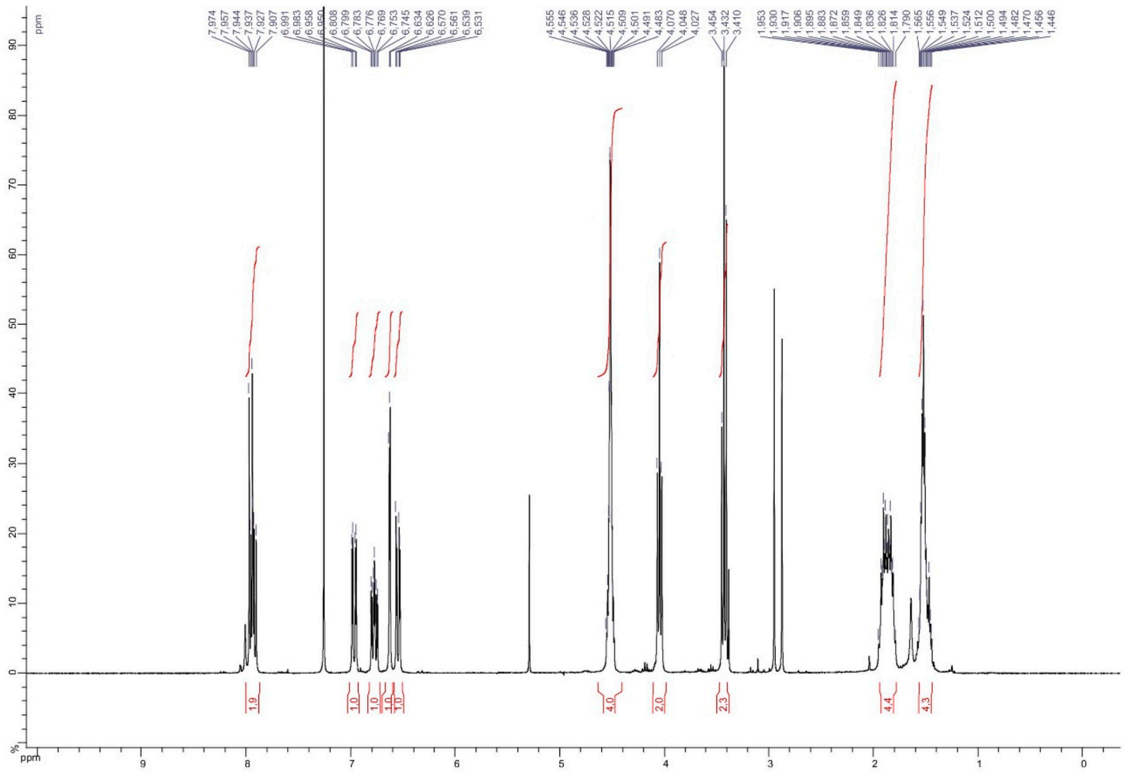

Figure S14. ^{1}H NMR spectrum of **8** ($CDCl_3$, 300 MHz).

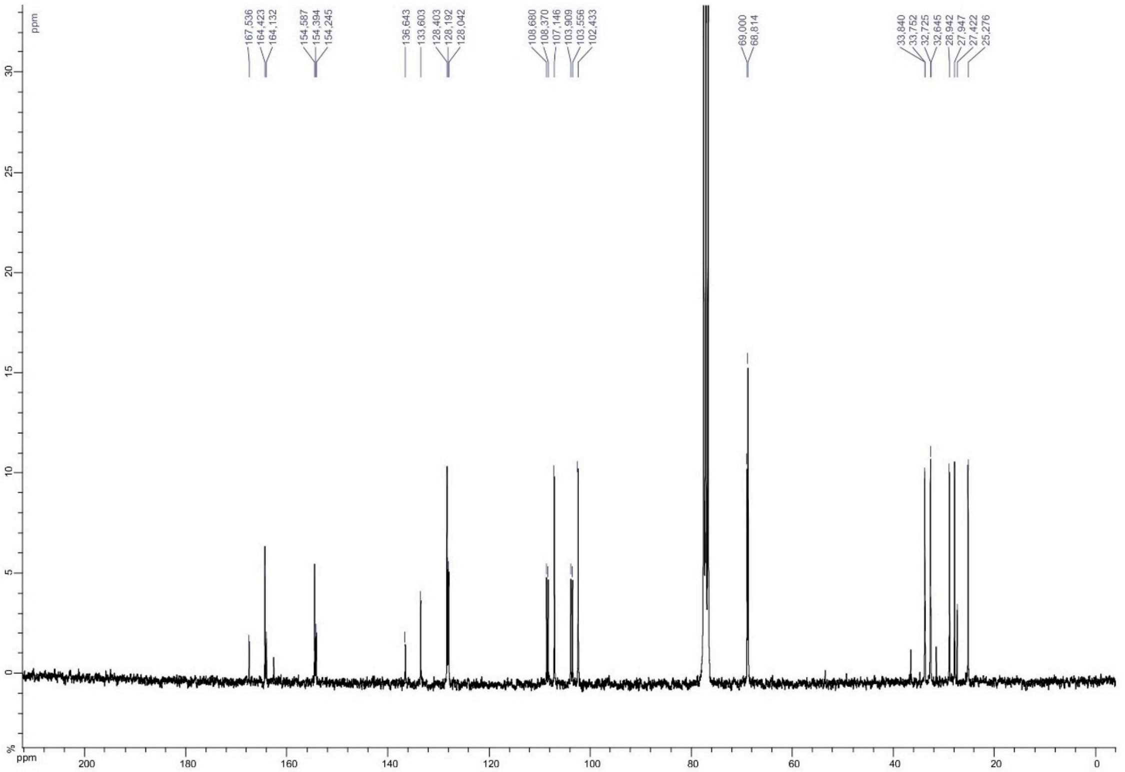

Figure S15. ^{13}C NMR spectrum of **8** ($CDCl_3$, 75 MHz).

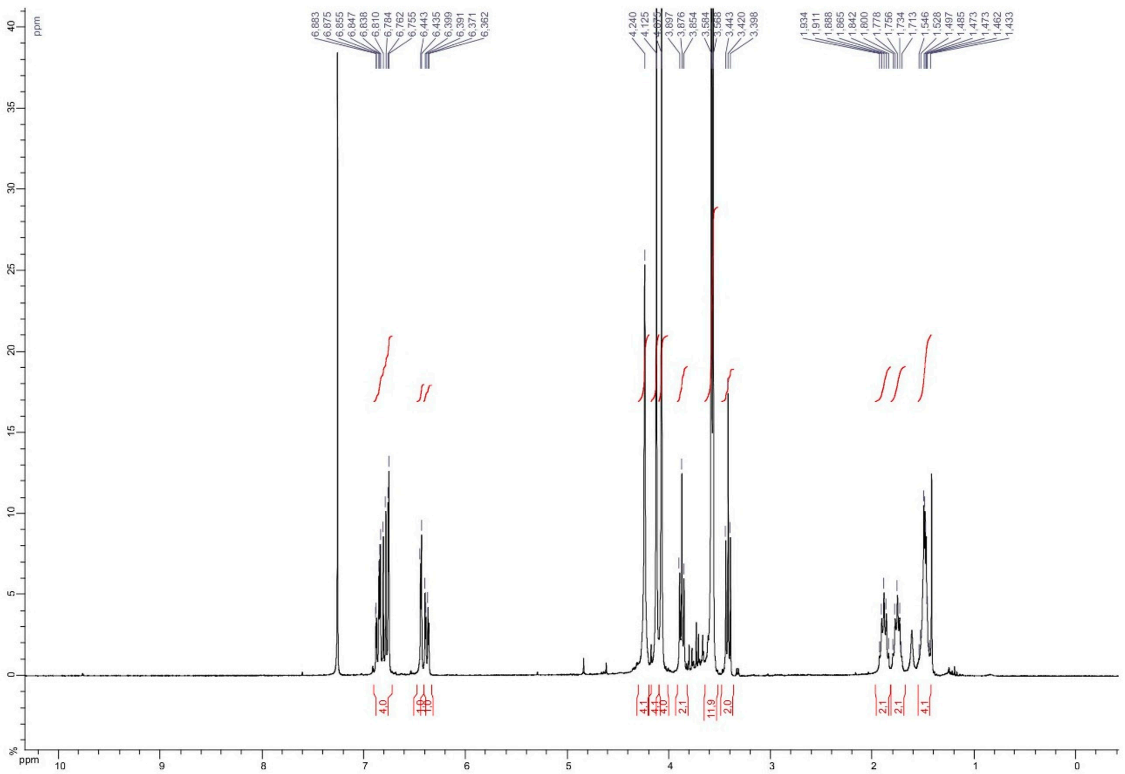

Figure S16. ^{1}H NMR spectrum of **9** ($CDCl_3$, 300 MHz).

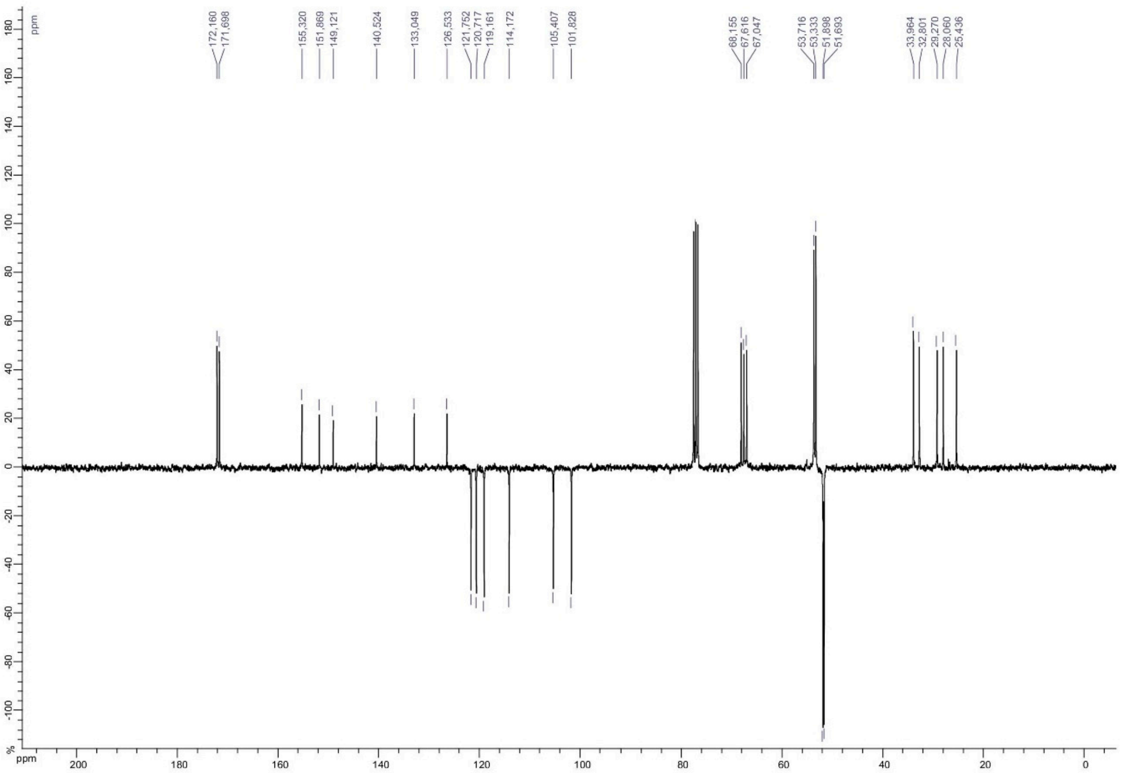

Figure S17. ^{13}C NMR (JMod) spectrum of **9** ($CDCl_3$, 75 MHz.

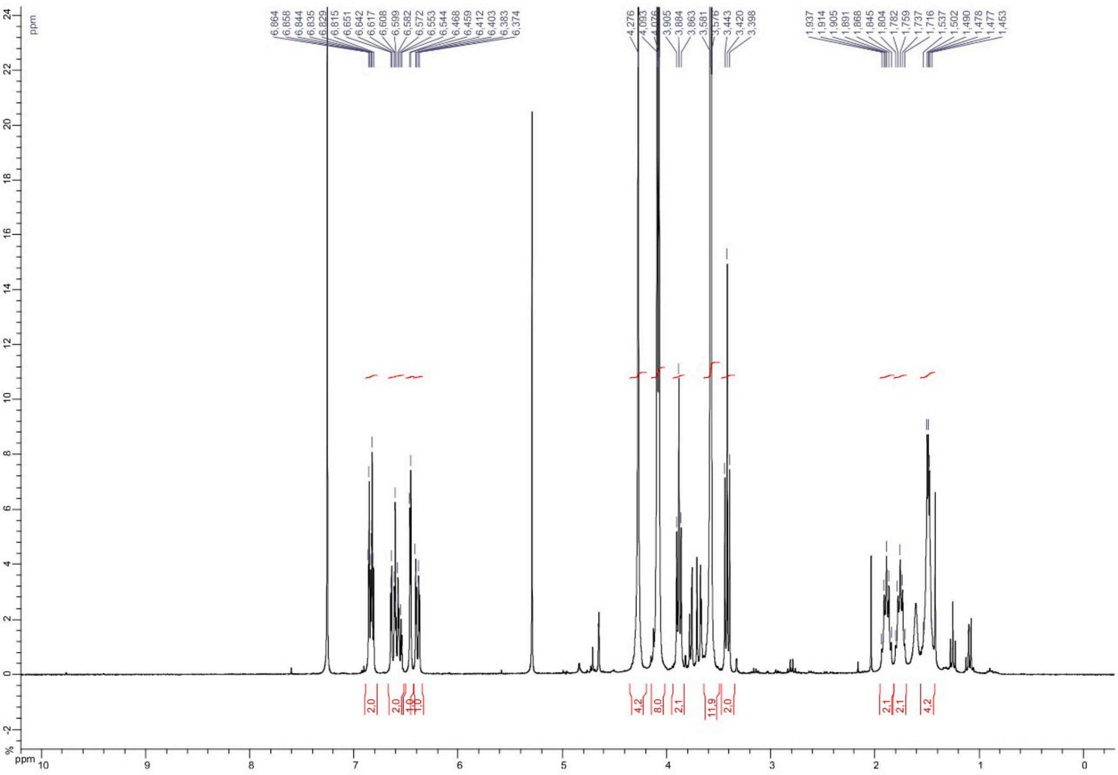

Figure S18. ^{1}H NMR spectrum of **10** ($CDCl_3$, 300 MHz).

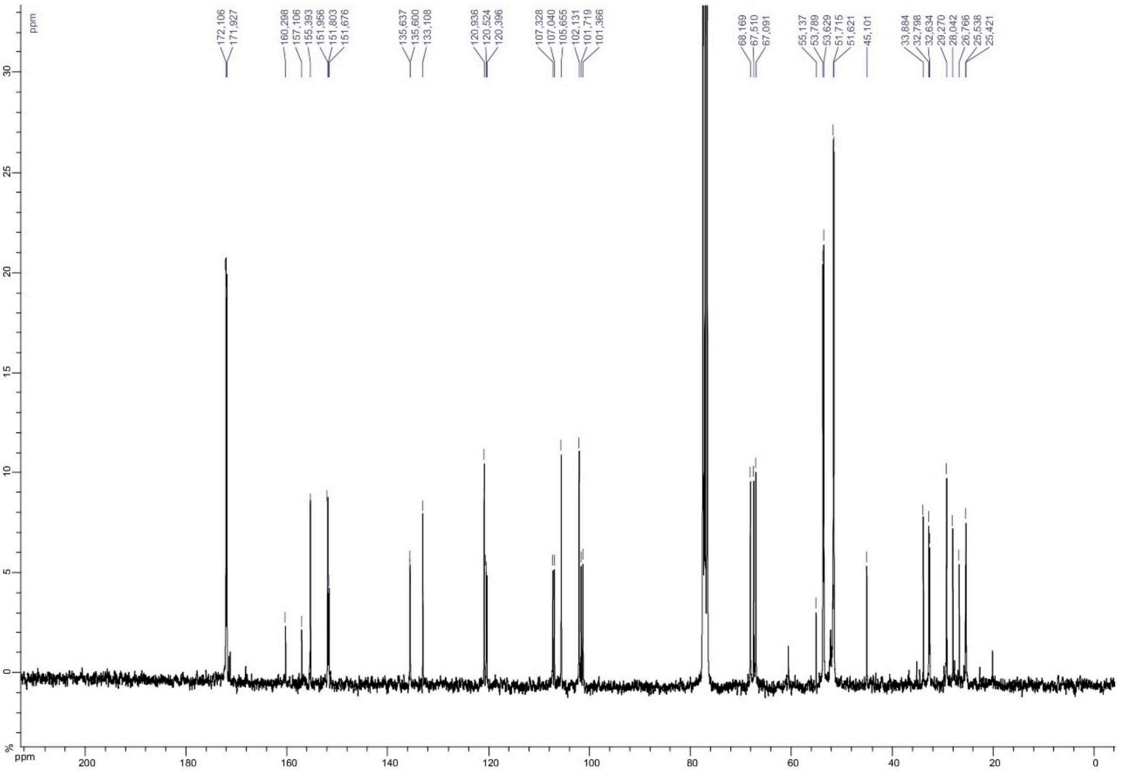

Figure S19. ^{13}C NMR spectrum of **10** ($CDCl_3$, 75 MHz).

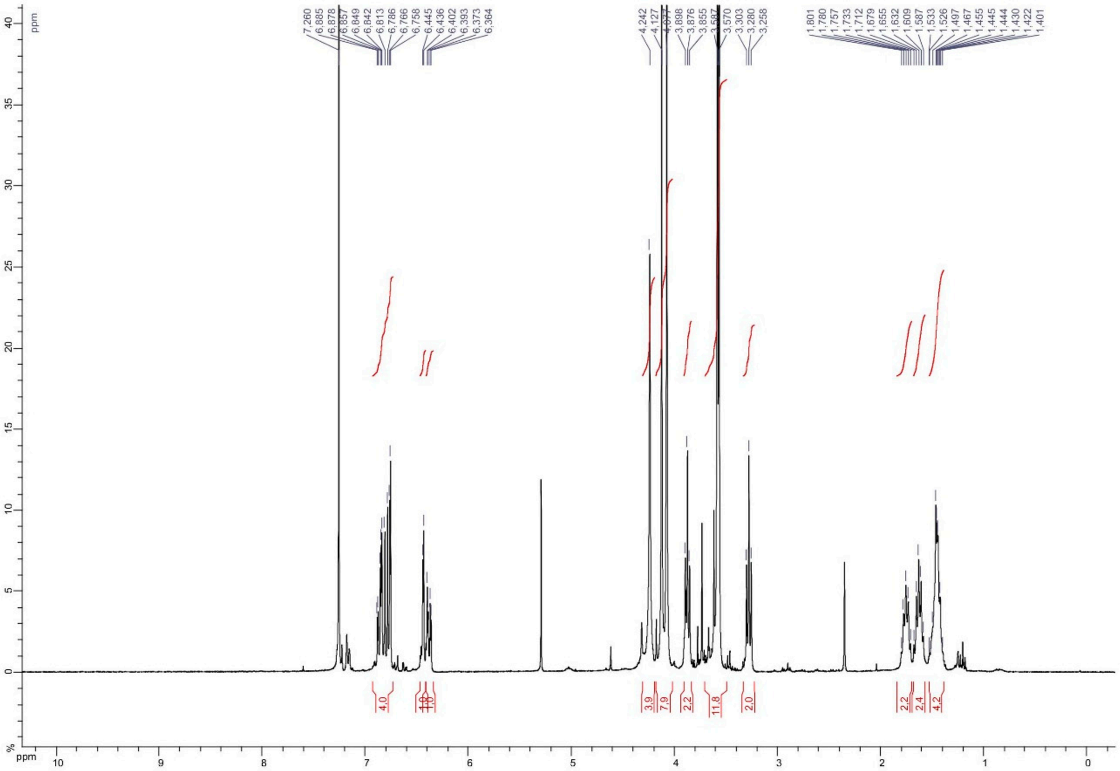

Figure S20. ^{1}H NMR spectrum of **11** ($CDCl_3$, 300 MHz).

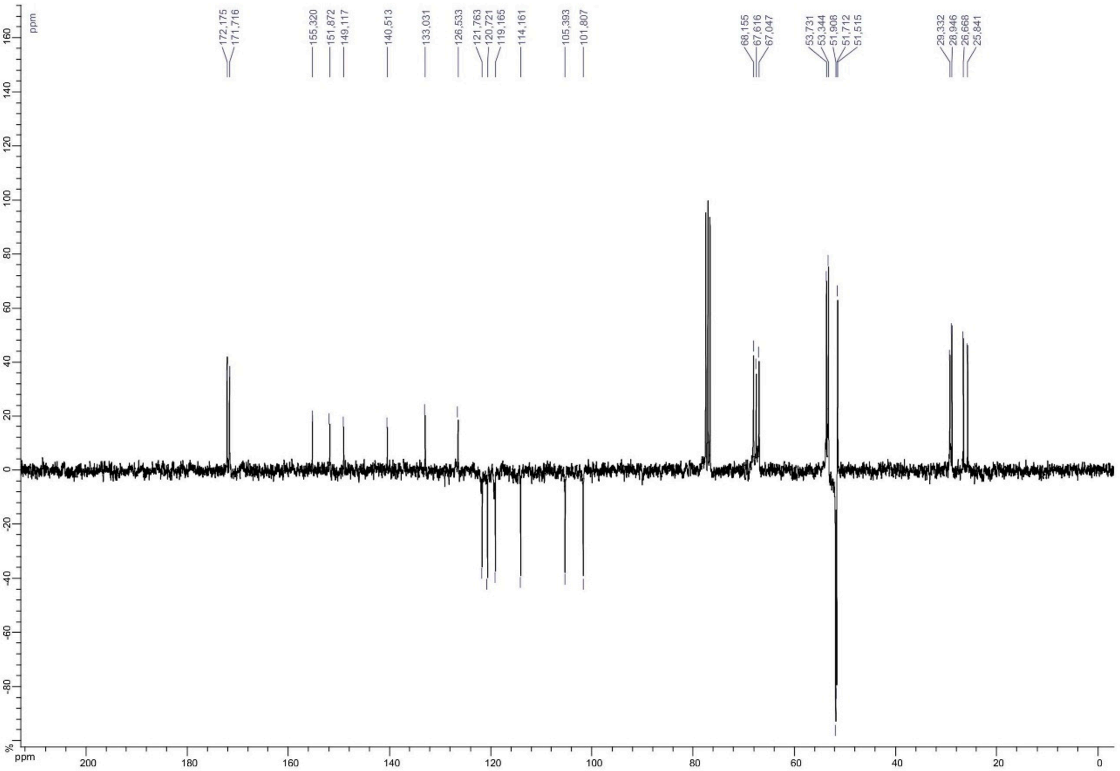

Figure S21. ^{13}C NMR (JMod) spectrum of **11** ($CDCl_3$, 75 MHz).

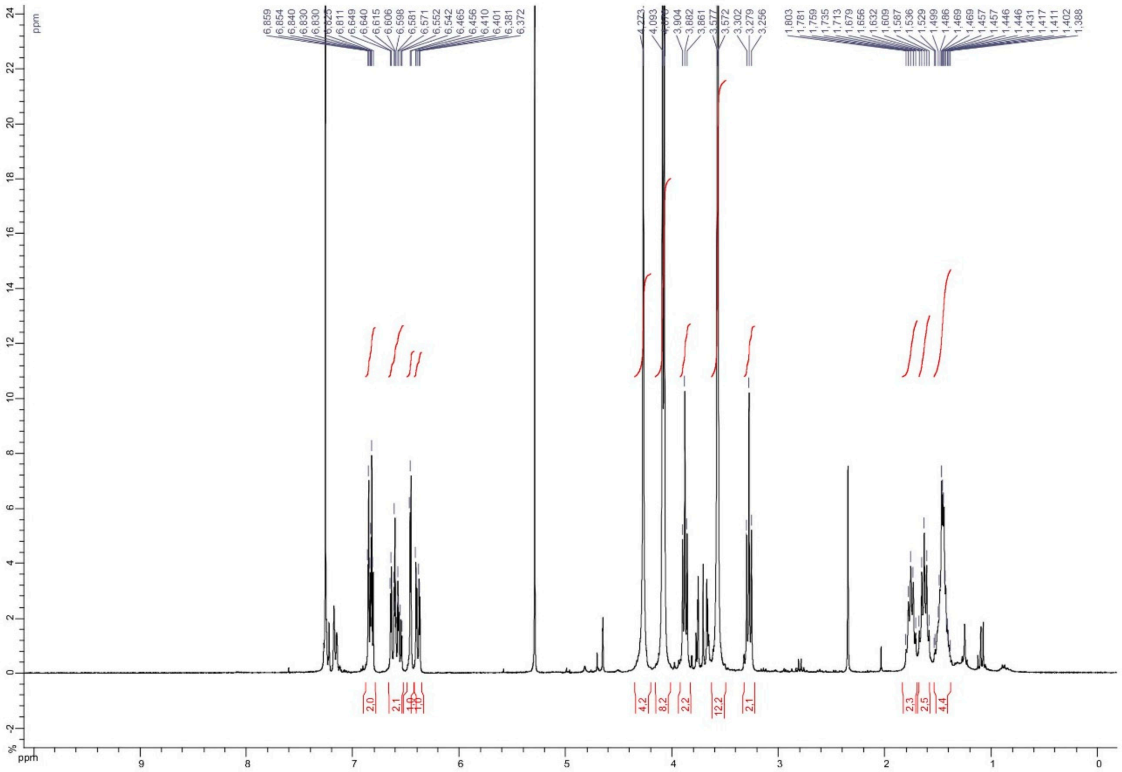

Figure S22. ^{1}H NMR spectrum of **12** ($CDCl_3$, 300 MHz).

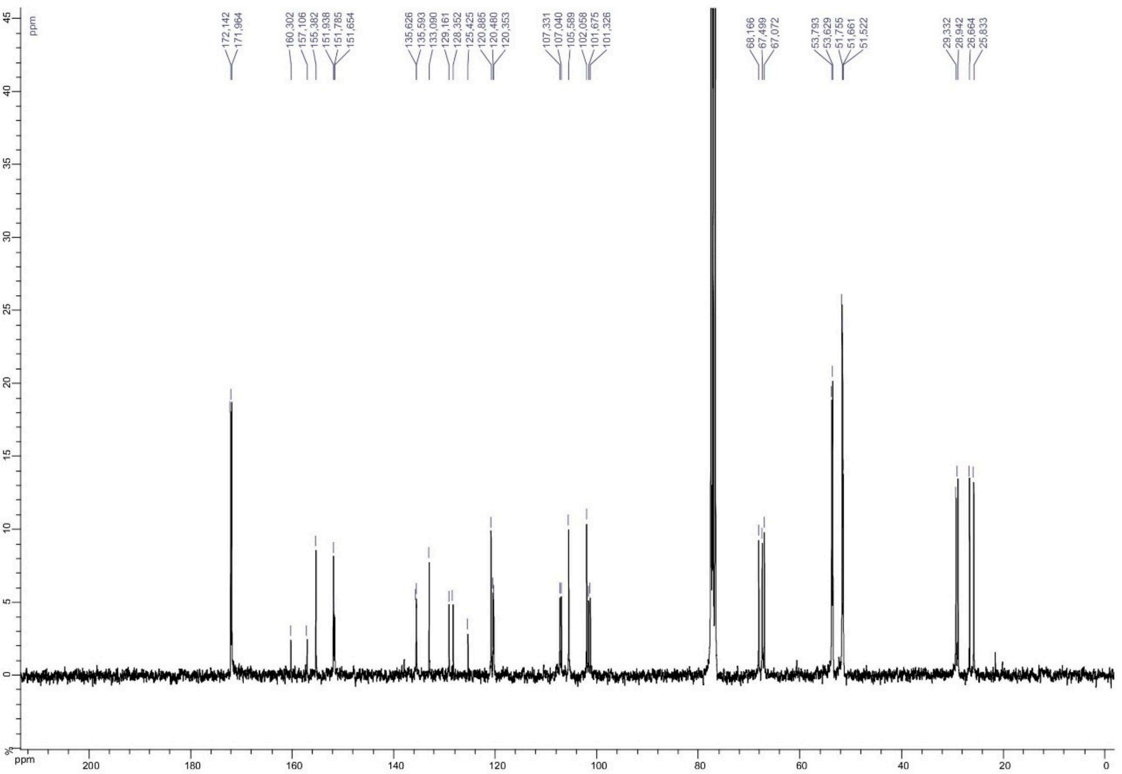

Figure S23. ^{13}C NMR spectrum of **12** ($CDCl_3$, 75 MHz).

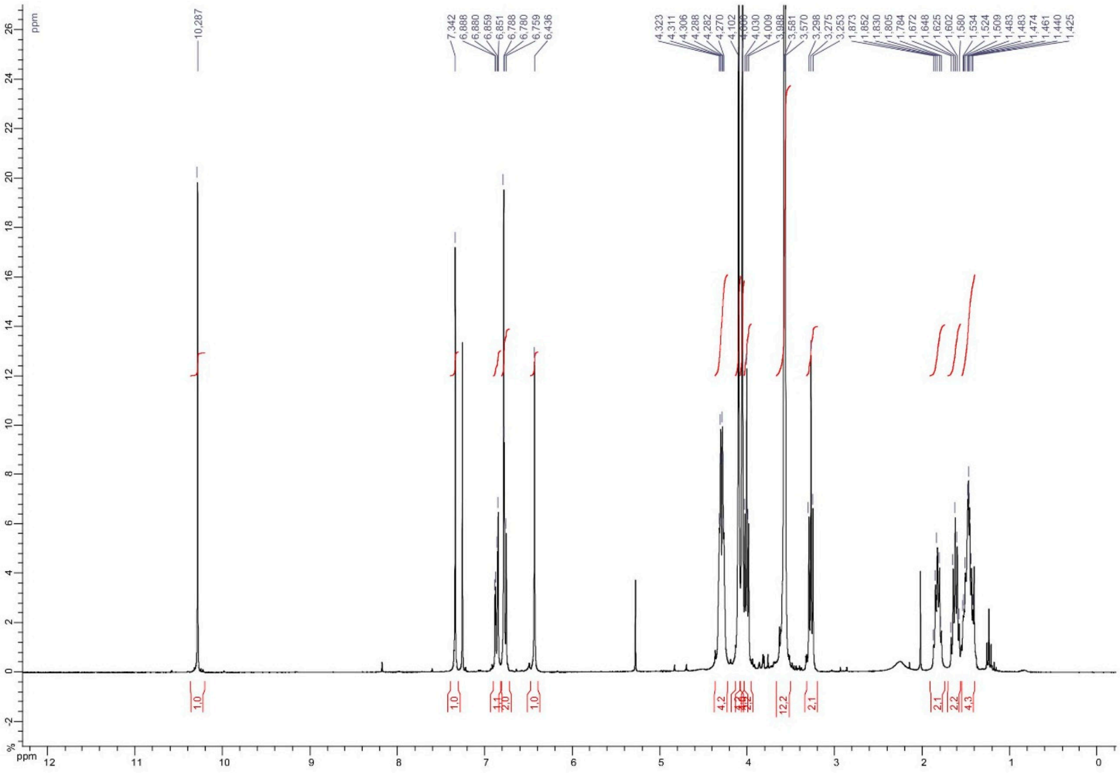

Figure S24. ^{1}H NMR spectrum of **13** ($CDCl_3$, 300 MHz).

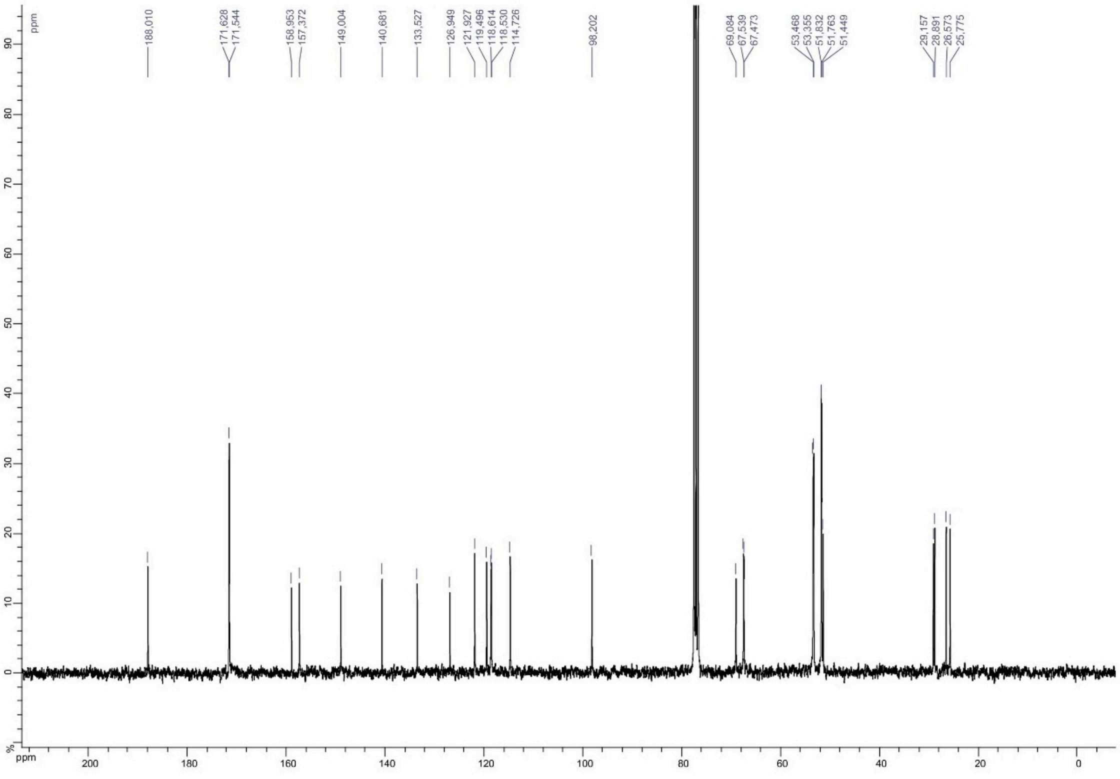

Figure S25. ^{13}C NMR spectrum of **13** ($CDCl_3$, 75 MHz).

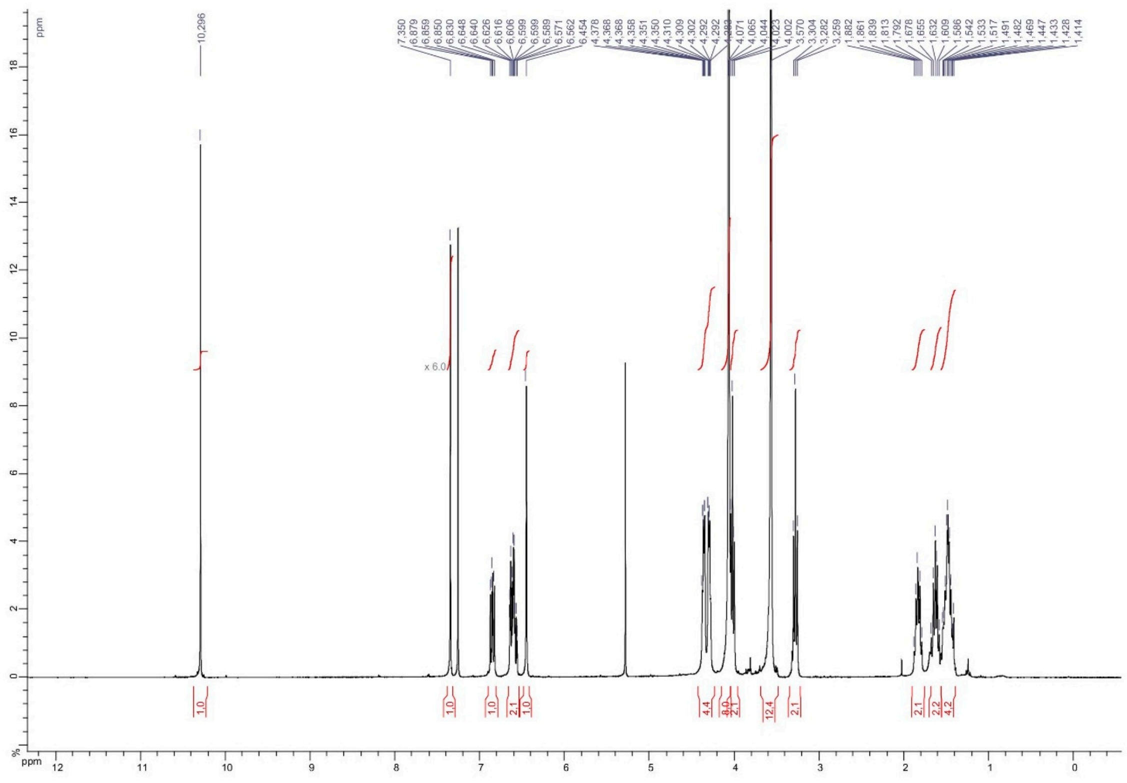

Figure S26. ^{1}H NMR spectrum of **14** ($CDCl_3$, 300 MHz).

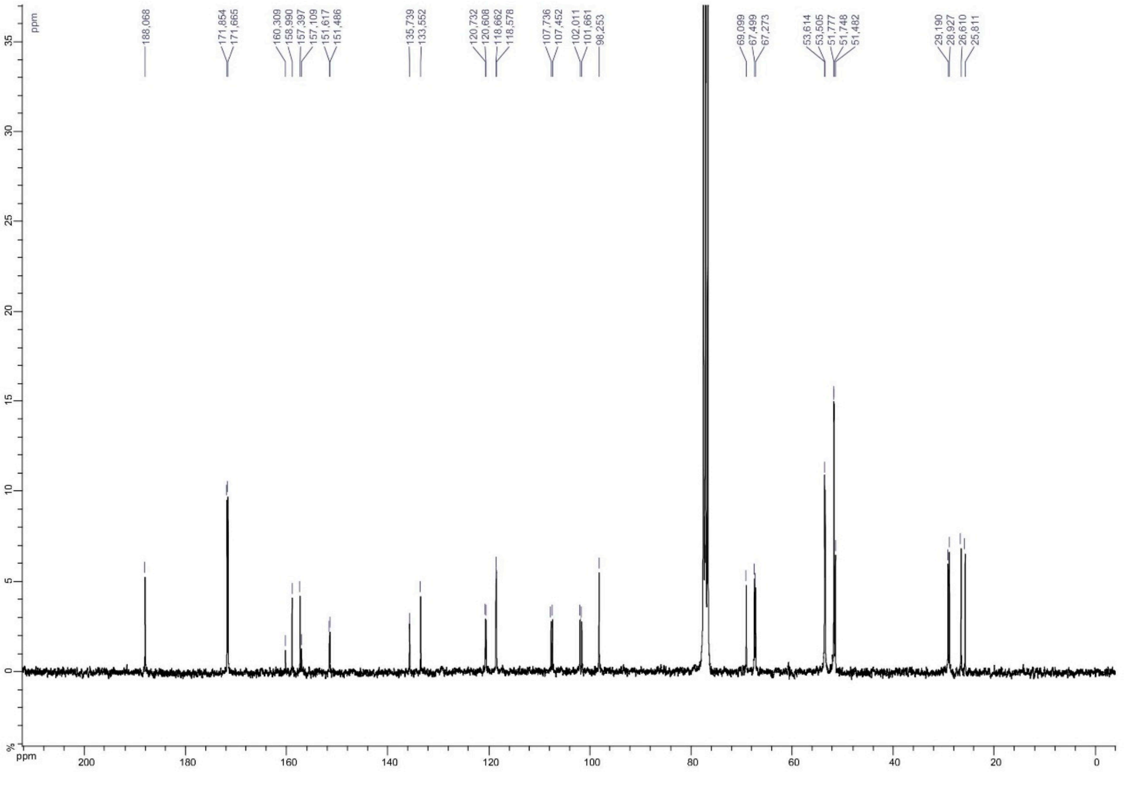

Figure S27. ^{13}C NMR spectrum of **14** ($CDCl_3$, 75 MHz).

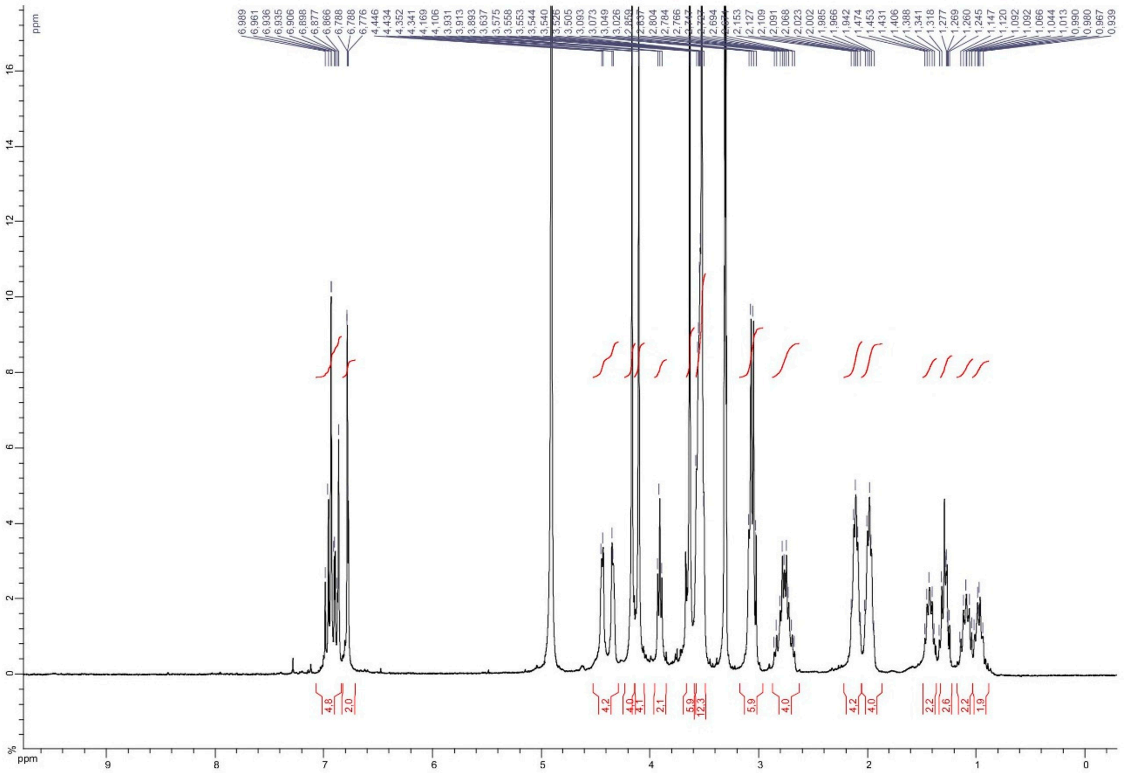

Figure S28. ^{1}H NMR spectrum of **15** (CD_3OD, 300 MHz).

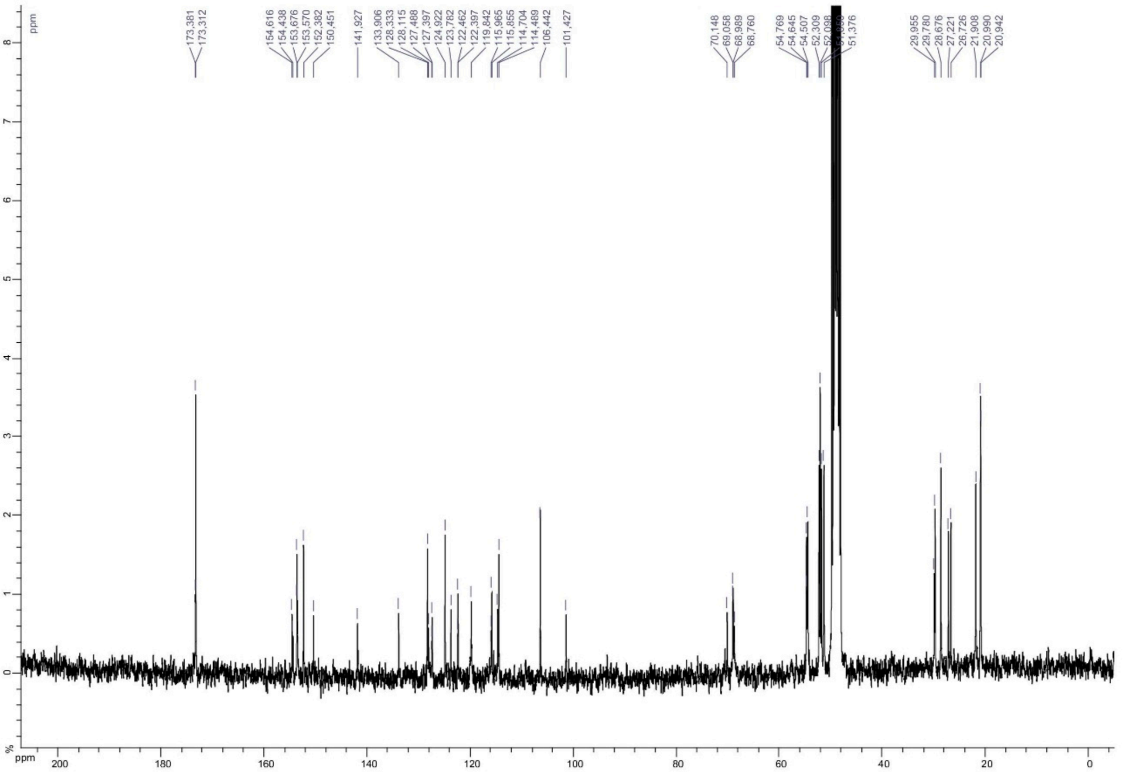

Figure S29. ^{13}C NMR spectrum of **15** (CD_3OD, 75 MHz).

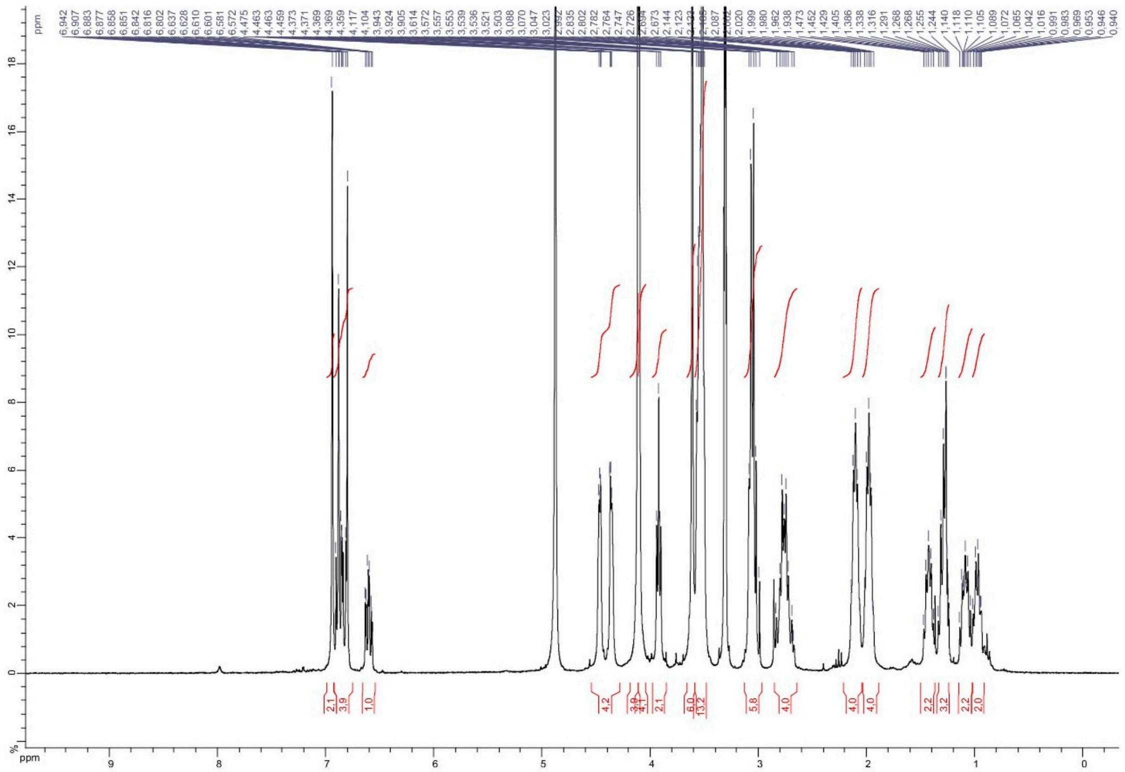

Figure S30. ^{1}H NMR spectrum of **16** (CD_3OD, 300 MHz).

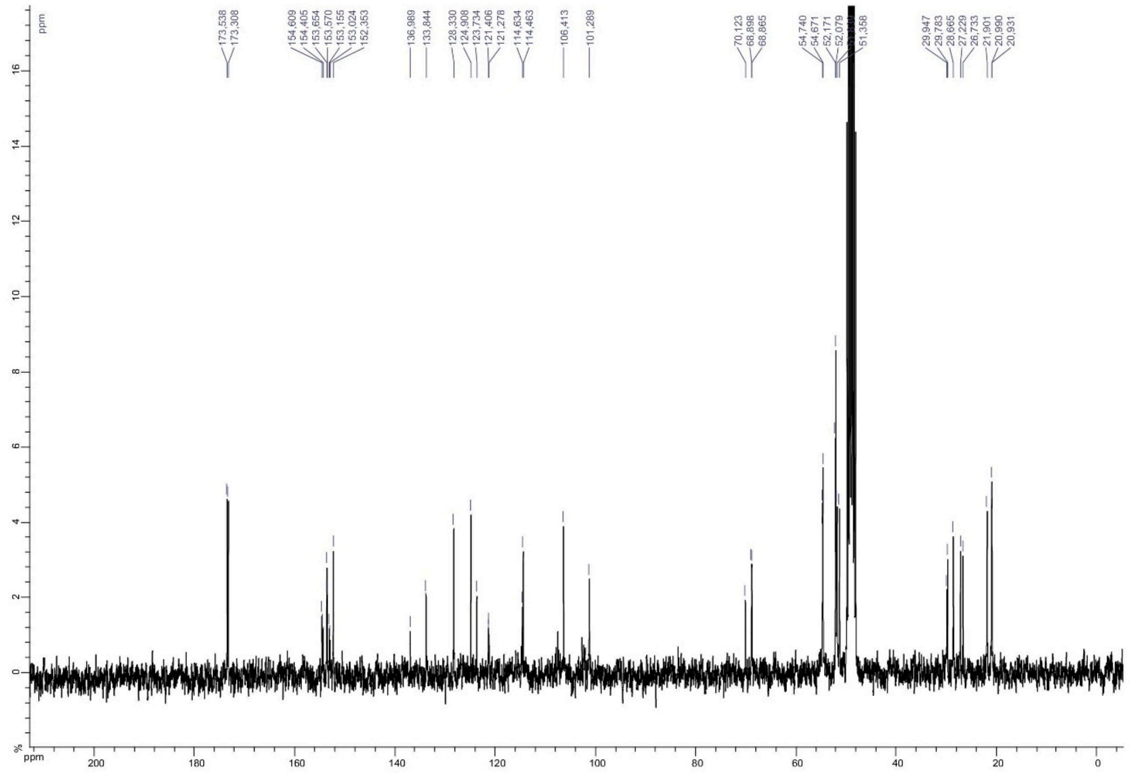

Figure S31. ^{13}C NMR spectrum of **16** (CD_3OD, 75 MHz).

6. Copies of HRMS Spectra

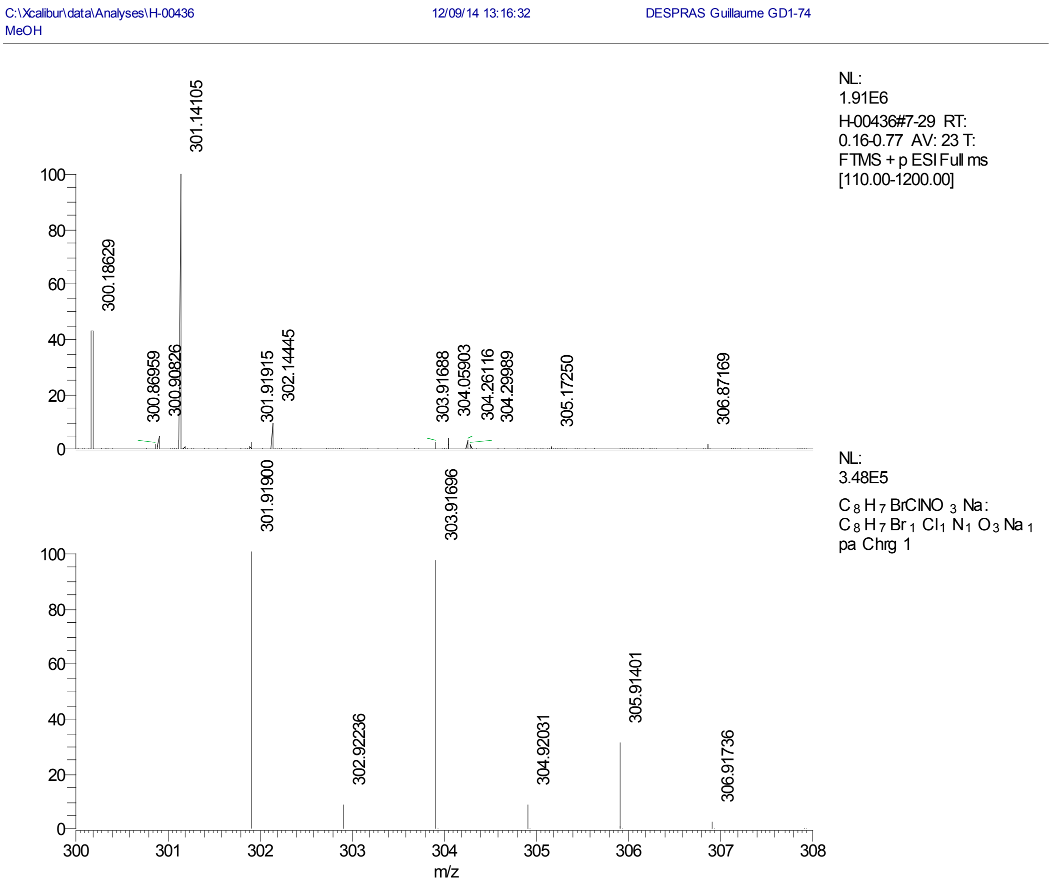

Figure S32. HRMS spectrum of **3**.

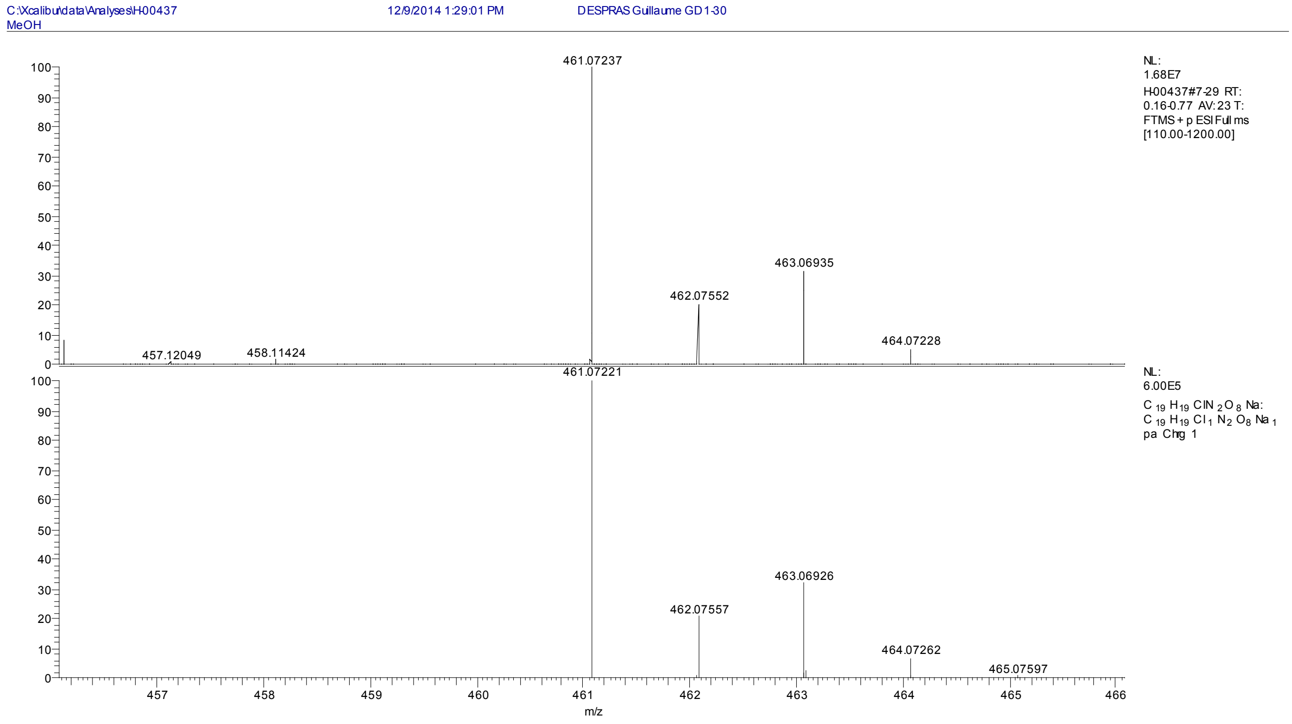

Figure S33. HRMS spectrum of **5**.

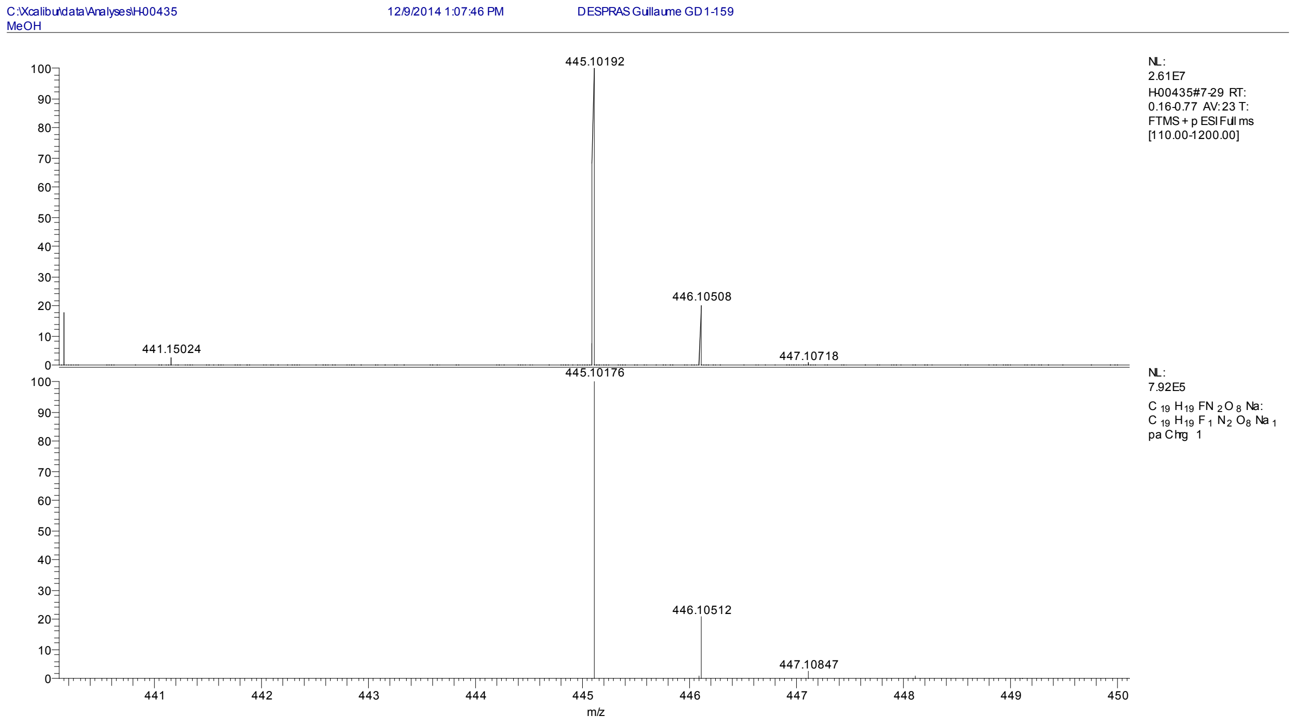

Figure S34. HRMS spectrum of **6**.

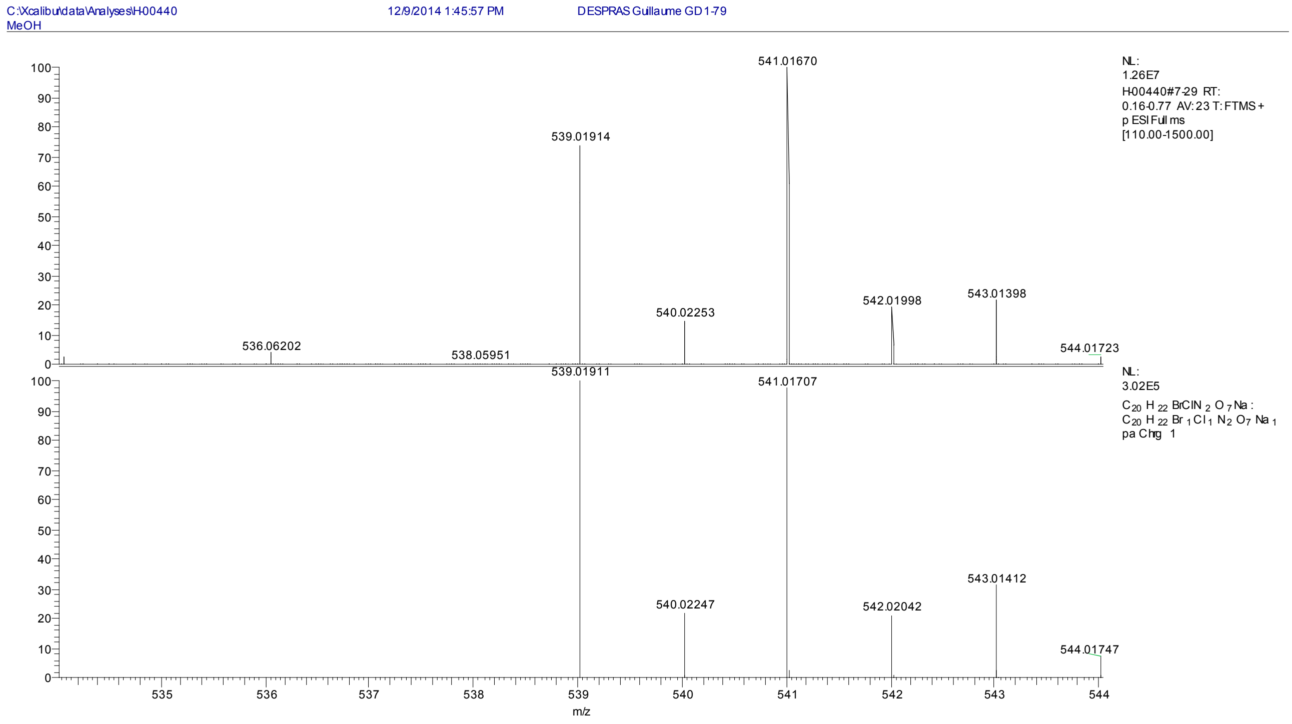

Figure S35. HRMS spectrum of **7**.

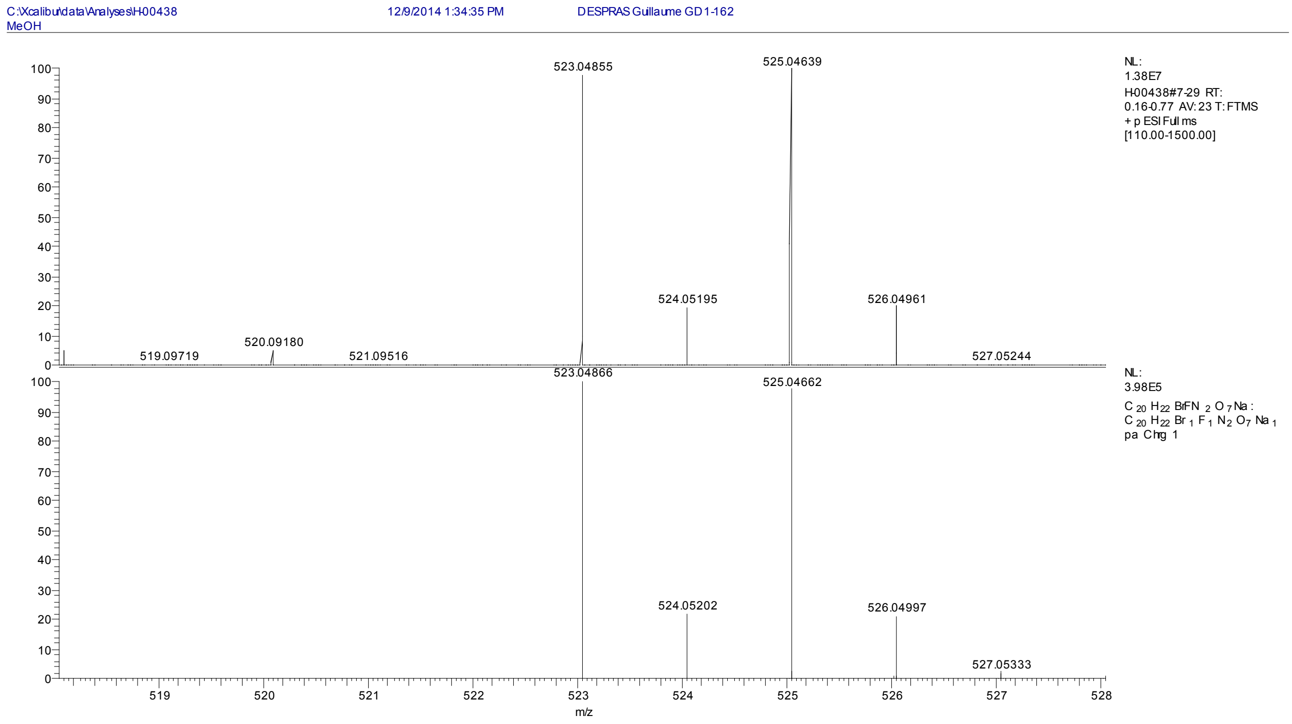

Figure S36. HRMS spectrum of **8**.

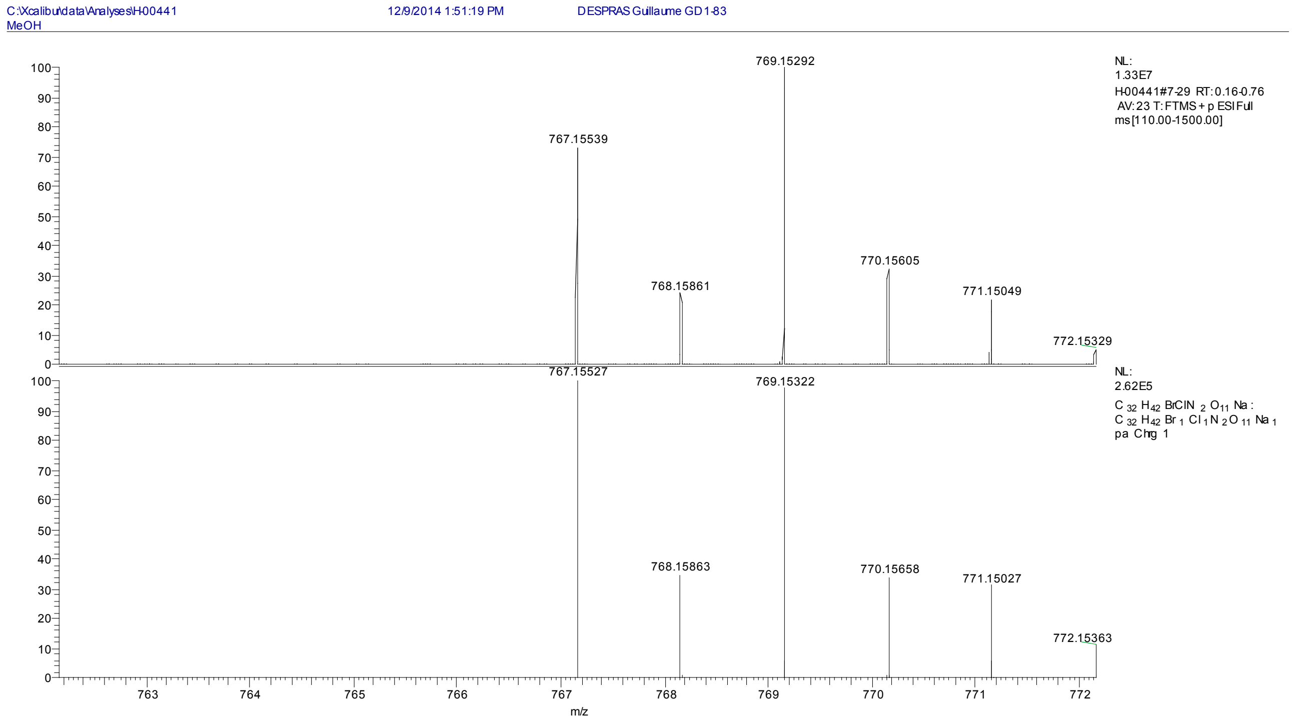

Figure S37. HRMS spectrum of **9**.

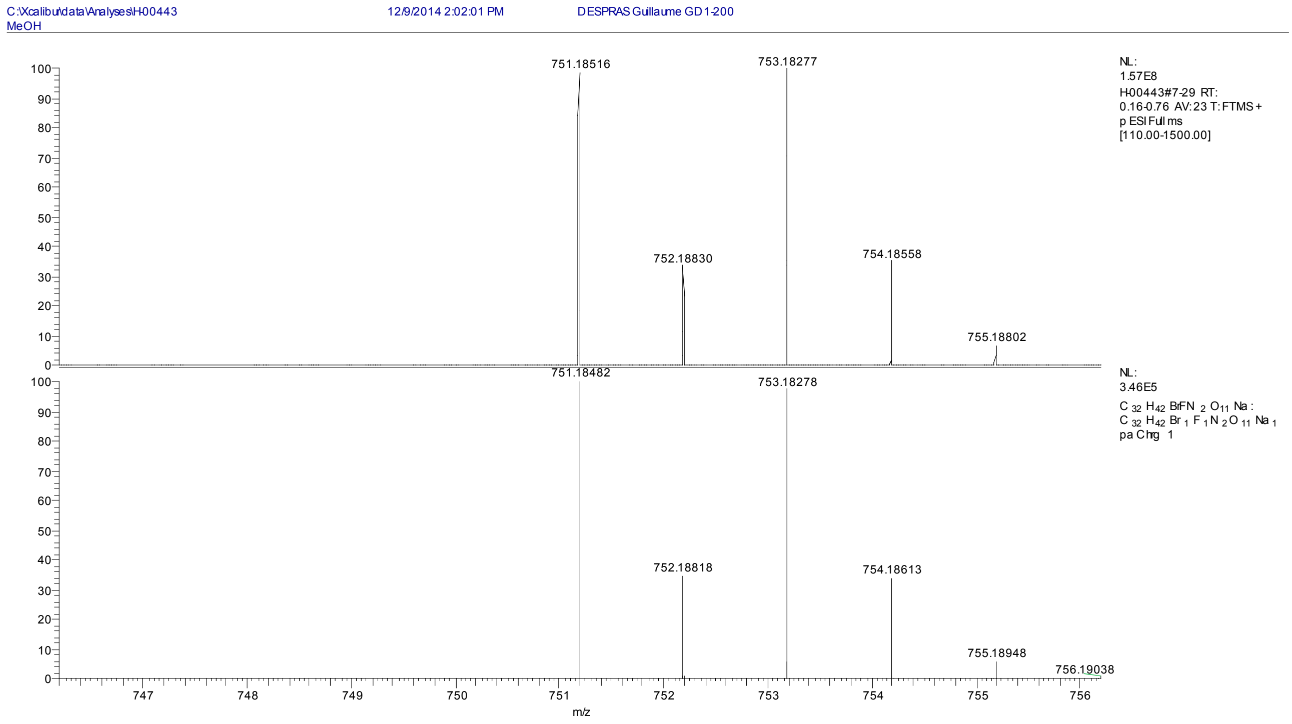

Figure S38. HRMS spectrum of **10**.

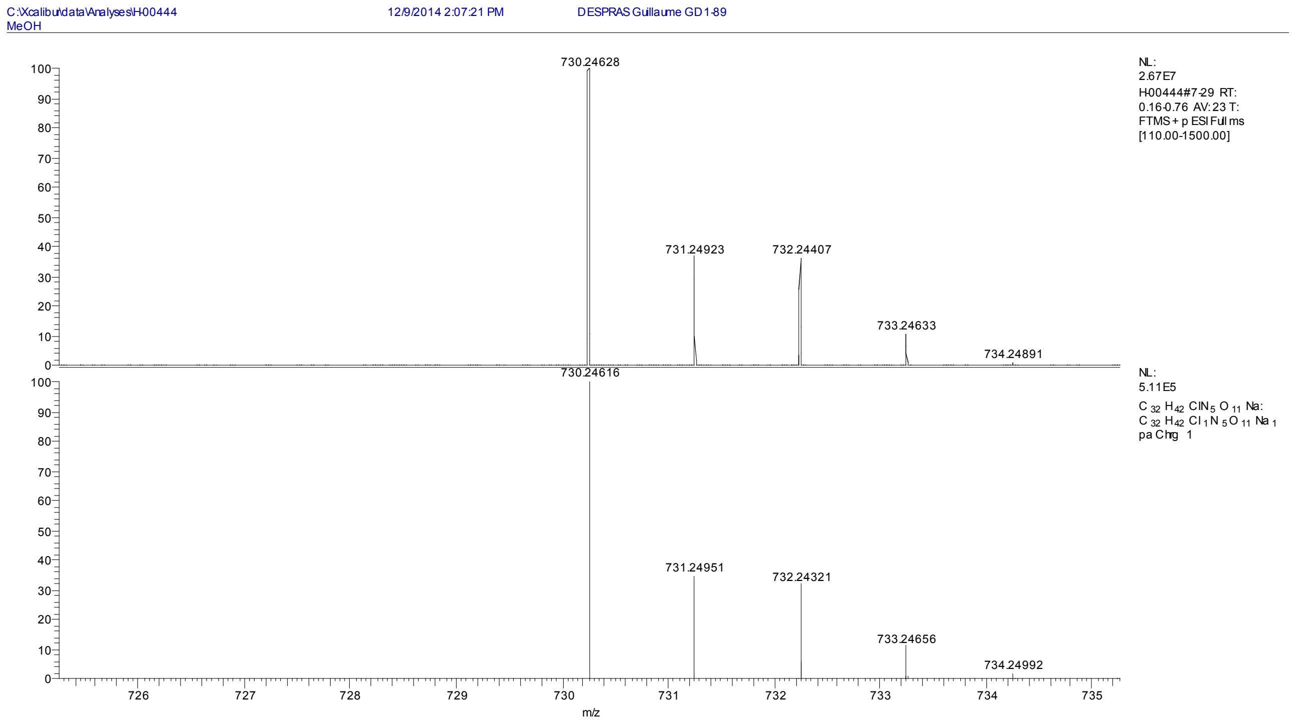

Figure S39. HRMS spectrum of **11**.

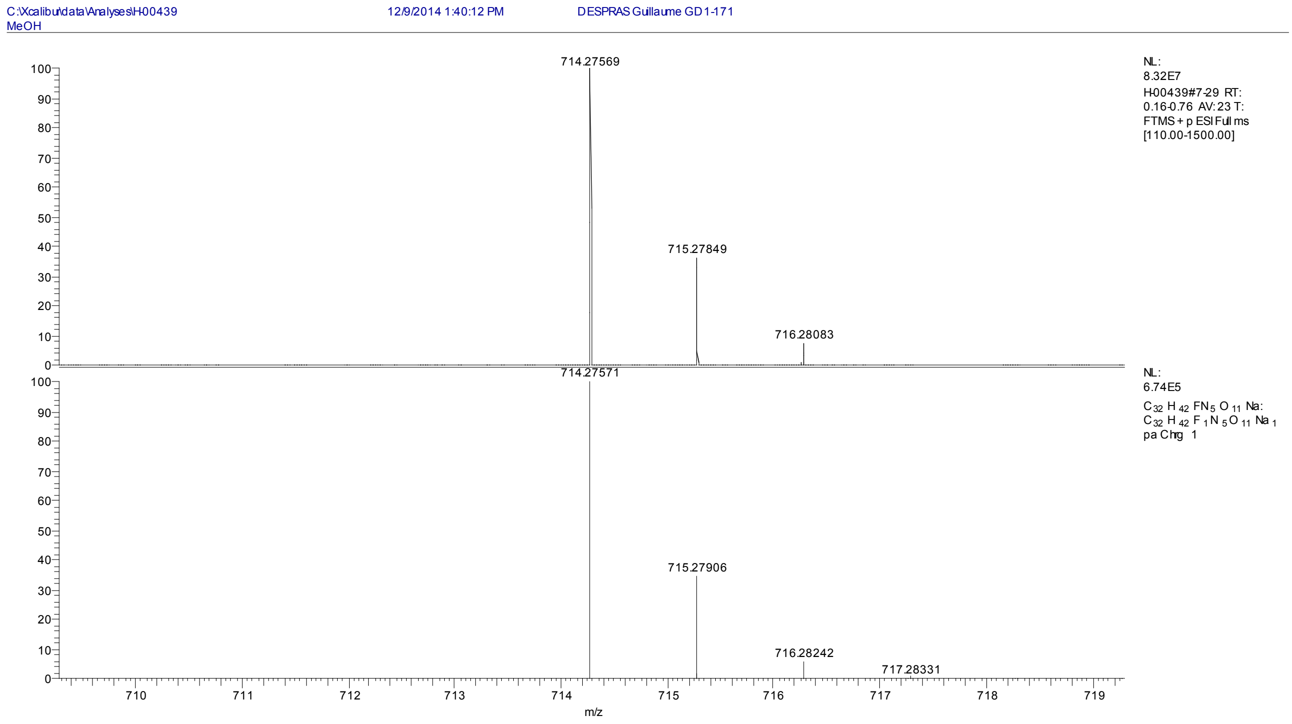

Figure S40. HRMS spectrum of **12**.

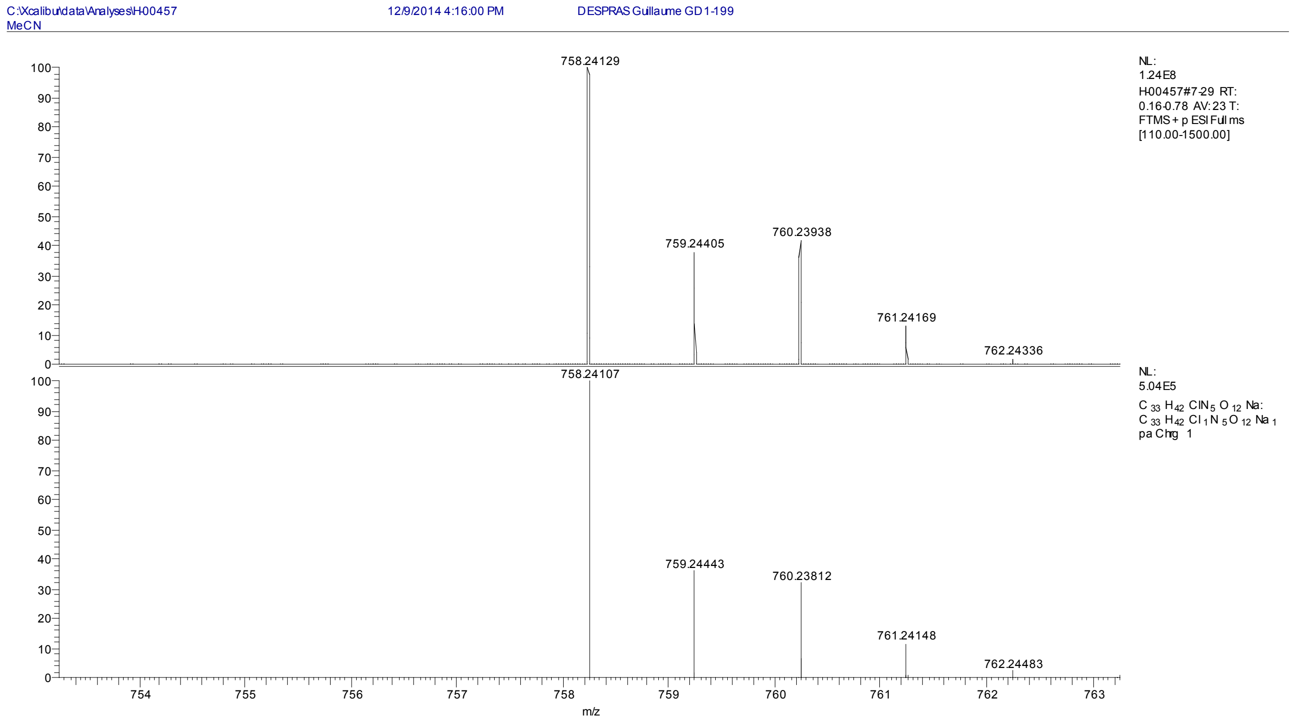

Figure S41. HRMS spectrum of **13**.

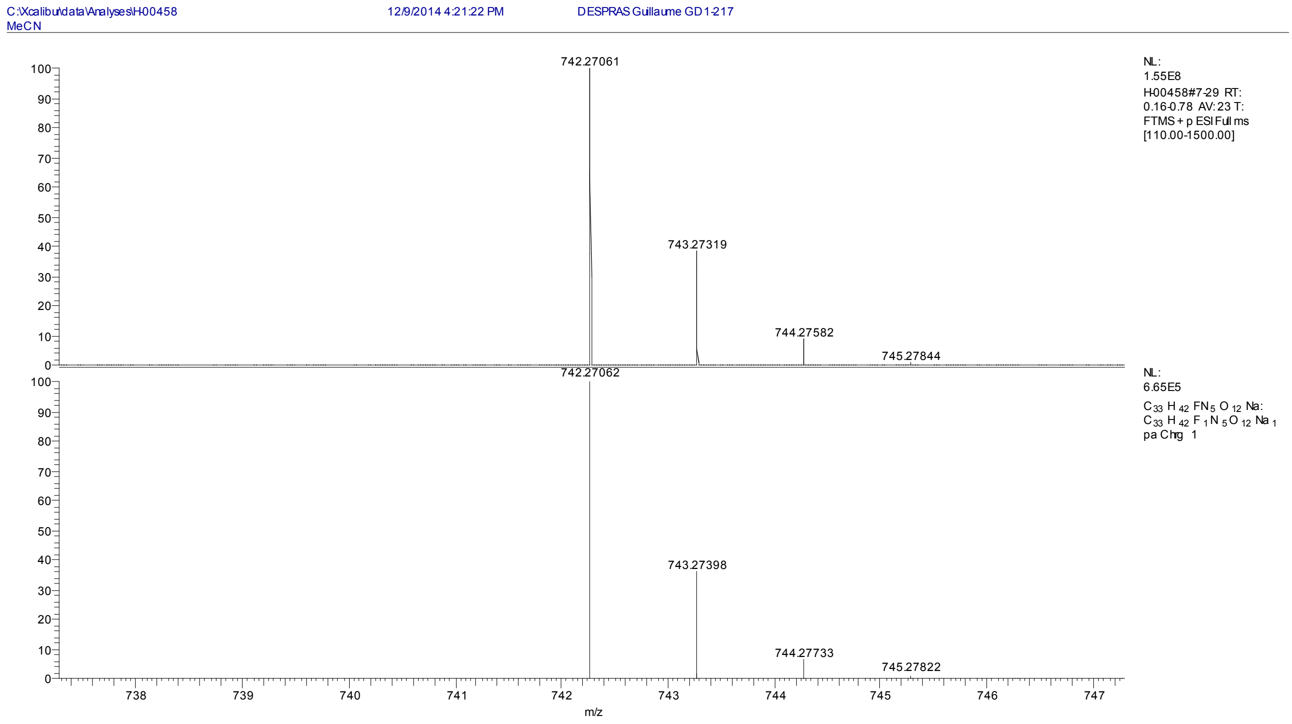

Figure S42. HRMS spectrum of **14**.

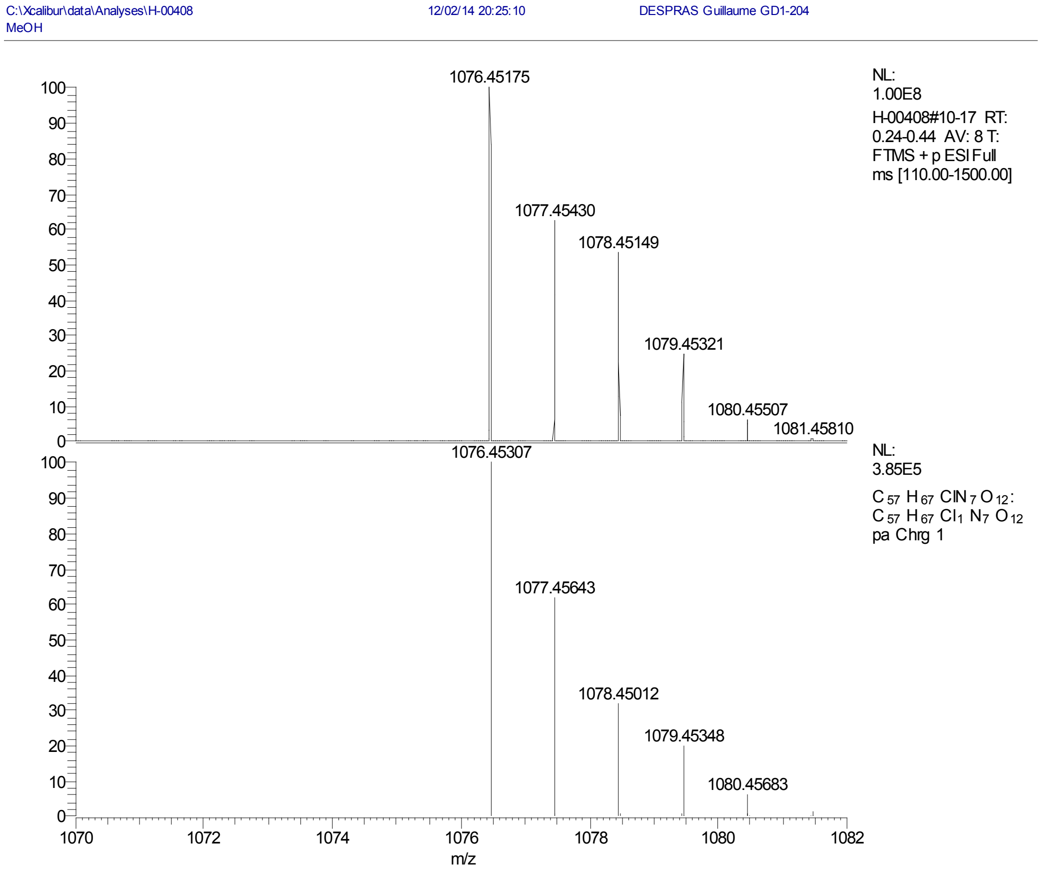

Figure S43. HRMS spectrum of **15**.

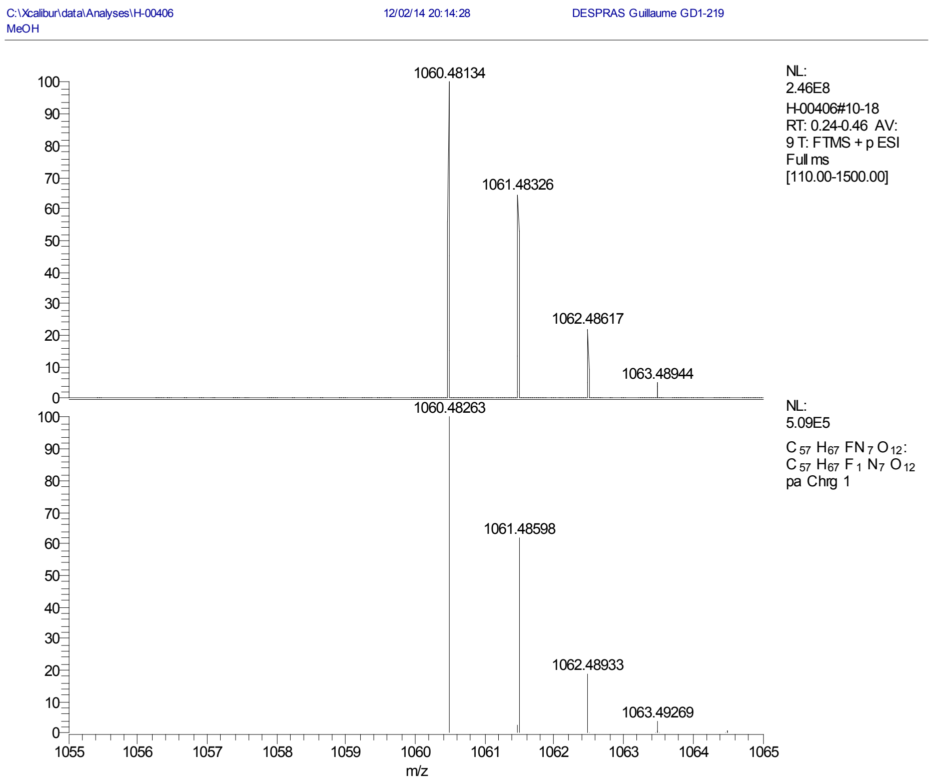

Figure S44. HRMS spectrum of **16**.

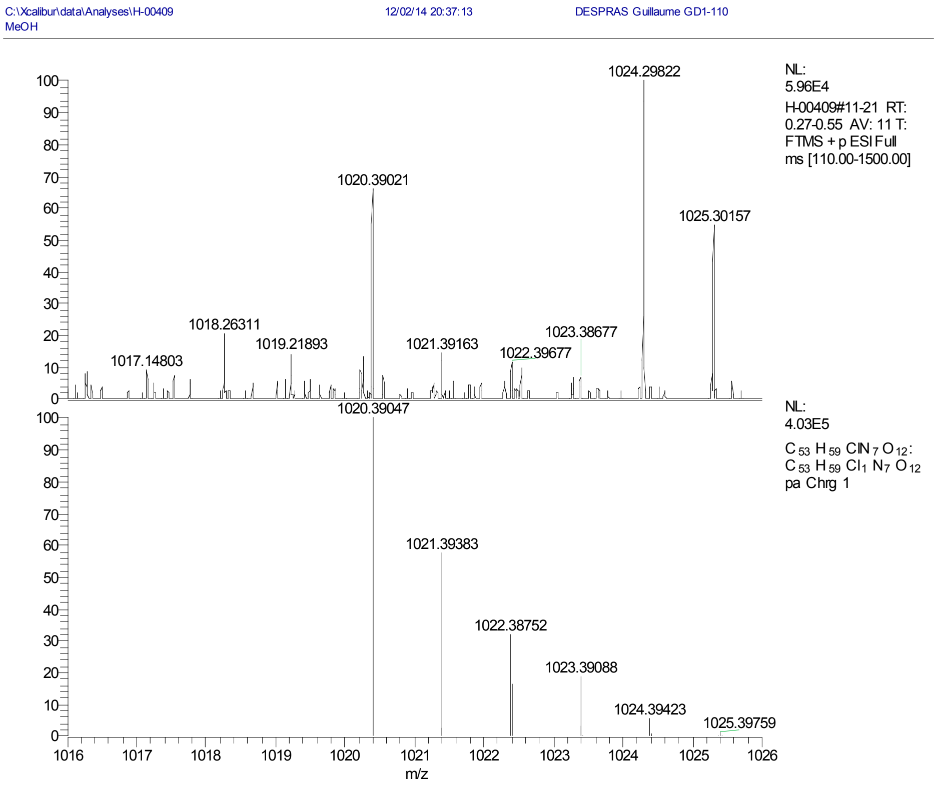

Figure S45. HRMS spectrum of **17**.

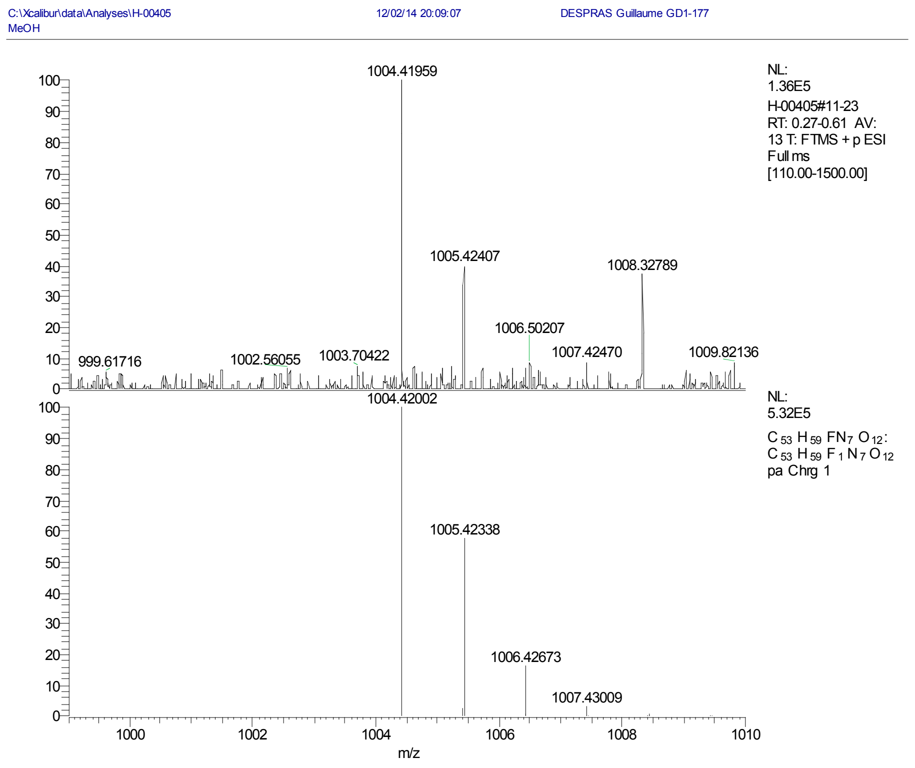

Figure S46. HRMS spectrum of **18**.

References

1. Shi, M.; Cui, S.C. A new method for nitration of phenolic compounds. *Adv. Synth. Catal.* **2003**, *345*, 1197–1202.
2. Luboch, E.; Wagner-Wysiecka, E.; Biernat, J.-F. Chromogenic azocrown ethers with peripheral alkyl, alkoxy, hydroxy or dimethylamino group. *J. Supramol. Chem.* **2002**, *2*, 279–291.
3. Chen, P.; Zhao, Y.; Fink, B.E.; Kim, S.H. Pyrrolotriazine kinase inhibitors. Patent US. 7514435 B2, 7 April 2009.
4. Collot, M.; Wilms, C.D.; Bentkhayet, A.; Marcaggi, P.; Couchman, K.; Charpak, S.; Dieudonné, S.; Haüsser, M.; Feltz, A.; Mallet, J.-M. CaRuby-Nano: A novel high affinity calcium probe for dual color imaging. *eLife* **2015**, *4*, doi:10.7554/eLife.05808.

© 2015 by the authors; licensee MDPI, Basel, Switzerland. This article is an open access article distributed under the terms and conditions of the Creative Commons Attribution license (http://creativecommons.org/licenses/by/4.0/).
